# History of Neurosurgery in Malaysia: The Past, Present and Future

**DOI:** 10.21315/mjms2021.28.6.13

**Published:** 2021-12-22

**Authors:** Fadzli Cheah ABDULLAH, Zaitun ZAKARIA, Hari Chandran THAMBINAYAGAM, Regunath KANDASAMY, Azmi ALIAS, Azizi ABU BAKAR, Albert Sii Hieng WONG, Pulivendhan SELLAMUTHU, Rahmat HARUN, Saiful Azli MAT NAYAN, Azman RAFFIQ, Sharon Casilda THEOPHILUS, Nujaimin UDIN, Mohamad Azhari OMAR, Mohamed Saufi AWANG, Abdul Rahman Izaini GHANI, Zamzuri IDRIS, Jafri Malin ABDULLAH

**Affiliations:** 1 KPJ Ipoh Specialist Hospital, Ipoh, Perak, Malaysia; 2 Department of Neurosciences, School of Medical Sciences, Universiti Sains Malaysia, Kubang Kerian, Kelantan, Malaysia; 3 Department of Neurosciences & Brain and Behaviour Cluster, Hospital Universiti Sains Malaysia, Universiti Sains Malaysia, Kubang Kerian, Kelantan, Malaysia; 4 Brain and Behaviour Cluster, School of Medical Sciences, Universiti Sains Malaysia, Kubang Kerian, Kelantan, Malaysia; 5 Division of Neurosurgery, Faculty of Medicine, Universiti Malaya, Kuala Lumpur, Malaysia; 6 Gleneagles Hospital Kuala Lumpur, Kuala Lumpur, Malaysia; 7 Department of Neurosurgery, Tunku Abdul Rahman Neuroscience Institute (IKTAR), Hospital Kuala Lumpur, Kuala Lumpur, Malaysia; 8 Division of Neurosurgery, Hospital Canselor Tuanku Muhriz Universiti Kebangsaan Malaysia, Kuala Lumpur, Malaysia; 9 Department of Neurosurgery, Sarawak General Hospital, Kuching, Sarawak, Malaysia; 10 Department of Neurosurgery, Hospital Queen Elizabeth II, Kota Kinabalu, Sabah, Malaysia; 11 Division of Neurosurgery, Hospital Tuanku Ja’afar, Seremban, Negeri Sembilan, Malaysia; 12 Department of Neurosurgery, Hospital Sungai Buloh, Sungai Buloh, Selangor, Malaysia; 13 Department of Neurosurgery, Penang General Hospital, Georgetown, Pulau Pinang, Malaysia; 14 Department of Neurosurgery, Hospital Sultanah Aminah Johor Bahru, Johor Bahru, Johor, Malaysia; 15 Department of Neurosurgery, Hospital Sultanah Nur Zahirah, Ministry of Health Malaysia, Kuala Terengganu, Terengganu, Malaysia; 16 Department of Neurosurgery, Hospital Raja Permaisuri Bainun, Ipoh, Perak, Malaysia; 17 Division of Neurosurgery, International Islamic University Malaysia (IIUM), Kuantan, Pahang, Malaysia

**Keywords:** Neurosurgical Association of Malaysia, neurosurgery, neurosciences, World Federation of Neurosurgical Societies

## Abstract

The history of neurosurgery in Malaysia traces back to 1962 and is filled with stories of vibrant and humble neurosurgeons who have dedicated their life to patients and professions. The early development of neurological and neurosurgical services begins from the establishment of the neurosurgery unit at Hospital Kuala Lumpur (HKL), followed by the foundation of the Tunku Abdul Rahman Neuroscience Institute (IKTAR). Due to the exponentially increased demand for the care of neurosurgical patients, many universities and government hospitals have opened their neurosurgical units. In 2001, the formal residency training programme (USM Masters in Neurosurgery) started and since then has produced qualified neurosurgeons that empowered and shaped the present generation. The formation of the Neurosurgical Association of Malaysia (NAM) is another turning point towards bidirectional collaboration with the World Federation of Neurosurgical Societies (WFNS). Many opportunities were created for educational activities and the expansion of subspecialties in neurosurgery. This article describes the impact of the past neurosurgeons and the endeavors that they had gone through; the present neurosurgeons who pioneered the current neurosurgical services in Malaysia, and the future neurosurgeons that will continue the legacy and bring neurosurgery further ahead in this country.

## Neurosurgery in Kuala Lumpur (The Early Days)

**Figure 1:**
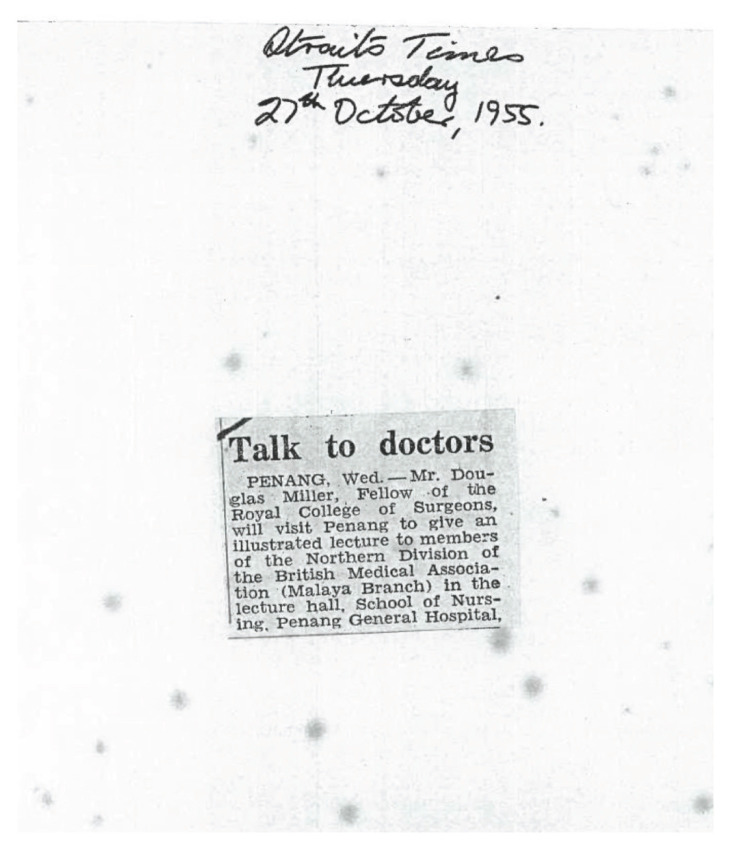
An announcement of official visit of Mr Douglas Miller to Penang General Hospital

**Figure 2:**
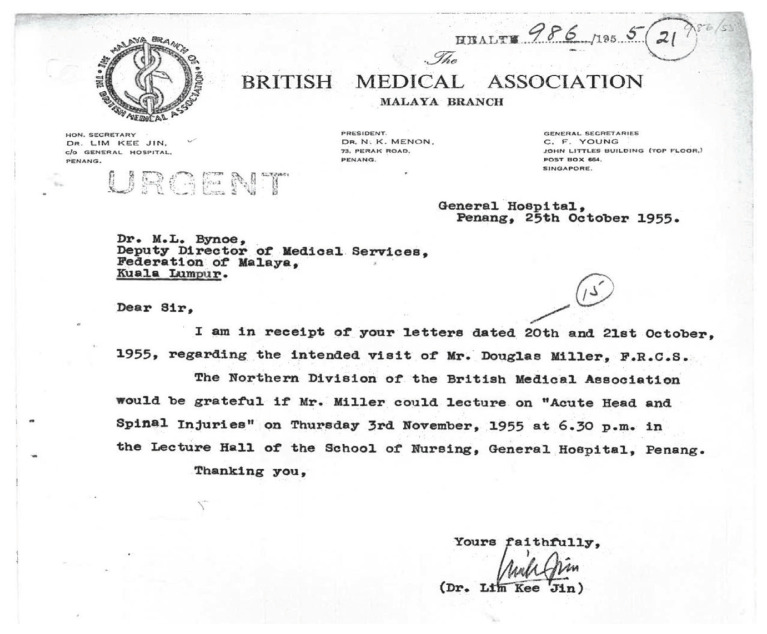
The first lecture presented by Mr Douglas Miller at Penang General Hospital

**Figure 3:**
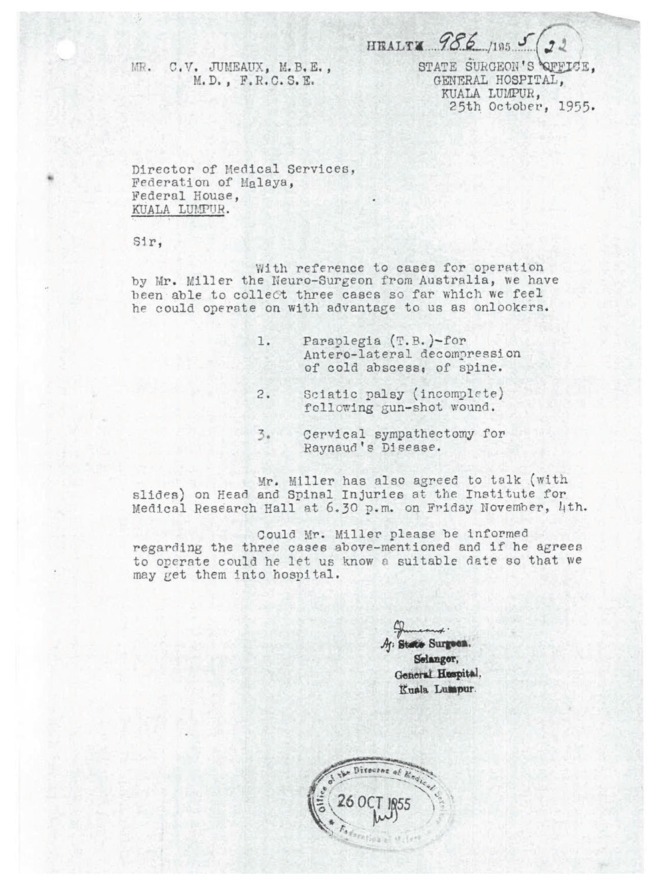
Three proposed operations during Mr Douglas Miller’s visit to GHKL

Neurosurgery was virtually unheard of in the 1950’s in the then Federation of Malaya. There was understandably excitement when Mr Douglas Miller FRCS, a neurosurgeon from Australia, made an official visit to Singapore and Federation of Malaya in 1955 under the Colombo Plan (Figure 1). He presented a lecture on ‘Acute head and spine injuries’ on 3 November 1955 at the School of Nursing, Penang General Hospital to members of Northern Division of the British Medical Association (Malaya branch) (Figure 2). There was also proposal for Mr Miller to operate on three cases viz:- i) paraplegia (tuberculous)-for antero-lateral decompression of cold abscess of the spine; ii) sciatic palsy (incomplete) following gun-shot wound; iii) cervical sympathectomy for Raynaud’s disease (Figure 3). On official records, this event marked the first ever Neurosurgical scientific presentation in Malaysia.

**Figure 4:**
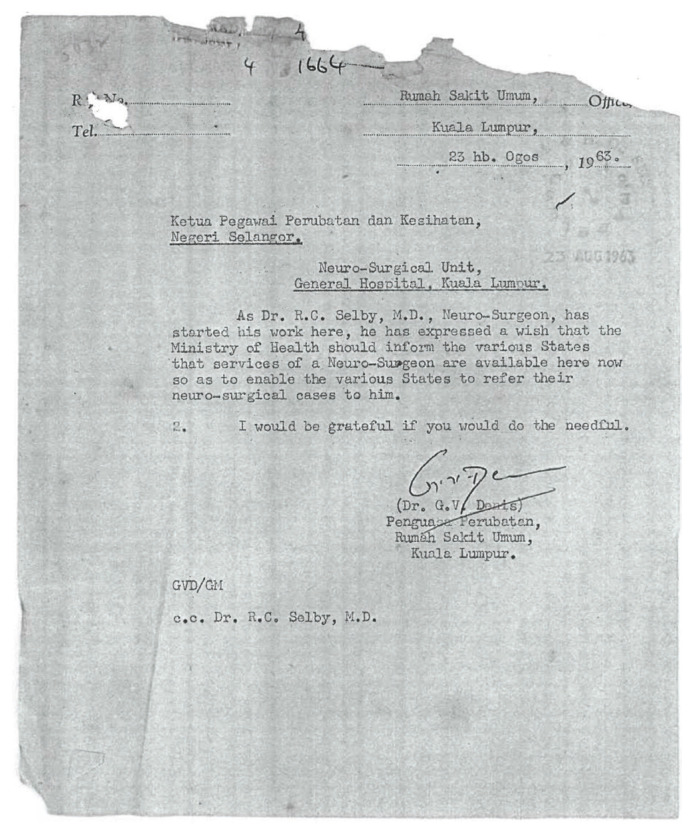
Official letter to announce the availability of neurosurgical service of Dr Roy Clifton Selby at GHKL

**Figure 5:**
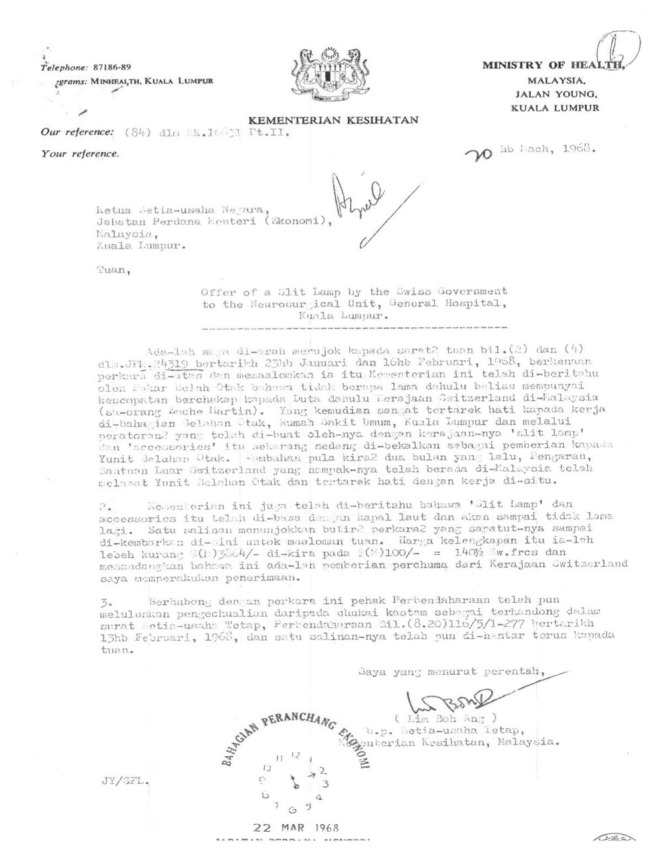
Letter of donation of slit lamp and accessories from the Switzerland government

**Figure 6:**
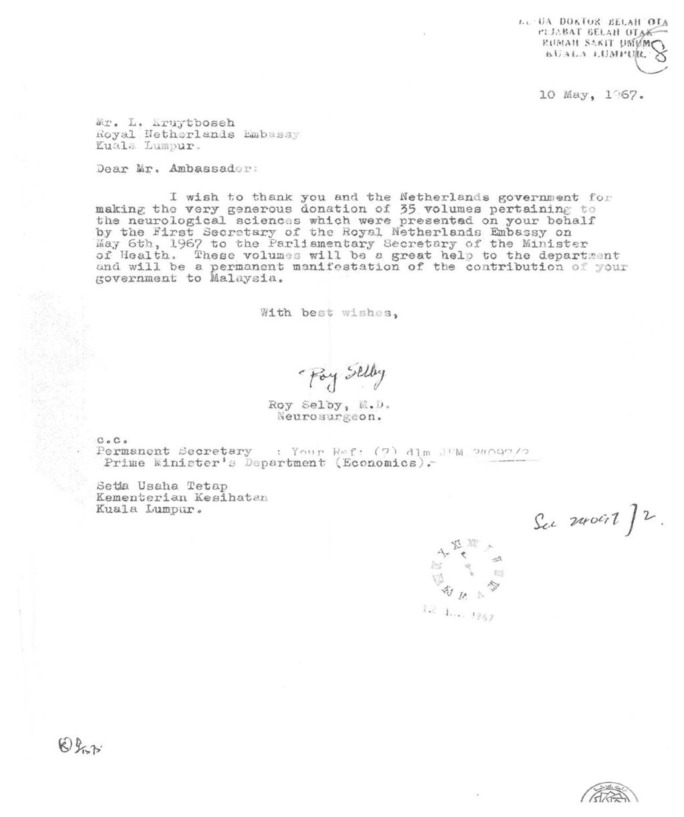
Letter from Dr Roy Clifton Selby thanking the Netherlands government for the generous donation received for the Neurological Unit in GHKL

Neurosurgery as a subspecialty of surgery actually began in Malaysia with the arrival of Dr Roy Clifton Selby under the United States of America (USA) CARE/MEDICO programme in 1962 (Figure 4). With the full support of Datuk Alhady, then the Head of Department of Surgery, Dr Roy Clifton Selby managed to set up a fully functional Neurosurgery Unit from scratch (a humble beginning at Ward 25) within a short period of two years in General Hospital Kuala Lumpur (GHKL, now known as Hospital Kuala Lumpur [HKL]). In the early phase, amongst the many challenges that Dr Roy Clifton Selby faced, apart from the clinical commitment, was to procure the necessary equipment for the operating room facilities. With the support from the Ministry of Health (MOH), he managed to get a donation from Switzerland and the Netherlands governments (Figures 5 and 6). His main mission was to recruit suitable doctors to be trained as neurosurgeons and neurologists so that they can continue the works he had started.

Of the many remarkable achievements of Dr Roy Clifton Selby, perhaps the most notable one, was that he managed to send five trainees to the USA for neurosurgical training annually for 5 years in the early phase. Of the five, only three opted to return to serve the motherland after completion of their training, respectively. The first to return was Dato’ Dr Nadasan Arumugasamy in 1969, followed by Dr Anavangot Mohandas in 1971 and Dr Swaran Singh Khera in 1972. Dr Sundaresan and Dr Lim Jit Kim did not return. Dr Roy Clifton Selby also undertook the overseas training of neurologist in the country. Of the three neurology trainees he sent overseas, only two returned to Malaysia: Dr C Balaratnam and Dr Sabri Rejab.

**Figure 7:**
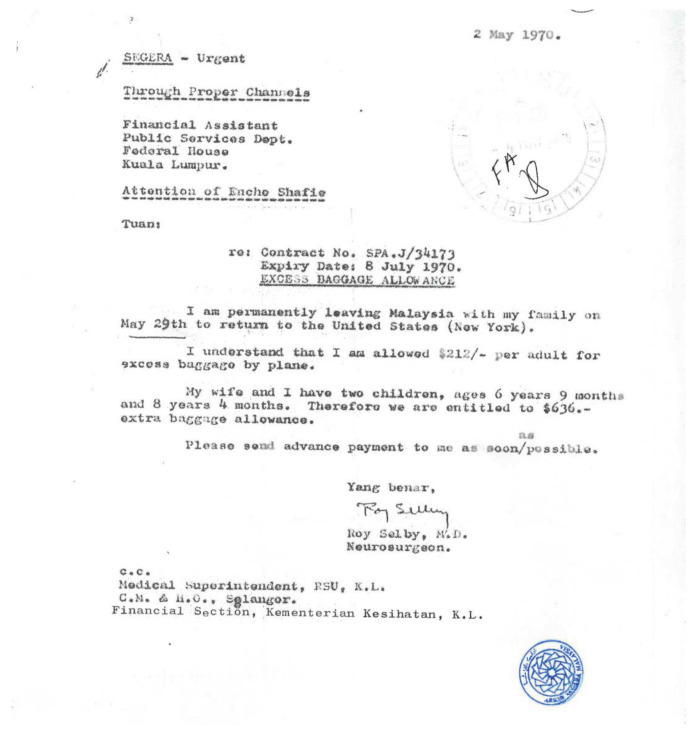
A letter from Dr Roy Clifton Selby confirming his permanent departure to return to the USA

**Figure 8:**
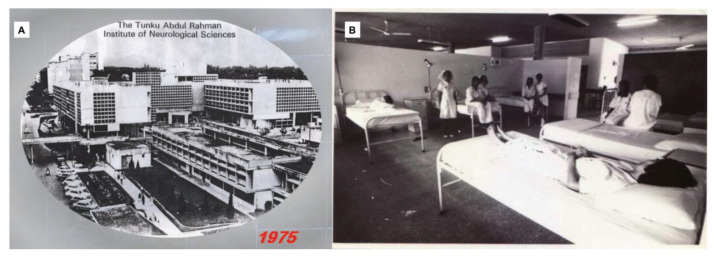
(A) The IKTAR at the early phase (1975), (B) Neurosurgery ward in early 1970s

Dr Roy Clifton Selby returned to USA in 1970 (Figure 7) and Dato’ Dr Nadasan Arumugasamy took over as the Head of Department of Neurosurgery, GHKL. The neurosurgical service in GHKL was boosted with the return of Dr Anavangot Mohandas in 1971. Dr Swaran Singh Khera returned in 1972 and was posted to Penang General Hospital, tasked with the responsibility of setting up neurosurgical service to cater for the northern region of the Malayan Peninsular. Neurosurgery in the early days was very challenging with the lack of facilities and all the patients scattered in different wards sprawled all over the large compound of GHKL. In 1970, the then Prime Minister of Malaya, Tunku Abdul Rahman Putra Alhaj laid the foundation stone for a separate building dedicated for neurosciences viz. neurosurgery, neurology and psychiatry. This building is now known as the Tunku Abdul Rahman Neuroscience Institute (IKTAR) (Figure 8).

In August 1973, the services of neurosurgery, neurology and psychiatry moved into IKTAR. IKTAR started with 240 beds, 80 beds for each specialty, respectively. The institute housed various services ranging from neurosurgery operation theatres (subsequently with intensive care unit ward), neuradiology (e.g. in those days, neurosurgeons themselves performed cerebral angiography by direct puncture of carotid artery/brachial artery), neuropathology, electroencephalography (EEG), electro-convulsive therapy and so on. By 1975, IKTAR was run by a small core of well-trained dedicated specialists and specialty skilled technicians and graduate nurses, many of whom had been trained overseas. In 1975 alone, more than 500 operations of the nervous system were carried out, in addition to over 2,000 contrast studies. Neuropathological examination of all surgical specimens were processed in the Institute and reported by Dato’ Dr Nadasan Arumugasamy, who also performed regular post-mortem brain cutting sessions.

IKTAR was officially opened by the then Minister of Health, YB Tan Sri Dr Lee Siok Yew in March 1975, after it had been operational for a year. A forum on neurological sciences in developing countries was held to commemorate the occasion. This forum was well attended by many foreign distinguished neurosurgeons which included Dr Paul C. Bucy and Dr A. Baker from USA; Dr G. Baumgartner and Dr Andre Haynal from Switzerland; Dr Bedbrook, Dr Cawte and Dr John Game from Australia; Dr Tsubaki from Japan, and Dr H. Wadia and Dr Ramamurthi from India. Dr Roy Clifton Selby also returned to Kuala Lumpur for this special occasion which was also attended by many regional specialists from ASEAN countries. In the prime days under the leadership of Dato’ Dr Nadasan Arumugasamy and Dr Anavangot Mohandas, IKTAR was highly regarded as a premier neurosurgery centre in the ASEAN region. However, in 1979, Dr Anavangot Mohandas decided to leave the government service and became the first neurosurgeon in private practice in Malaysia. About the same year, Dr Swaran Singh Khera emigrated to Adelaide, Australia. Dato’ Dr Nadasan Arumugasamy remained at the helm of neurosurgery service in the country until his retirement in 1986.

**Figure 9:**
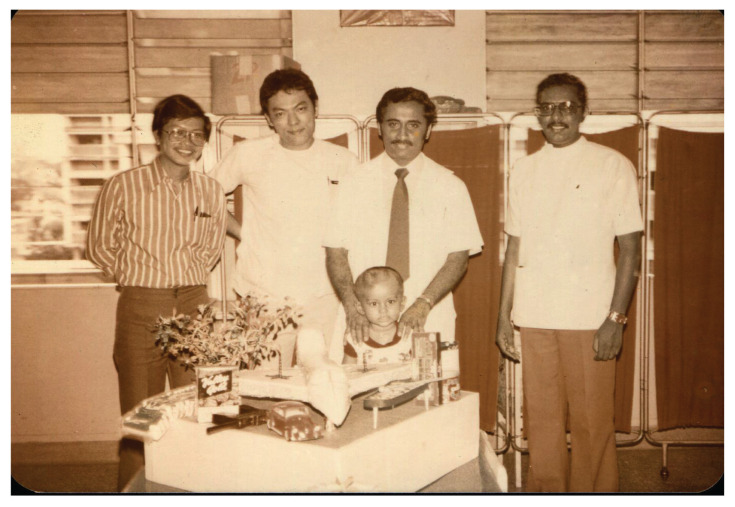
The photo was taken with a child being operated at GHKL. From left to right: Dr Tan Kok Joo, Dr Lim Meng Choo, Dr Anavangot Mohandas and Dr Gunasekaran Balasundaram

**Figure 10:**
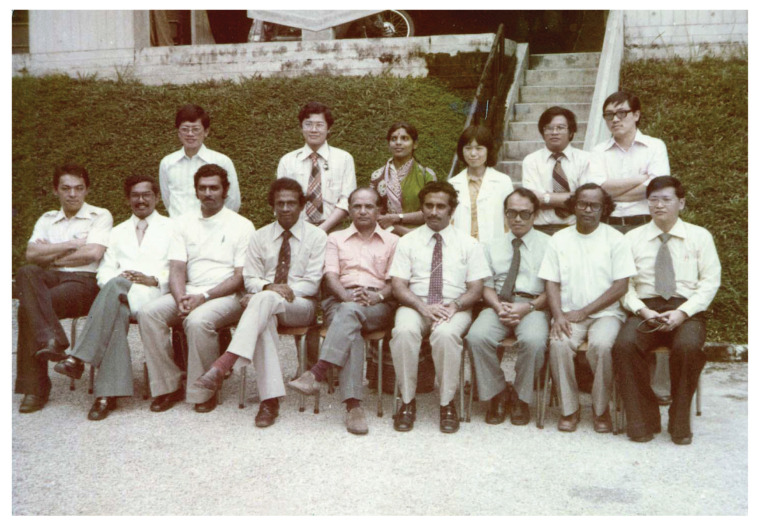
The photo was taken at GHKL during 1980’s. First row from left: Dr Lim Meng Choo, Dr Gunasekaran Balasundaram, Dr Nadasan Arumugasamy, Dr C. Balaratnam, Dr Anavangot Mohandas, Dr Sabri Rejab and others. Back row from left: Dr Tan Kok Joo, Dr Lee Foo Chang and others

Dato’ Dr Nadasan Arumugasamy undertook the training of more neurosurgeons to overcome the acute shortage of neurosurgeon following a lapse in neurosurgery training. Under a co-operative programme with the University of Adelaide, Dr Gunasekaran Balasundaram was sent there for training in 1980, followed by Dr Lee Foo Chang a year later. Dr Gunasekaran returned in 1984 whist Dr Lee returned in 1985, after successfully obtaining their Fellowship in Neurosurgery (FRACS) (Figures 9 and 10). Dr Gunasekaran, apparently frustrated by the government requirement to sit for bahasa Malaysia paper for confirmation as neurosurgeon, left for private practice in Gleneagles Hospital, Pulau Pinang in 1986. In 1988, Dr Lee Foo Chang also followed suit and left for private practice in Subang Jaya Medical Centre.

## Pioneers of Neurosurgery

### Dr Roy Clifton Selby

Dr Roy Clifton Selby was born on 28 September 1930 in Little Rock, Arkansas, USA. He received his MD cum laude from University of Arkansas Medical School. He underwent neurosurgery training at the Department of Neurology and Neurosurgery at Neuropsychiatry Institute at University of Illinois (1958–1961), followed by fellowship at the Department of Neurosurgery at Lahey Clinic in Boston, Massachusetts under the renowned name of Dr James Poppen (1961–1962).

Dr Roy Clifton Selby came to Kuala Lumpur, Malaysia in 1962 with his wife Marilyn, his one and a half-year-old son, his 1 month-old daughter and 2 dogs. He was responsible for setting up the groundwork for the development of neurosciences (neurosurgery in particular) in Malaysia. He came to Malaysia under the auspices of CARE MEDICO (an American non-profit government organisation). Dr Roy Clifton Selby will be remembered as the founder of neurosciences in Malaysia. He literally set up the Malaysian Neurosurgical Service from scratch. He undertook his pioneering works with enthusiasm, hard work and determination. Despite the lack of facilities and anaesthetist at his time, he performed about 10 surgeries per week. One of his trainees, Dr Swaran Singh Khera described him as ‘tall good looking man. He worked hard every day including weekends, appeared stern and serious when at work, but sort of shy and likeable at social gatherings’.

Dr Roy Clifton Selby was a strong advocate of International Medical Assistance to developing countries with his vision of arranging people to be trained in the USA and then return home to serve their motherland. He was instrumental in the recruitment of five neurosurgeons and three neurologists from Malaysia to be trained overseas notably in the USA and Canada, in addition to several paramedical staff in radiology, neuropathology and nursing. Dr Roy Clifton Selby also played an important role in the formation of Neurosurgical Foundation of Malaysia which led to the creation of a separate building for neurosciences viz. the IKTAR. For many years, the foundation has continued to provide direction and support for improving facilities in the related specialities, in addition to subsidising conferences and travel grants for doctors and nurses. In 1970, in recognition of his meritorious service to the country, Dr Roy Clifton Selby was conferred the federal royal award, ‘Commander of the Order of the Defender of the Realm’ by the King.

**Figure 11:**
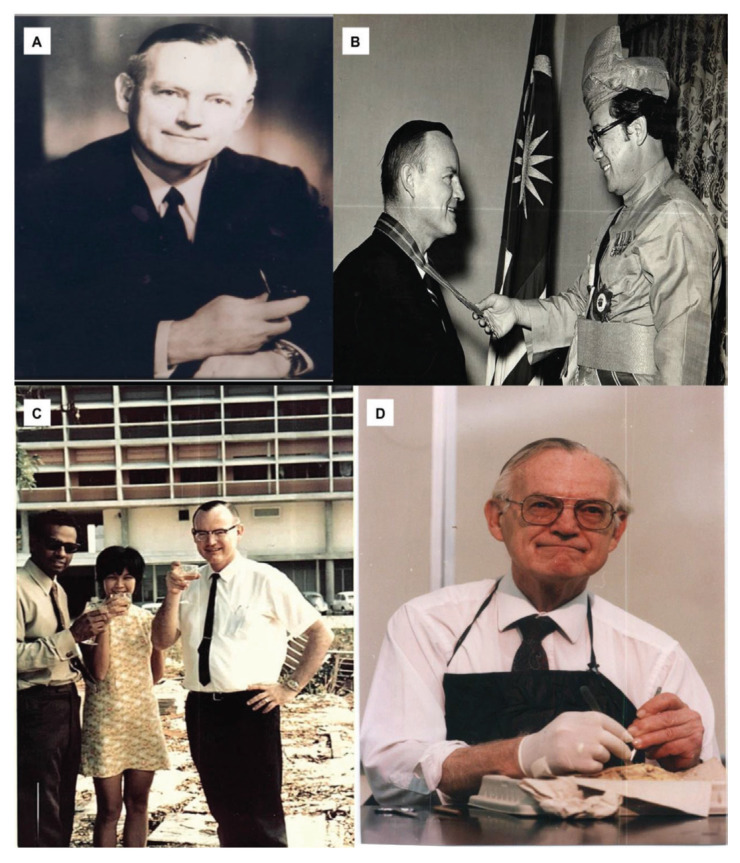
(A) Dr Roy Clifton Selby is the first neurosurgeon in Malaysia in 1960s, (B) Dr Roy Clifton Selby received Royal Award from the King in Malaysia a ‘Commander of the Order of the Defender of the Realm’, (C) Dr Roy Clifton Selby with Dr Nadasan Arumugasamy in 1970 in front of the IKTAR while under construction and (D) photo in 1996 after his cardiac transplant surgery

Dr Roy Clifton Selby returned to Chicago, USA in May 1970 after his first trainee, Dr Nadasan Arumugasamy completed his overseas training (Figure 11). He initially worked in Cook County Hospital as Chair of the Department of Neurology. He later ventured into private practice in his home city of Little Rock before retiring in 1986. Before his retirement, Dr Roy Clifton Selby was active in teaching and delivering lectures in many local universities. He was Adjunct Professor at East Texas State University teaching neuroendocrinology, psychology and biomedical ethics. He was a member of many national and international professional societies. He was Chairman of the Archives Committee of the American Association of Neurological Surgeons (AANS) and had conducted numerous video interviews of senior neurosurgeons and neuroscientists worldwide. He was also a pioneer in spine surgery, one of his most important contributions was his insistence on the spine to be an important part of neurosurgical practice. He was a founder of the Lumbar Spine Society.

**Figure 12:**
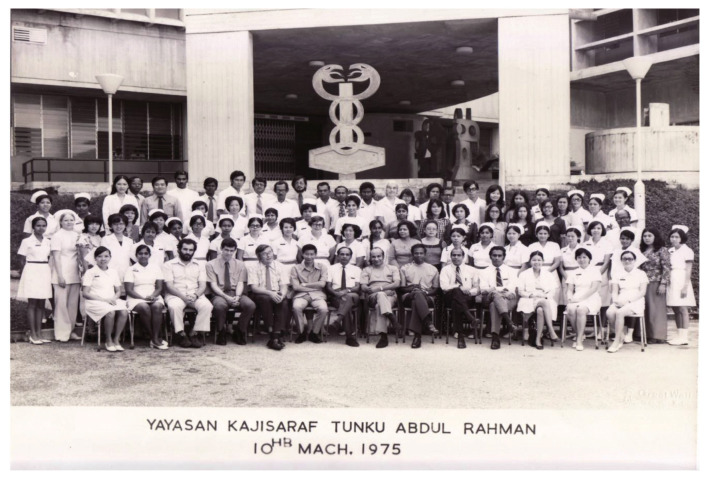
The opening of the IKTAR. Sitting fifth from the left front row is Dr Roy Clifton Selby. Other prominent figures include Dr Nadasan Arumugasamy, Dr Balaratnam and Dr Anavangot Mohandas

Dr Roy Clifton Selby was also a talented writer. Besides 18 published papers and editorials, he also wrote essays and short stories. One of his most popular essays was ‘A Delicate Operation’ which was published in Harper’s magazine in 1975 (1). He also wrote on his surgical experience in Malaysia which included two case reports of tropical fungal infection *Allescheria boydii* and cryptococcal granulomata (2, 3), a book chapter on Tropical Neurology (e.g. neurosurgical aspects of leprosy) (4, 5) and a study of intracranial neoplasm (6). He was also a passionate lover of classical music. He made a return trip to Malaysia in March 1975 for the official opening of the IKTAR (Figure 12) and the Symposium on Neurological Sciences in Developing Countries which was held in conjunction with the opening. In 1976, Dr Roy Clifton Selby had a heart attack which subsequently required a bypass surgery. In 1986, a pacemaker was inserted. In April 1995, he received a heart transplant from a 13-year-old girl. He put his old heart in a glass jar for display on his mantle because ‘it will make a good conversation piece’. In January 2002, he wrote:-

‘No city noise, no sirens; quietude. Eight miles west of my doctor and hospital (which I seek to avoid) and only twelve miles from the cemetery that should reduce expenses a bit’

Up until his last days of life, he still maintained his sombre sense of humour. Dr Roy Clifton Selby passed away on 20 January 2002 at the age of 71. He had left a legacy in neurosurgery which will be remembered and cherished forever by all of us.

‘As clinicians, perhaps we must remain advocates for the individual, resist every effort of planners to impose a system or limitations on us and the service we must provide and improve upon’ wrote Dr Selby.

This should serve as a constant reminder for all of us in our current environment with increasing corporatisation of health care (adapted from a memoriam written by Dato’ Dr Nadasan Arumugasamy, dated 7 April 2002 in an article ‘A humanist neurosurgeon: A legacy of Dr Roy Selby’ by Lichterman et al. (7).

### Dato’ Dr Nadasan Arumugasamy

Dato’ Dr Nadasan Arumugasamy was born on 15 January 1934 in Taiping, Perak. He received his early education in King Edward VII School in Taiping and later in St Michael Institution in Ipoh. He graduated as a doctor (MBBS) in 1960 from King Edward VII College of Medicine, University of Malaya, Singapore. While serving as a medical officer in GHKL, he was recruited by Dr Roy Clifton Selby as his very first neurosurgery trainee. Dato’ Aru (as he was usually referred to) was the first Malaysian neurosurgeon to be trained in the USA, in 1964. He started a one-year fellowship programme in Neurophysiology in the University of Wisconsin, followed by another year of Neuropathology Fellowship programme in Albert Einstien College of Medicine in New York before embarking on the Neurosurgery Residency programme in Northwestern University Medical School, Chicago from 1966–1968. In 1969, he became the first Malaysian Neurosurgeon to be certified by the American Board of Neurological Surgery.

**Figure 13:**
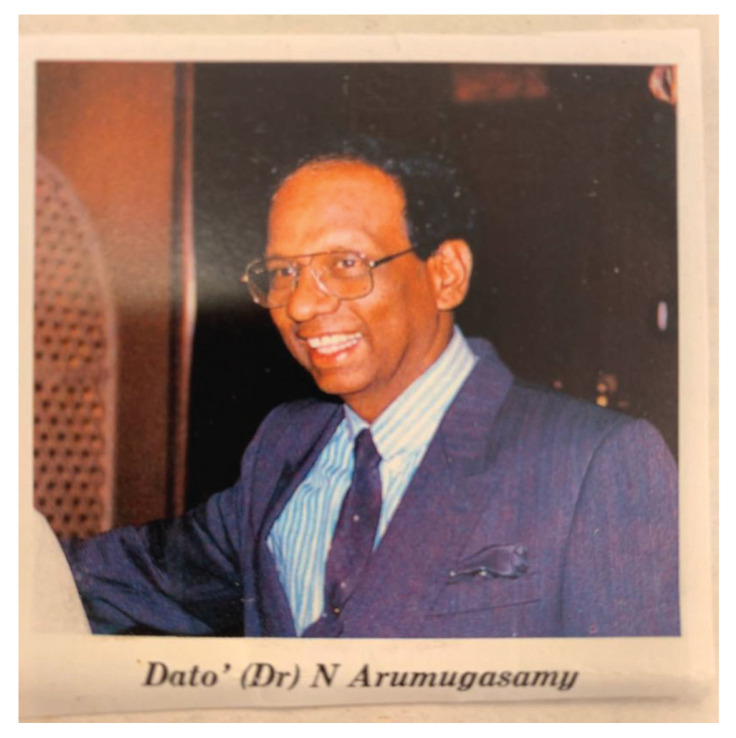
Dato’ Dr Nadasan Arumugasamy is the the first Malaysian neurosurgeon to be trained in the USA and the first Malaysian neurosurgeon, taking over Dr Roy Clifton Selby in 1969

Dato’ Aru returned to Malaysia at the end of 1969 and took over from Dr Roy Clifton Selby as the head of the Neurosurgery Department in HKL. In his heydays, besides his heavy clinical and administrative responsibilities, Dato’ Aru practically had to do everything himself which included neuro-radiological procedures (e.g. cerebaral angiogram by direct puncture of the carotid or brachial arteries), neuropathology laboratory service (e.g. he read and reported on the all histological specimens and conducted brain cutting sessions) and neuro-physiology investigations (e.g. reporting on EEG). In essence, Dato’ Aru was a neurosurgeon, neurologist, neuroradiologist, neurophysiologist and neuropathologist all moulded into one. He was truly a complete neurosurgeon (Figure 13). Dato’ Aru was instrumental in establishing the Neurosurgical Foundation of Malaysia and erecting a new building – the IKTAR which provided the specialised services of neurosurgery, neurology and psychiatry confined to one premise. The institute was highly regarded as a landmark of achievement in the development of neurosciences in the ASEAN region at that time. Dato’ Aru was the Founding Director of the Neurosurgical Foundation and remained active as the Honorary Secretary General until the very last days of his life.

Since 1958, Dato’ Aru had published and presented more than 61 papers on various subjects of the central and peripheral nervous system, including his favourite topic on cryptococcal infection/cryptococcoma (8) and tumours like cerebellar sarcoma and meningiosarcoma (9). Dato’ Aru was fellow and member and council member of countless national and international professional societies and non-governmental organisations (NGO). In 1995, he became the prestigious Fellow of Academy of Sciences Malaysia and he was unanimously elected as the first President of the Neurosurgical Association of Malaysia (NAM) which was founded in 2001.

In recognition of his invaluable contributions to the country, Dato’ Aru was conferred the royal award of Darjah Sultan Ismail Johore (DSIJ) which carries the title Dato’. He also received another award of Johan Setia Mahkota (JSM) in 1980. After his optional retirement in 1986, Dato’ Aru remained active as visiting consultant to a few established private hospitals, allowing him more time to indulge in his favourite sport of golf. Dato’ Aru passed away after a short spell of illness on 19 December 2003 at the age of 69. He has blazed the trail for neurosurgery in this country and has left a legacy we Malaysians are forever grateful.

### Dr Anavangot Mohandas

Dr Anavangot Mohandas was the second Malaysian neurosurgeon to be trained in USA. He was born on 28 August 1937 in Sungkai, Perak. He was the fourth of six children of an estate doctor. After the second World War, he was sent back to India to live with his grandparents. He had his early education in South India. He graduated as a doctor from Vitkal Unversity, achieving his childhood ambition to be a doctor like his father. He returned to Malaysia in 1963 and worked as medical officer in hospitals in Taiping, Kampar and Tapah before ended up as neurosurgery trainee under Dr Roy Clifton Selby in GHKL.

He left for the USA in 1966 and completed his residency programme in the University of Minnesota. Dr Anavangot Mohandas was a pioneer in the study of brain death. During his training in Minnesota, his paper titled ‘Brain death – a clinical and pathology study’ was published in a textbook on Coma by Plum & Posner in the USA (10, 11). Dr Anavangot Mohandas returned to HKL in 1971 and worked closely with Dato’ Dr Nadasan Arumugasamy. He was very friendly and approachable, described by his contemporaries as honest, forthright, a competent and stylish neurosurgeon and above all very charitable and just. He was a kind and caring person, well respected and admired by his colleagues and juniors alike. Dr Anavangot Mohandas left the government service and became the first neurosurgeon to venture into private practice in Malaysia. Although busy with his own private practice, he was ever ready to render assistance and advice to younger neurosurgeons. While I was working in Ipoh Specialist Hospital, I sought his help once and he drove all the way from Kuala Lumpur to assist me in clipping a complex anterior communicating artery aneurysm. He was gracious and did not bother to charge the patient at all.

**Figure 14:**
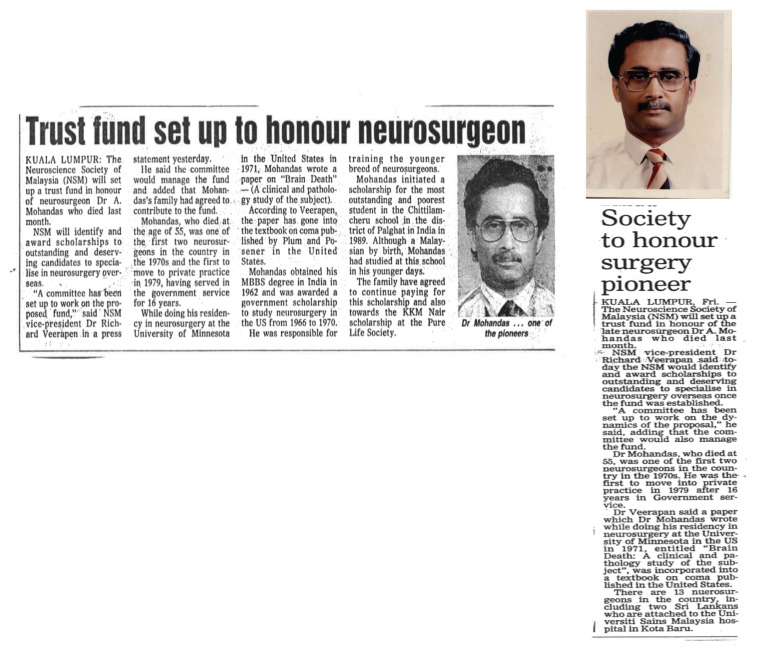
In recognition of the late Dr Anavangot Mohandas contribution to neurosurgery in Malaysia, the Mohandas Educational Trust Fund was launched

It came as a shock to all of us when Dr Anavangot Mohandas suddenly succumbed to a myocardial infarct after doing his evening ward round in 31 January 1993. He was only 55 year old. The sudden demise of Dr Anavangot Mohandas was a great loss to the nation. The neuroscience community, particularly the neurosurgery fraternity had lost a great friend and a trusted colleague. In recognition of his invaluable contribution to neurosurgery, the Neuroscience Society of Malaysia (NSM) in 1994 had set up a trust fund with the objectives to promote co-operation, research and publication in the field of neurosciences, particularly in neurosurgery, a ‘Mohandas Memorial Lecture’ will be delivered by a distinguished personality annually in conjunction with the scientific meeting of the Neuroscience Society of Malaysia (Figure 14). Dr Anavangot Mohandas always carried a little book with these sayings which he lived by them:

‘My work is my prayer,

Never live on regrets and debts,

Duty is never sweet’

### Dr Swaran Singh Khera

Dr Swaran Singh Khera was born in 1936 in Tanjung Malim, Perak, a son of a migrant from Punjab who came to Peninsular Malaya in 1915. He is the middle sibling of five, four boys and one girl. He attended the first English school in Tanjung Malim established by Rev Rajakarier, an English master from Ceylon (Sri Lanka) in 1947. He was the first student from that school to get Grade One in Cambridge School Certificate and subsequently did his Higher School Certificate in Anglo Chinese School, Ipoh. He graduated from Royal College of Surgeons Dublin in 1963 and won the first prize Gold Medal in Obstetrics and Gynaecology. Upon his return in July 1963, he was posted to Penang General Hospital for his internship in General Surgery under Dr Vanniasingham who subsequently recommended him to Dr Roy Clifton Selby to do neurosurgery in 1966.

**Figure 15:**
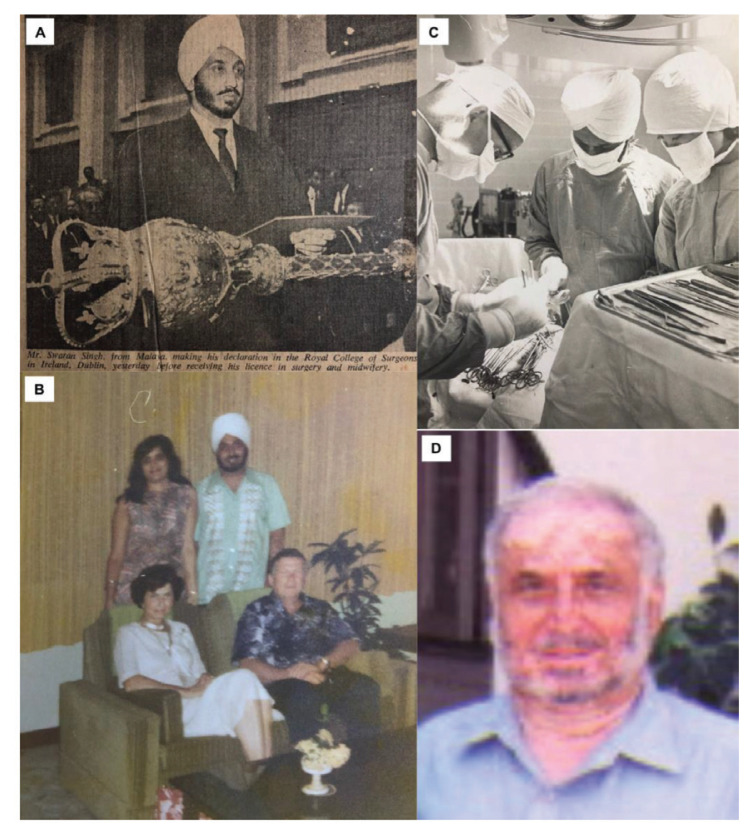
(A) Dr Swaran Singh Khera graduated from Royal College of Surgeons in Dublin in 1963, (B) Dr Swaran Singh Khera and his wife with Professor Dr Charles Drake and his wife in Canada, (C) A photo of him assisting Dr Roy Clifton Selby during surgery in 1967 and (D) Dr Swaran Singh Khera at the age of 84

In 1967, he was sent to Canada to be trained under the world famous neurosurgeon Dr Sir Charles Drake in University of Western Ontario (Figure 15). He returned to Malaysia in 1972. He was the third Malaysian neurosurgeon to have returned from overseas training. He was given the responsibility to set up Neurosurgical service in the northern region of Peninsular Malaya as the first neurosurgeon in Penang General Hospital. The lack of proper equipment at that time compelled him to improvise surgical instrument from the local hardware shop. He resigned from government service after seven years and emigrated to Adelaide, Australia in 1979. At the age of 84, Dr Swaran Singh Khera is currently happily and physically active with three sons, a grandson and a great grandson.

### Dr Narayan Sundaseran

**Figure 16:**
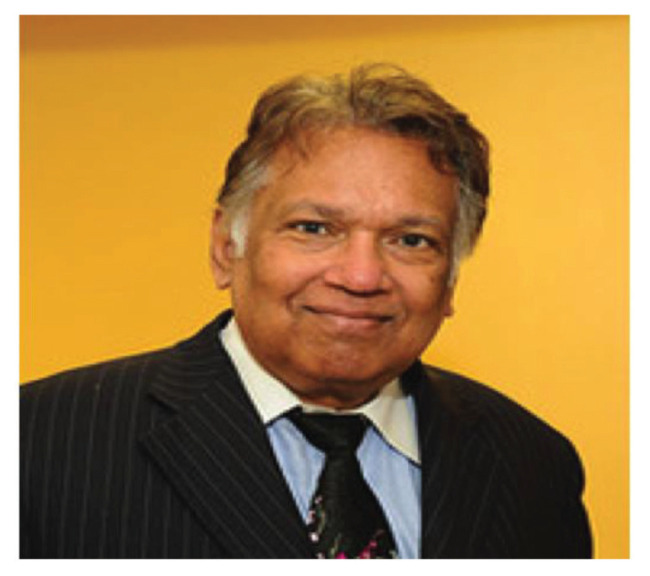
Dr Narayan Sundaresan is currently a Clinical Professor of Neurosurgery at Mount Sinai Medical Center, New York

Dr Narayan Sundaresan was the fifth neurosurgery trainee to be sent overseas. He was supposedly designated to serve East Malaysia after completion of his training. He was trained in the Northwestern University, Illinois under Dr Paul Bucy. He decided to stay back and work in Memorial Sloan Kettering Cancer Center after completing his residency training. At one stage, he planned to come back to Malaysia to establish a private centre in 1995 but the project failed. Currently, Dr Narayan Sundaresan is Clinical Professor of Neurosurgery at Mount Sinai Medical Center, New York and Chief of Neurosurgery at BronxCare Health Center, New York (Figure 16). He also practice at Northwell Lenox Hill Hospital in Manhattan.

## The University Hospital

### Neurosurgery in Universiti Malaya

**Figure 17:**
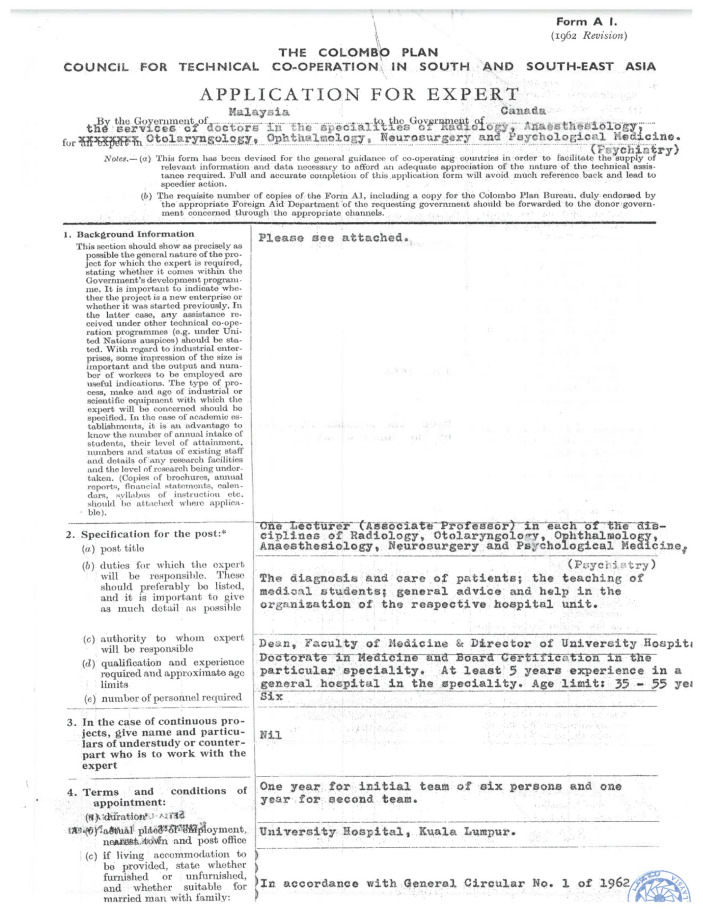
A request for more specialists under the Colombo Plan 1962

Back in 27 September 1965, the Founding Dean of the Faculty of Medicine, Universiti Malaya (UM), Professor TJ Danaraj, had applied to the Government to obtain the services of lecturers under the Colombo Plan in each of the following disciplines of Radiology, Otolaryngology, Ophthalmology, Anaesthesiology, Neurosurgery and Psychological Medicine (Figure 17). This request was formally tabled in Dewan Rakyat. It was only in 1970’s, that the then University Hospital (UH), (now the University of Malaya Medical Centre [UMMC]) under the Faculty of Medicine managed to procure the services of Dr Jagdish C Chawla, a neurosurgeon from the University of Bristol, United Kingdom (UK). During his affiliation with UH, Dr Jagdish C Chawla managed to recruit patients with dementia and communicating hydrocephalus for intracranial pressure (ICP) study. His paper titled ‘Intracranial pressure in patients with dementia and communicating hydrocephalus’ was published in *Journal of Neurosurgery* (12). Dr Jagdish C Chawla stayed on with the UH for a few years and returned back to the UK. Dr Jagdish C Chawla was fondly remembered by Professor Saw Huat Seong, a pioneer heart surgeon and the first batch of medical graduates from University of Malaya in 1968, as a very caring and fatherly neurosurgeon who advocated treating patients in a holistic manner with patient-centered mindset. Towards the end of career, Dr Jagdish C Chawla was a consultant and honorary lecturer at the Welsh Spinal Injuries and Neurological Rehabilitation Unit, Rookwood Hospital, Cardiff. He passed away in January 2013 at the age of 79 years.

**Figure 18:**
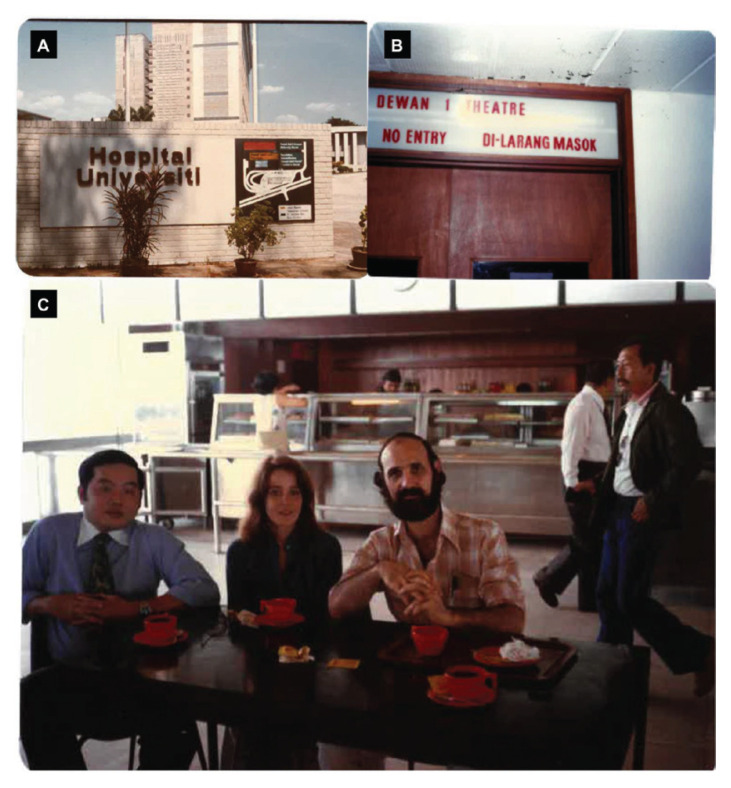
University of Malaya in the early days. (A) The University Hospital entrance, (B) the main entrance to the theatre room in 1978 and (C) Dr Richard L Rapport and his wife, Mrs Valerie Trueblood in 1979, accompanied by Dr Ernest Yeoh (general surgeon)

Neurosurgical services at the UH saw an abeyance until the arrival of two American neurosurgeons, Dr Richard L Rapport from Seattle (Figure 18) and Robert Dunn from St Louis, in 1978. Their sojourn in Malaysia was by virtue of a grant to the Dean of the School of Medicine of University of Washington, courtesy of the China Medical Board of New York (part of the Rockefeller Foundation). Besides providing the clinical services so desperately wanting in the hospital, they also undertook the Basic Neurosurgical Training for aspiring young surgeons in the Department of Surgery. They inspired two young promising surgeons to embrace and further their neurosurgery training overseas. These competent talents namely Dr Fadzli Cheah Abdullah left for Glasgow, Scotland in 1980 to be trained under Professors William Bryan Jennett and Graham Teasdale. Dr Chee Chee Pin followed in suit in 1984. He initially trained in Glasgow and later with Dr Derek Gordon in Belfast.

**Figure 19:**
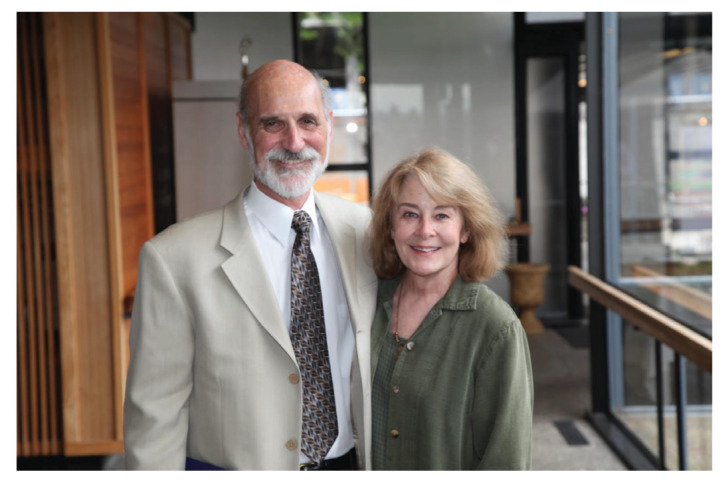
Dr Richard L Rapport and Mrs Valerie Trueblood. He currently resides in Seattle and is a clinical professor at the University of Washington, School of Medicine in the Department of Neurological Surgery

Amongst the many neurosurgical procedures carried out by the two neurosurgeons, Dr Richard L Rapport also successfully performed awake surgery in 1979 on three patients with meningioma, glioma and AVM, respectively. Dr Richard L Rapport was particularly interested in language function in bilingual/multilingual Chinese patients. Based on his experience in Malaysia, he published a paper on ‘Language function and dysfunction among Chinese- and English-speaking polyglots: Cortical stimulation, Wada testing and clinical studies’ in the *Journal Brain and Language* (13). Both of them returned to the USA at the end of 1979. Dr Richard L Rapport did come back again to UH during his sabbatical, from February to May in 1990. He currently resides in Seattle and is a Clinical Professor at the University of Washington School of Medicine in the Department of Neurological Surgery (Figure 19).

**Figure 20:**
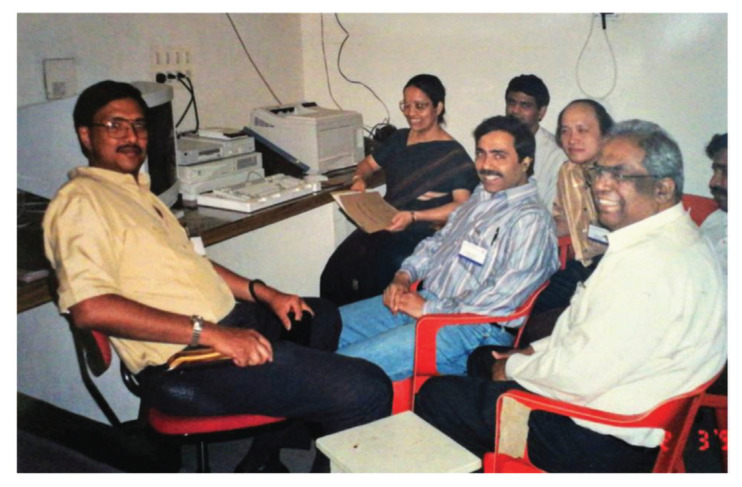
Photo of Dr Richard Veerapan (left) and Dr Chee Chee Pin (third from right) in 1987

The neurosurgical service in UH was once again disrupted with departure of Dr Robert Dunn and Dr Richard L Rapport in 1979. After a three-year hiatus, the services resumed with the arrival of Dr Jeffrey Coltheart in 1982. Dr Coltheart, from Tasmania Australia, was a neurosurgery registrar senior to Dr Fadzli Cheah Abdullah during their training days at the Institute of Neurosciences, Southern General Hospital, Glasgow during the 1980–1982 period. Dr Jeffrey Coltheart left Malaysia in 1985. Dr Chee Chee Pin returned in 1986 after completing his training in Belfast, Northern Ireland. He was at about the same time joined by Dr Richard Veerapan, who had completed his neurosurgery training in Frenchay Hospital, Bristol and returned the same year (Figure 20). In 1986, Dr Coltheart and Dr Chee Chee Pin published a case series of ‘Cerebritis preceding cerebral abscess formation: A report of three cases’ in the *Australian Journal of Surgery* (14). After a few years at UH, Dr Richard Veerapan left for private practice in 1988, followed by Dr Chee Chee Pin in 1991. The neurosurgical services after the departure of Dr Chee Chee Pin saw an era of crests and troughs, spanning the rest of the decade of the 1990s. Gaps were filled at different intervals by Dr Lim Heng Tien, Dr Nachiappen, Dr Liew Shih Shing (a Malaysian lady Doctor who trained in the USA) and intermittently by Dr Ahmad Zubaidi Abdul Latiff who was covering from HKL on the good graces of the MOH. This was a protracted period. Despite the tireless contributions of the above who were synonymous with tenacity and resilience, sadly the services repeatedly caved. The limitations of support from the MOH, the university administrative structure, the challenges of funding, resources and human capital (at the turn of the millennium there were only about 20 neurosurgeons serving a population of 23.2 million) led to unsustainability. The establishment of a Neurosurgical Unit to provide a sustainable comprehensive practice, potentially evolving to a stand-alone Tertiary Centre remained elusive.

Dr Vicknes Waran is an esteemed undergraduate alumnus of the Faculty of Medicine of UM who started his training in neurosurgery at IKTAR, HKL after obtaining his Fellowship of the Royal Colleges of Surgeons (FRCS) in Surgery in General, with a Special mention. He went on for further training in neurosurgery at Toranomon Hospital in Tokyo, Japan and then onto a national training post in neurosurgery at Addenbrooke’s Hospital, Cambridge, UK where he completed his training and obtained the FRCS (Neurosurgery). After honouring his commitments to the MOH at HKL, Dr Vicknes Waran responded to the call of establishing an Academic Unit of Neurosurgery in UM, providing comprehensive neurosurgical services.

The period of 2001 to mid-2003 saw the establishment and exponential development in the provision of neurosurgical services in UM and in the Klang Valley. Dr Vicknes Waran was aided by Dr S Sukumar for service provision. Between May and August 2002, the first three trainees were recruited. Two were also UM undergraduates, namely Dr Dharmendra Ganesan who had also obtained his FRCS in Surgery in General earlier and Dr Kalai Arasu Muthusamy, both of whom had graduated from UM’s own Master of Surgery Programme in May 2002. The third, Dr Hari Chandran started with the division of neurosurgery as its first service provision medical officer. He was previously from the MOH at HKL and had completed surgical training and obtained the FRCS in Surgery in General, after an 18-months stint with the General Surgery Division of UM, while providing neurosurgery service by being on the neurosurgery on-call rota. These three trainees, were not only responsible for clinical management but were also mentored in academia, research, publication, supervision, administration and management. Their in-house training was subsequently recognised for entry into the final stages of the comprehensive training programme in the UK and Ireland. Dr Dharmendra Ganesan and Dr Hari Chandran went on to get their FRCS (Neurosurgery) by completing their training at Addenbrookes Hospital, Cambridge and John Radcliffe Hospital, Oxford, respectively. Dr Kalai Arasu Muthusamy completed his PhD in the University of Oxford. The successful return of these three candidates heralded the declaration of UM as a fully-fledged training centre. In tandem with the Masters of Neurosurgery Programme established in the Universiti Sains Malaysia (USM) by Dr Jafri Malin Abdullah, Dr Vicknes Waran was acknowledged as having established a parallel training pathway for neurosurgical trainees in UM. Aspirants with a postgraduate qualification in General Surgery, either with Master of Surgery or FRCS (Surgery in General) were enrolled into a Twinning Programme that entailed 3 years of training in UM, followed by a 2 to 3-year stint in an accredited training post in the UK or Ireland, culminating in the FRCS (Neurosurgery). The peer acknowledgement and mutual respect between these two stalwarts has indeed driven neurosurgery to the forefront in the region.

Dr Jagdeep Singh Nanra joined UM as a consultant neurosurgeon in October 2004 after training in Beaumont Hospital, Dublin and obtaining his FRCS (Neurosurgery). This was a welcome end to Dr Vicknes Waran’s 3-year tenure as the sole consultant neurosurgeon on-call daily for more than 3 years. With that the foundations were laid for the eventual establishment of a fully in-house Neurosurgery Training Programme in UM. The three initial candidates were succeeded by a series of a further seven trainees who went on to either complete their FRCS (Neurosurgery) or PhD. Drs Devaraj Pancharatnam and Amritpal Singh Sidhu went to Oxford University Hospitals and completed their fellowship in Skull Base Neurosurgery.

The rest of the UM Neurosurgery Training Alumni consists of:

- Dr S Sukumar a/l C Sivasubramaniam, FRCS (2001–2007), Consultant Neurosurgeon, Pantai Hospital, Kuala Lumpur
- Dr Sia Sheau Fung, PhD (2008–2019), Consultant Neurosurgeon, Thomson Hospital Kota Damansara, Selangor
- Dr Vairavan Narayanan, FRCS (Neurosurgery), current Senior Consultant Neurosurgeon and Head of Department, University of Malaya, Kuala Lumpur
- Dr Faizal Ahmad Bahuri, DPhil (Oxon), Consultant Neurosurgeon, University of Malaya, Kuala Lumpur
- Dr Devaraj Pancharatnam, FRCS (Neurosurgery) (2010–2017), Consultant Neurosurgeon, Gleneagles Hospital Medini, Johor
- Dr Kevin Sek Weng Yew, FRCS (Neurosurgery) (2010–2017), Consultant Neurosurgeon, Pantai Hospital, Kuala Lumpur
- Dr Amritpal Singh Sidhu FRCS (Neurosurgery), current Consultant Neurosurgeon, University of Malaya Medical Centre, Kuala Lumpur
- Dr Ravindran Karuppiah (2012), current Consultant Neurosurgeon, University of Malaya Medical Centre, Kuala Lumpur

As the divisional faculty increased, the path was paved for UM to launch its Master of Neurosurgery Programme. An arduous period of formulation, negotiations and robust discussions finally met with approvals from the UM Senate and acceptance by all the cohorts of the Neurosurgical Fraternity of Malaysia. The first intake was in November 2019 (disruptions in the intake schedules and timelines due to the COVID-19 pandemic). From Dr Vicknes Waran’s sole endeavour to its current state of appraisal as a major Neurosurgical Service and Training Centre in Malaysia, UM has progressively evolved in all facets of the neurosurgical compass:

### Subspecialisation

Provides general neurosurgical services and subspecialisation in:

- Skull base surgery
- Paediatric neurosurgery
- Neurosurgical oncology
- Stereotactic radiosurgery
- Vascular neurosurgery
- Epilepsy surgery
- Functional neurosurgery
- Complex spinal neurosurgery
- Interventional pain management

### Centre for Image Guided and Minimally Invasive Therapy (CIGMIT) in Neurological Surgery

This centre is a private public partnership (PPP) project that provides state-of-art treatment facilities for the neurosurgical patients at the UMMC and University of Malaya Specialist Centre (UMSC). The original plan was presented to the National Economic Planning (NEP) Unit for their input and subsequently received cabinet approval before implementation. The planning for this facility began in 2007 and the facilities opened to public in 2016.

The aim of developing this cutting edge centre was to ensure that the neurosurgeons who trained in UM would continue to remain and serve the university and the nation. The centre was imperative so our Malaysian patients could enjoy the benefits for such technology which at that time was only available in certain first world nations. The centre also served as the conterminous engine to drive the Unit ever to the fore. The centre was not only an innovative first for the nation in terms of equipment, it was also an innovative private-public partnership to fund a RM-upper-8 figure project. The funding mechanism developed was not just new to Malaysia but also somewhat new in a global context.

The facility consists of an operating room connected to a 1.5T magnetic resonance imaging (MRI) scanner, a second operating room connected to a CT scanner, and a Radiation Suite with a linear accelerator that allows complex brain tumours and other central nervous system (CNS) pathology to be treated non-invasively, in addition to being able to deliver conventional radiation treatment. To date this remains the only such centre in Malaysia. With the availability of this centre, theoretically patients should no longer require repeat operations for either misplacement of spinal fixations or brain tumours that have been not been satisfactorily treated. This centre has also proven to be financially profitable to the various stakeholders vested in it. It has become a reference site not just for the available equipment but also for its innovative business plan. All the neurosurgical colleagues within the Unit have been robustly involved in developing neurosurgery in general and their subspecialty niches and hobby horses, via innovations, collaborations, research and development notwithstanding busy practices and arduous commitments. UM has always and continues to be a proponent of continuing neurosurgical education. Working on its own, and with the NAM, the division has organised and conducted numerous neurosurgical workshops, education courses, meetings, symposiums and conferences on national, regional and international platforms.

**Figure 21:**
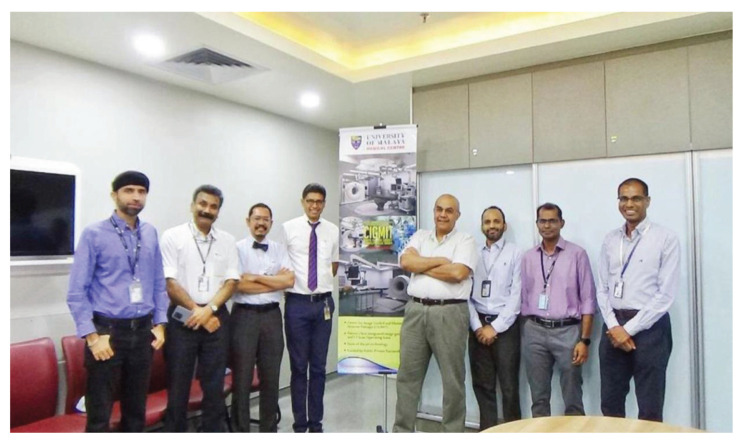
The current neurosurgeons working at the UMMC. From left to right are Dr Amritpal Sidhu, Dr Hari Chandran Thambinayagam, Dr Nor Faizal Ahmad Bahuri, Dr Dharmendra Ganesan, Dr Vikneswaran Mathaneswaran, Dr Vairavan Narayanan, Dr Ravindran a/l Karuppiah and Dr Kamal Azrin Abdullah @ Kalai Arasu Muthusamy

The division of neurosurgery was approached by the Joint Surgical Colleges Fellowship Examinations (JSCFE) Committee of the UK and Ireland to consider hosting and conducting the examinations in neurosurgery. UMMC successfully conducted and provided examiners for the FRCS (Neurosurgery) examinations in Kuala Lumpur at the UMMC in January 2019 and January 2020, which saw the enrolment of about 60 candidates on each occasion. Dr Vicknes Waran became the Medical Director of the UMMC. The headship of the division of neurosurgery was taken over first by Dr Dharmendra Ganesan from 2015 to 2018 and then Dr Kalai Arasu Muthusamy from 2018 to 2021. Both have steered the Unit to further peaks. Dr Hari Chandran succeeded Dr Kalai Arasu Muthusamy as the Head of the Division of Neurosurgery and continues to endeavour to follow in the tracks of his predecessors towards the advancement and betterment of the Unit. There are other seven neurosurgeons providing dedicated service in the Unit (Figure 21).

### Neurosurgery in Universiti Kebangsaan Malaysia

**Figure 22:**
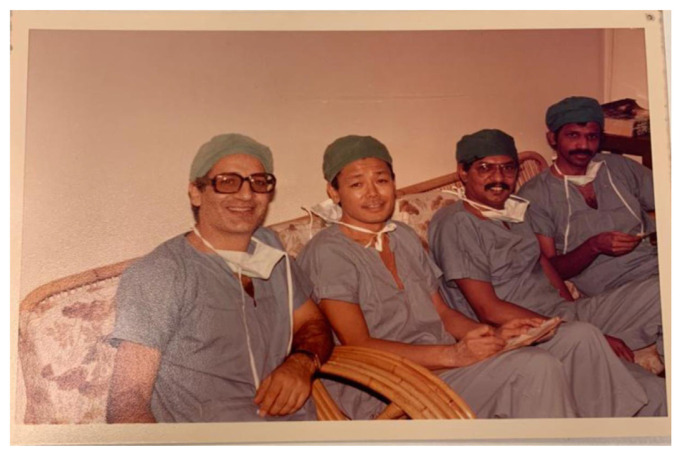
The neurosurgeons in scrubs during 1980s. From left to right: Dr Kazem Djavadkhani, Dr Fukushima from Japan, Dr Gunasekaran Balasundaram and Dato’ Dr Selvapragasam Thambiah

In 1982, Dr Kazem Djavadkhani, an Iranian neurosurgeon trained in Leuven, Belgium was recruited by Universiti Kebangsaan Malaysia (UKM) and initially joined Dato’ Dr Nadasan Arumugasamy in his department (Figure 22). With the arrival of Dr Fadzli Cheah Abdullah in 1983, a Unit of Neurosurgery was set up under the Department of Surgery, Faculty of Medicine, UKM. Dr Fadzli Cheah Abdullah graduated from UM in 1976, obtained his FRCS from Edinburgh in 1980 and left for Glasgow, Scotland in December 1980 for neurosurgery training in the Institute of Neurosciences under Professor Bryan Jennette and Professor Sir Graham Teasdale. The UKM Unit was initially housed within IKTAR and functioned very closely with the department of neurosurgery, although administratively independent.

The Unit was entrusted with undergraduate teaching and post-graduate training of medical officers, particularly surgical trainees who were required to undergo neurosurgery rotation in their Master of Surgery programme. The Unit was strengthened with the return of Dr Ahmad Khan Ibrahim Khan in 1986 after completing his training in Leuven, Belgium followed by Dr Halili Rahmat in 1988 after his training in Melbourne, Australia under Dr Kelvin Siu. This situation did not last very long with the resignation of Dr Kazem in 1986 to join Lam Wah Ee Hospital in Pulau Pinang and Dr Ahmad Khan joining Johor Specialist Hospital in 1989. Dr Fadzli Cheah Abdullah resigned and joined Ipoh Specialist Centre at the beginning of 1990 whilst Dr Halili Rahmat also left for Tawakal Specialist Hospital in 1992.

Neurosurgical service in UKM continued with the arrival of Dr Benedict Selladurai in 1993 following his resignation from USM. Dr Selladurai played a vital role in upgrading the neurosurgical service and training in UKM. He has a keen interest in the management of Head Injury and has co-authored a book ‘Head injury — a comprehensive guide for initial management’ published by McGraw-Hill (Sydney) in 2007 which was awarded as ‘a highly recommended title’ by British Medical Association (15). He also has a special interest in epilepsy surgery. He was appointed as Professor of Neurosurgery in 1995 — a distinct honor of being the first Professor of Neurosurgery in Malaysia. Dr Selladurai had a profound influence on his trainees at that time, viz. Dr Abdul Muin Ishak, Dr Zurin Adnan Abd Rahman and Dr Jegan Thanabalan. Dr Selladurai left UKM for practice in Brunei in 1998. He rejoined UKM in 2001 until 2006 when he decided to go into private practice in Subang Jaya Medical Centre.

Dr Abdul Muin received further training in the Institute of Neurosciences, Glasgow and returned in 1994 to assume the role of Head of Division of Neurosurgery. He left UKM and joined KPJ Ampang Puteri Specialist Hospital in 1999. Dr Zurin Adnan Abd Rahman obtained his Master of Surgery (UKM) in 1995. He served as clinical specialist in the Division of Neurosurgery from 1995 to 1997. This was followed by a 2-year clinical fellowship in Neurosurgery and Spinal Neurosurgery in the Royal North Shore Hospital, Sydney under Professor Michael Morgan. He returned to UKM in 1999 and took over as the Head of the Division until 2006. He resigned in September 2006 and started private practice in KPJ Damansara Specialist Hospital.

**Figure 23:**
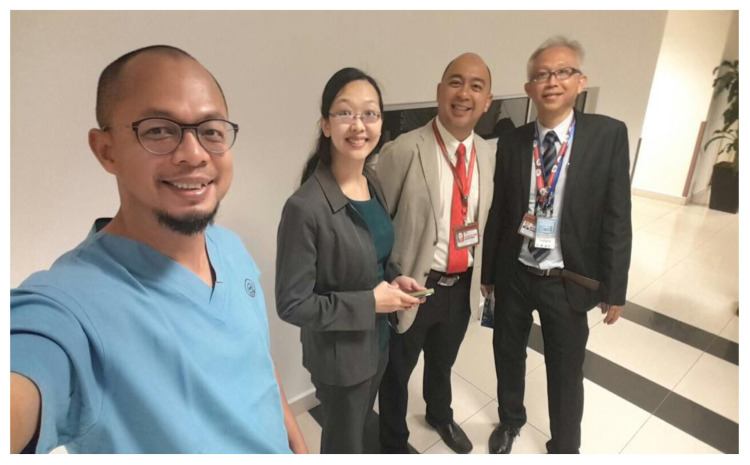
Photos of the current neurosurgeons in UKM (from left to right): Dr Azizi Abu Bakar (head of department), Dr Soon Bee Hong, Dr Ainul Syahrilfazli Jaafar and Dr Toh Charng Jeng

**Figure 24:**
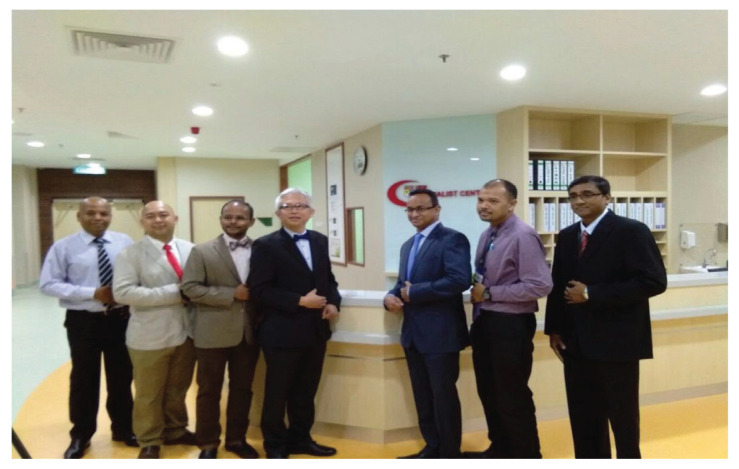
Photos of the current neurosurgeons in UKM (from left to right): Dr Sanmugarajah Paramasvaran, Dr Ainul Syahrilfazli Jaafar, Dr Kamalanathan Palaniandy, Dr Toh Charng Jeng, Dr Ramesh Kumar Athi Kumar, Dr Farizal Fadzil and Dr Jegan Thanabalan

Dr Zurin Adnan Abd Rahman was succeeded by Dr Azizi Abu Bakar who returned from Australia in 2006 after completing his Vascular Fellowship under Dr Michael K Morgan in University of Sydney and Paediatrics Neurosurgery Fellowship under Dr Raymond Chaseling, Children Hospital, Parramatta, Sydney. Dr Azizi Abu Bakar himself is a local graduate who obtained the Master of Surgery UKM in 2000. The division of neurosurgery at UKM has always played a role in the training of local neurosurgeons and currently has a complement of nine neurosurgeons, namely Dr Azizi Abu Bakar (head of department), Dr Soon Bee Hong, Dr Sanmugarajah Paramasvaran, Dr Ainul Syahrilfazli Jaafar, Dr Kamalanathan Palaniandy, Dr Toh Charng Jeng, Dr Ramesh Kumar Athi Kumar, Dr Farizal Fadzil and Dr Jegan Thanabalan (Figures 23 and 24).

### Neurosurgery in Universiti Sains Malaysia

In 1984, Dr Fauzi Ahmad Ali Salem from Egypt joined Universiti Sains Malaysia (USM) Hospital in Kota Baharu, Kelantan, as its first neurosurgeon. He was mostly involved in the management of acute head injuries that required urgent neurosurgical intervention. Following his departure in 1987, there was a lapse of three years before Dr Benedict Marius Selladurai from Sri Lanka joined in 1990. He was followed by Dr Sahanmugan Chandrasekaran in 1992. Dr Benedict Selladurai left USM to join UKM in 1993 and Dr Chandrasekaran left in 1996.

**Figure 25:**
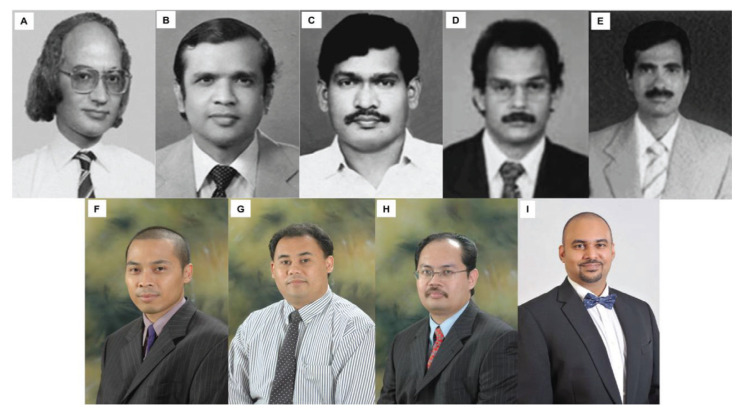
Photos of previous neurosurgeons at USM Kubang Kerian. (A) Dr Fauzi Ahmad Ali Salem (Egypt; 1984–1987), (B) Dr Benedict Marius Selladurai (Sri Lanka; 1990–1993), (C) Dr Shanmugan Chandrasekaran (India; 1992–1996), (D) Dr Jain George Panattil (India; 2002–2005), (E) Dr Prakash Rao Gollapudi (India; 2004–2005), (F) Dr Sani Sayuthi (USM graduates; 2001–2009), (G) Dr Mohamed Saufi Awang (USM graduates; 2001–2010), (H) Dr Badrisyah Idris (USM graduates; 2004–2019) and (I) Dr Regunath Kandasamy (USM graduates; 2012–2020)

**Figure 26:**
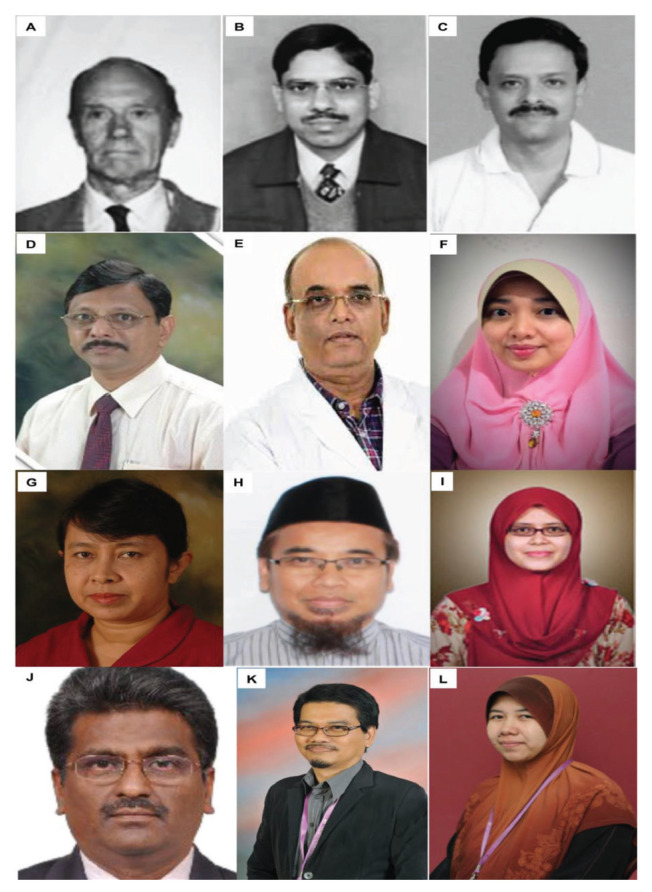
Visiting lecturers at USM Kubang Kerian. (A) Professor Luc Calliauw, (B) Dr Raj Kumar and (C) Dr Hillol Pal. The neurologist includes (D) Dr John Tharakan, (E) Dr Atul Prasad and (F) Dr Sanihah Abdul Halim. The neuroradiologist includes (G) Dr Win Mar @ Salmah Jalaludin, (H) Dr Mohd Shafie Abdullah and (I) Dr Nur Asma Sapiai. The neuropathologist includes (J) Dr Manoharan Madhavan, (K) Dr Hasnan Jaafar and (L) Dr Anani Aila Mat Zin

In May 1995, Dr Jafri Malin Abdullah returned to serve his alma mater after completing his six-year neurosurgical residency training at the University of Ghent, Belgium, under Professors Luc Calliauw and Jacques Caemart, as well as a Stereotactic, Functional and Pain Fellowship with the esteemed Leksell Elekta Belgium Scholarship at the Karolinska Institute, University Hospital, Stockholm, Sweden under Professors Bjorn Meyerson and Bengt Linderoth with a short skull base training programme with Professor Albert Rhoton Jr at the University of Florida, Gainesville, USA. The arrival of Dr Jafri Malin Abdullah ushered in a new era of development for neurosciences, particularly neurosurgery, at USM. The Department of Neurosciences which is the only department of its kind in South East Asia in that period was subsequently established on 17 August 2000, headed by Dr Jafri Malin Abdullah himself. The department attracted the participation of more neurosurgeons, with Dr George Jain joining in 2002 and Dr Prakash Rao in 2004 (Figure 25). Professor Luc Calliauw (Figure 26A) was appointed to the department as a visiting professor. His invaluable experience as past president of the European Association of Neurological Surgeons (EANS) provided extensive guidance to young trainees in neurosurgery. Other visiting lecturers who became the foundation of development in neurosurgical training in Malaysia include Dr Raj Kumar (Figure 26B) and Dr Hillol Kanti Pal (Figure 26C) from India, who came over in 2005. Other department that supported the training of neurosurgical residents include:

- Neurology training
  Dr John Tharakan (Figure 26D)
  Dr Atul Prasad (Figure 26E)
  Dr Sanihah Abdul Halim (Figure 26F)
- Neuroradiology training
  Dr Win Mar @ Salmah Jalaludin (Figure 26G)
  Dr Mohd Shafie Abdullah (Figure 26H)
  Dr Nur Asma Sapiai (Figure 26I)
- Neuropathology training
  Dr Manoharan Madhavan (Figure 26J)
  Dr Hasnan Jaafar (Figure 26K)
  Dr Anani Aila Mat Zin (Figure 26L)

**Figure 27:**
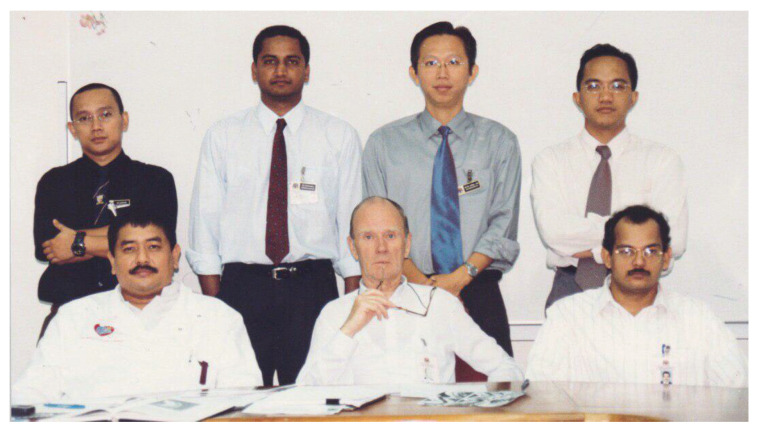
The first batch of Master of Neurosurgery USM with supervisors. Standing from left is Dr Zamzuri Idris, Dr Kanakaraj (left neurosurgery training programme), Dr Toh Charng Jeng and Dr Adam Zakaria. Sitting form left is Dr Jafri Malin Abdullah, Professor Luc Calliauw and Dr Jain George Panattil

**Figure 28:**
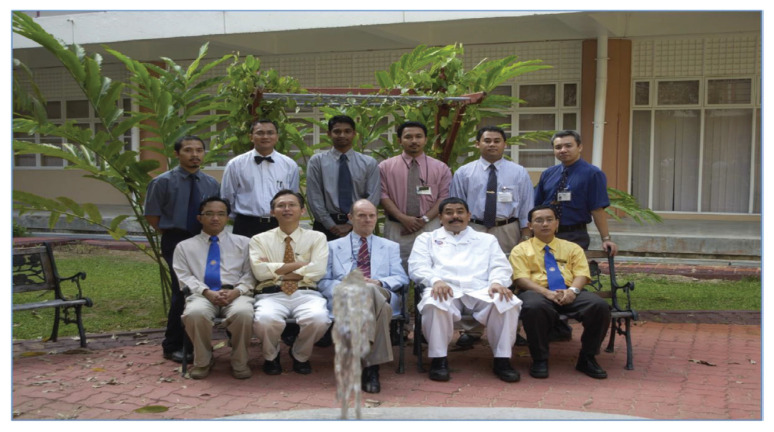
Professor Luc Calliauw was a visiting professor from University of Ghent, Belgium. The photo was taken with the first till third batch of neurosurgical residents at USM Kubang Kerian

Dr Jafri Malin Abdullah, with sheer determination and ingenuity, successfully transformed the department into a state-of-the-art clinical neuroscience centre combining neurosurgery, neurology equipment, and critical care intensive care facilities, a leading academic centre for postgraduate training in neurosciences (neurosurgery in particular) and a research hub for fundamental and clinical neurosciences. In 2001, a comprehensive 2-plus-4-year post-graduate Master of Neurosurgery training programme was launched at USM with the collaboration of the Department/Unit of Neurosurgery of different hospitals under the MOH and MOHE Malaysia, the Malaysian Qualifying Agency and the full cooperation of the NAM (Figure 27). In October 2004, Professor Luc Calliauw was the visiting professor for the first six batches of neurosurgical residents at USM Kubang Kerian, Kelantan (Figure 28).

**Figure 29:**
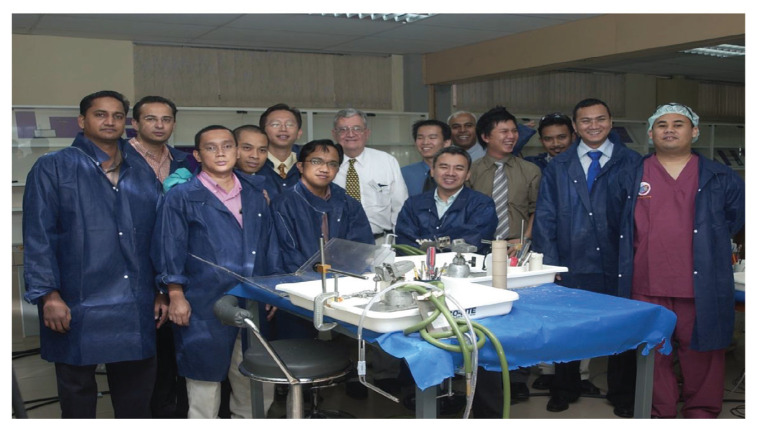
High Speed Drill course was held at USM in 2004. The course was in association with Mr Bob Bullard of Medtronics USA, Mahesh Swaminathan Vas, Kelvin Lim and Bong Joong of Medtronic Malaysia. The course was attended by the first till third batch or neurosurgical residents

**Figure 30:**
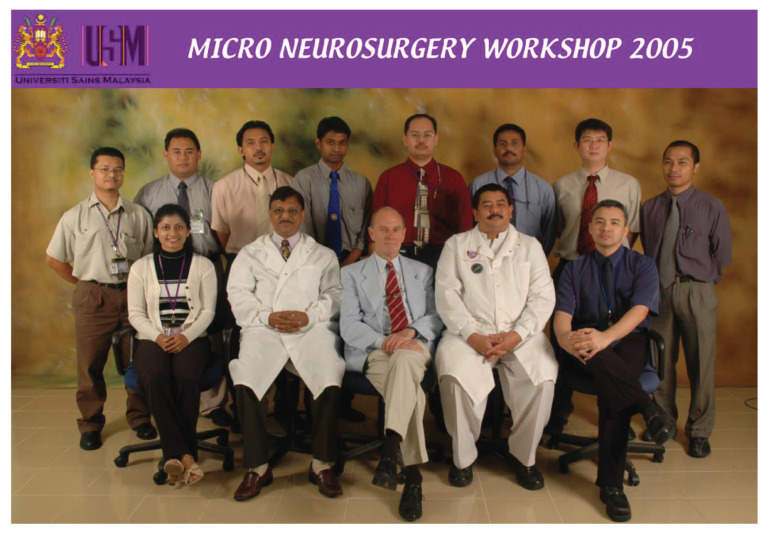
Microsurgery workshop was held at USM in 2005. The course was attended by the first till fourth batch of neurosurgical residents

This training centre opened an opportunity to introduce USM to the world establishing different specialties from cerebrovascular neurosurgery and interventional neuroradiology, paediatric neurosurgery, stereotactic radiosurgery, spine and peripheral nerve surgery, skull base neurosurgery, interventional pain therapy, neurooncology, stereotactic and functional image guided neurosurgery, deep brain stimulation and epilepsy surgery, making this department the only recognised training centre under the World Federation of Neurosurgical Societies (WFNS). As part of the continuing professional education and keeping abreast with neurosurgical skill, from the beginning, USM was able to secure places for courses, such as a high-speed drill course in October 2004 (Figure 29), followed by a microsurgery workshop in 2005 (Figure 30). To date, the courses remain open not just to USM residents but also to others. Collaboration with other hospitals internationally was strong in order to maintain a high standard of graduates in the Master of Neurosurgery USM training programme.

**Figure 31:**
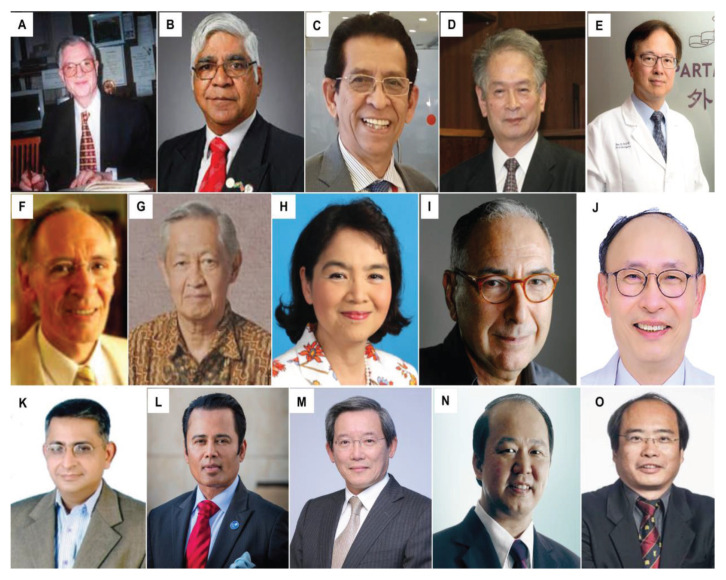
List of external examiners from 2002 until 2019. (A) Dr Iftikhar Ali Raja from Pakistan, (B) Dr Ganapathy Krishnan from India, (C) Dr Abdul Hafid Bajamal from Indonesia, (D) Dr Tetsuo Kanno from Japan, (E) Dr Wai Sang Poon from Hong Kong, (F) Dr Peter Reilly from Australia, (G) Dr Beny Atwadja Wirjomartani from Indonesia, (H) Dr Yoko Kato from Japan, (I) Dr Jeffrey Victor Rosenfeld from Australia, (J) Dr Kyu-Chang Wang from Korea, (K) Dr Syed Ather Enam from Pakistan, (L) Dr Saleem Abdulrauf from the USA, (M) Dr Yong-Kwang Tu from Taiwan, (N) Dr Eka Julianta Wahjoepramono from Indonesia and (O) Dr Yeo Tseng Tsai from Singapore

**Figure 32:**
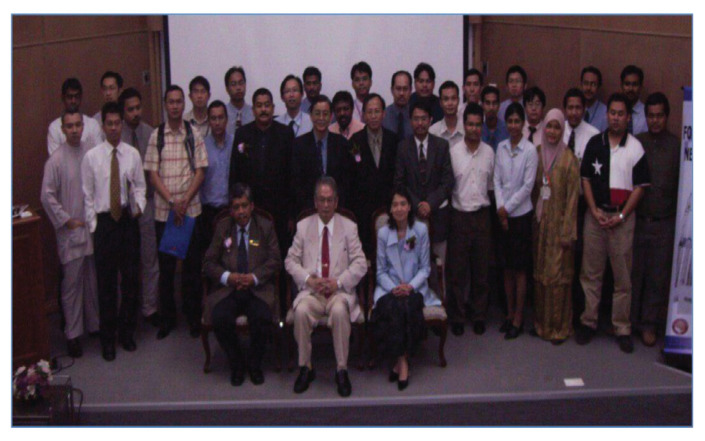
Professor Yoko Kato and Professor Tetsuo Kanno at Hospital Kuala Lumpur. They were the invited external examiner for master in neurosurgery exam in May 2006

**Figure 33:**
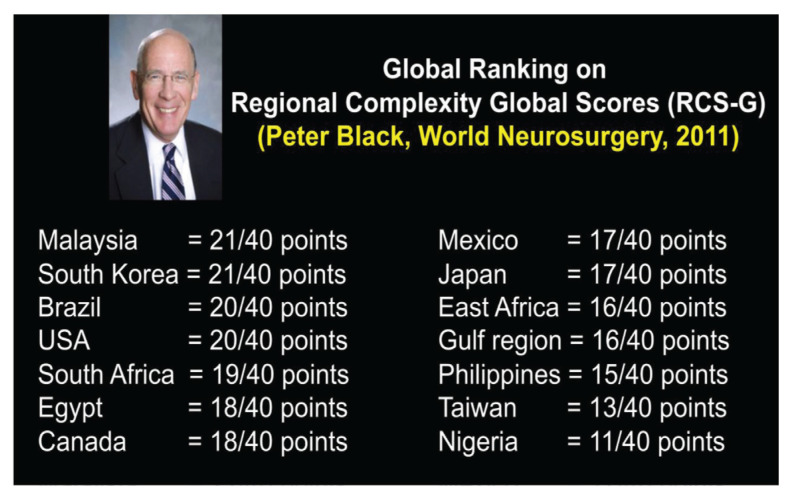
Neurosurgery certification in member societies of the WFNS: Asia

All final year examinations were done twice a year and in the presence of an external examiner from 6 continents. The first external examiner was the late Dr Iftikhar Ali Raja from Pakistan who was invited for 2002, 2003 and 2005 (Figure 31A). Dr Ganapathy Krishnan from India was invited for 2004, 2006 and 2007 (Figure 31B), Dr Abdul Hafid Bajamal from Indonesia for 2004 and 2015 (Figure 31C), Dr Tetsuo Kanno from Japan for 2006 (Figures 31D and 32), Dr Wai Sang Poon from Hong Kong for 2008, 2010, 2012 and 2017 (Figure 31E), Dr Peter Reilly from Australia for 2009 (Figure 31F), Dr Beny Atwadja Wirjomartani from Indonesia for 2011 (Figure 31G), Dr Yoko Kato from Japan for 2006, 2013 and 2019 (Figures 31H and 32), Dr Jeffrey Victor Rosenfeld from Australia for 2014 and 2015 (Figure 31I), Dr Kyu-Chang Wang from Korea for 2014 (Figure 31J), Dr Syed Ather Enam from Pakistan for 2016 (Figure 31K), Dr Saleem Abdulrauf from the USA for 2017 (Figure 31L), Dr Yong-Kwang Tu from Taiwan for 2018 (Figure 31M), Dr Eka Julianta Wahjoepramono from Indonesia for 2018 (Figure 31N) and Dr Yeo Tseng Tsai from Singapore for 2019 (Figure 31O). In 2011, Dr Peter Black presented his analysis comparing the complexity and diversity of the certification process in neurological surgery in member societies of the WFNS. In Asia, the greatest RCS-G score was achieved by Malaysia and South Korea (21/40 points), followed by the joint examination of Singapore and Hong Kong (FRCS-Ed; 20/40 points), Japan (17/40 points), the Philippines (15/40 points) and Taiwan (13 points, Figure 33) (16).

**Figure 34:**
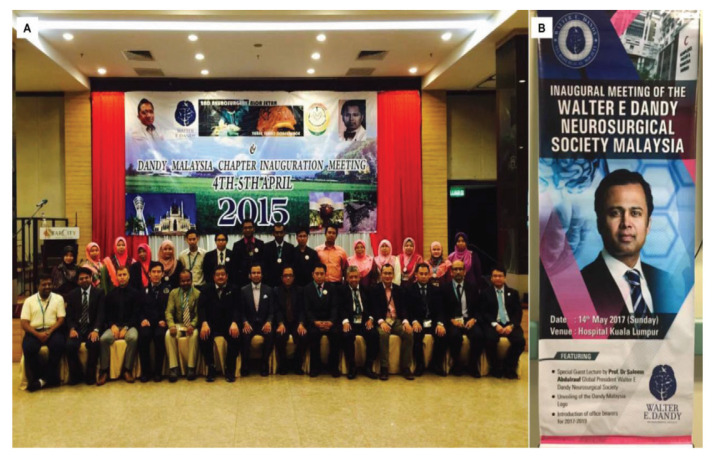
(A) In April 2015, The Dandy Malaysia Chapter Inauguration Meeting was held in Kedah, (B) In May 2017, the inaugural opening the Walter E Dandy Neurosurgical Society Malaysia was held in HKL

**Figure 35:**
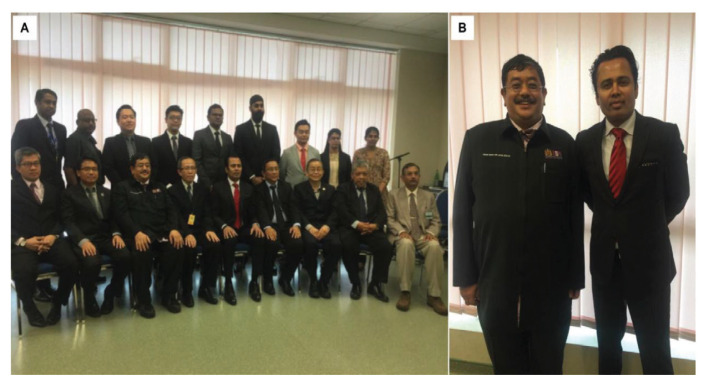
(A) Dr Saleem Abdulrauf was the invited external examiner for Master in Neurosurgery exam in May 2017, (B) Dr Jafri Malin Abdullah with Dr Saleem Abdulrauf

Dr Saleem Abdulrauf is the Global Dandy President for The Walter E Dandy Neurosurgical Society founded on 19 November 2011 in St. Louis, Missouri. In April 2015, the Dandy Malaysia Chapter Inauguration Meeting was held in Kedah (Figure 34A). Following that, in May 2017, The Walter E Dandy Neurosurgical Society Malaysia was established. The inaugural opening was held in HKL with speech and lecture titled ‘Awake craniotomy in aneurysm, AVM and bypass cerebrovascular surgery’ (Figure 34B). The president for Malaysian chapter is Dr Abdul Rahman Izaini Ghani from USM, followed by Dr Saufi Awang as the vice president, Dr Regunath Kandasamy as the secretary and Dr Ananda Arumugam as the treasurer. At the same, the final year examination for May 2017 was held in HKL and Dr Saleem was invited as the external examiner (Figure 35).

**Figure 36:**
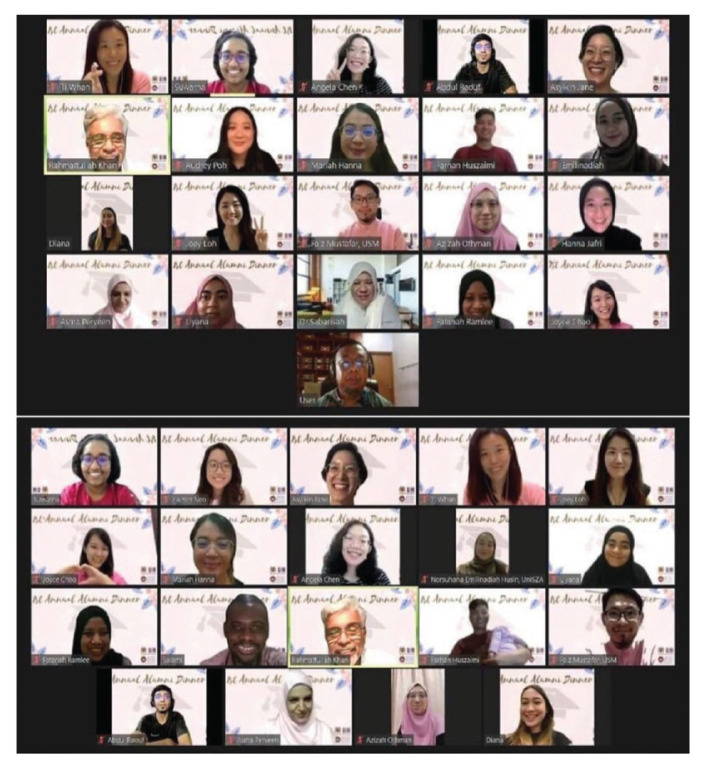
The first batch of the clinical psychologist helping neurosurgeons in Malaysia

The department has a combination of different specialists from neurology, neurorehabilitation, neuroanesthesiologist, clinical neuropsychologist and neuroscientists. The vast experience from other specialties is a great backbone for a successful training programme. By the end of 2021, the USM Department of Neurosciences had produced 103 neurosurgeons. An integrated neuroscience programme (INP) was also established nine years ago which produced more than 58 graduates with Master of Neuroscience (M Neurosc) degree (mixed mode). Twenty Master of Sciences (Neurosciences: pure research mode) graduates were produced with six PhD graduates pure research mode in Neurosciences as well as 10 Doctor of Neurosciences (Neurosc D) (INP) till July 2021. A Master of Cognitive Neurosciences and an Integrated Master of Psychology (Clinical), Doctorate Psychology (Clinical Psychology) and Doctorate Psychology (Clinical Neuropsychology) was established in 2019 (Figure 36). To date, there were 19 graduates from the first and second batches in the Master of Psychology (Clinical) and 21 graduates from Master of Cognitive Neurosciences.

The fast expansion of the department is in line with the dedication and prospered leadership by Dr Jafri Malin Abdullah. Indeed, Dr Jafri Malin Abdullah had the foresight and had accelerate a diverse clinical, laboratory and scientific training. To date, the department has its own neurointensive care, neurorehabilitation unit, operation theatre equipments, electroencephalography (video telemetry), electromyography, deep brain stimulation (DBS) surgery, vagus nerve stimulation (VNS) lab and transcranial magnetic stimulation (TMS) lab, magnetoencephalography (MEG) lab, event related potential, eye tracking system, functional MRI (fMRI) lab, animal and human behaviour/psychology lab, neurogenetics, neuro-electrophysiology lab with patch clamp and xenopus oocyte facilities, and stem cell and neuroregeneration lab.

**Figure 37:**
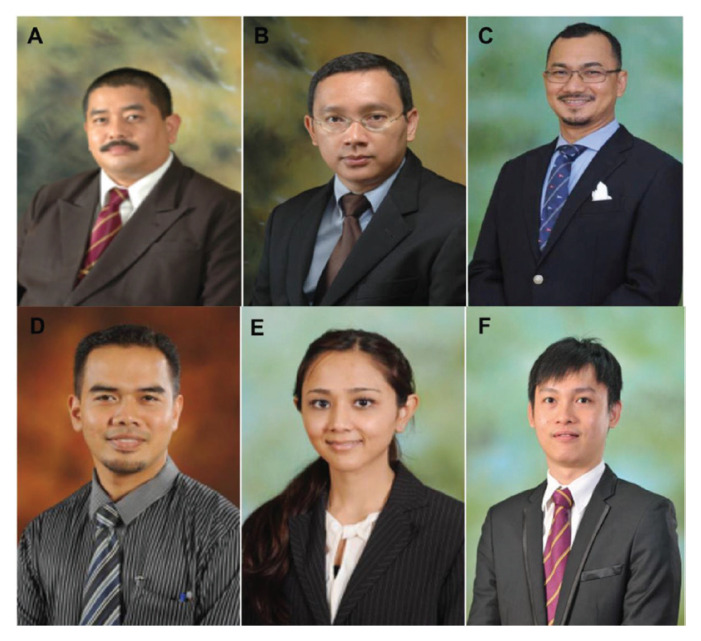
The current neurosurgeons at USM Kubang Kerian. (A) Dr Jafri Malin Abdullah, (B) Dr Zamzuri Idris, (C) Dr Abdul Rahman Izaini Ghani, (D) Dr Muhammad Ihfaz Ismail, (E) Dr Diana Noma Fitzrol and (F) Dr Ang Song Yee

Dr Jafri Malin Abdullah (Figure 37A) was appointed as the Professor of Neurosciences in December 2003. He was transferred from his position as head of the Department of Neurosciences in August 2015 to the chair of the Pusat Penyelidikan dan Perkhidmatan Neurosains (P3Neuro), an excellent research and service centre established by decree of the Ministry of Higher Education (MOHE) Malaysia, which was returned by Senate USM to the School of Medical Sciences in the end of 2018. He was succeeded by Dr Zamzuri Idris (Figure 37B), from the first batch of Masters of Neurosurgery USM. Dr Zamzuri Idris is assisted by Dr Abdul Rahman Izaini Ghani (Figure 37C), Dr Muhammad Ihfaz Ismail (Figure 37D), Dr Diana Noma Fitzrol (Figure 37E) and Dr Ang Song Yee (Figure 37F), who are all graduates of the Master of Neurosurgery USM programme. Dr Jafri Malin Abdullah remains active as the senior consulting neurosurgeon and senior professor Grade A of the department, as well as Chairman of the Brain and Behaviour Cluster (BBC), School of Medical Sciences, USM since 1 January 2019. On 17 January 2019, the Neuroinformatic BBC-School of Computer Sciences USM group was established to intergrade the Big Data from fMRI, MRI, EEG, MEG, Deep Brain Recording for advanced neurosurgical-neurological-psychiatric-clinical psychological treatment. The latest progress was on the 30 July 2021, where the Hospital USM Neuro-Clinical STEM cell group was launched. The services and research were expanded to neural stem cells and transplant to treat the peripheral nerve diseases, brain and spinal cord injury ailments.

### Neurosurgery in the International Islamic University Malaysia

**Figure 38:**
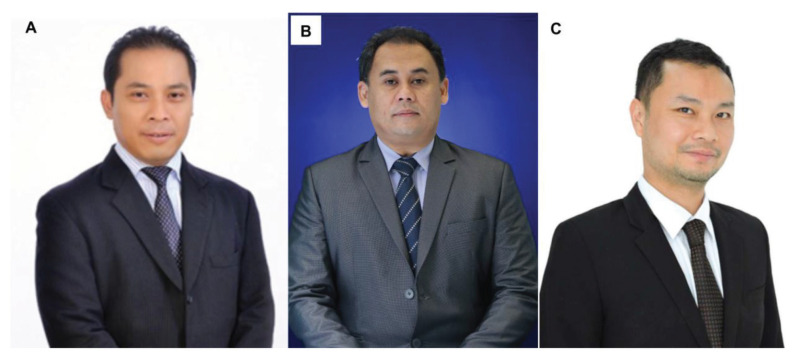
The previous and current neurosurgeons at IIUM. (A) Dr Sani Sayuti, (B) Dr Mohamed Saufi Awang and (C) Dr Chan Kin Hup

The state of Pahang is the largest in Peninsular Malaysia. Transportation has improved with better access by road and air. The public hospital, Hospital Tengku Ampuan Afzan (HTAA), Kuantan is closer to other neurosurgical centres — in the eastern region, Hospital USM Kubang Kerian (about 340 km) and Hospital Tuanku Nor Zahirah Kuala Terengganu (about 220 km), and in the central and southern region IKTAR (about 280 km) and Hospital Sultanah Aminah Johor Bahru (about 340 km). HTAA serves two million people and receives referrals from all districts of the Pahang and Kemaman areas (in Terengganu). Neurosurgery in Pahang started in 2004; its initiation was implemented between the MOH Malaysia and the International Islamic University Malaysia (IIUM). The neurosurgery unit was headed by Dr Khaw (from IIUM) and assisted by a medical officer from the MOH, Dr Khairul Azmi. Subsequently, Dr Sani Sayuti and Dr Mohamed Saufi Awang (Figures 38A and B) joined the unit in 2010, followed by Dr Chan Kin Hup in 2011 (Figure 38C). In 2017, Dr Mohamed Saufi and Dr Chan Kin Hup started the neurosurgical services at IIUM Medical Centre. Dr Chan Kin Hup and Dr Sani Sayuti later left for private practice. Since then, Dr Mohamed Saufi has expanded the service to include teaching and training; it plays an important role in the Master of Neurosurgery USM postgraduate programme.

## Neurosurgery in the Central and Southern Region

### Neurosurgery in Kuala Lumpur

**Figure 39:**
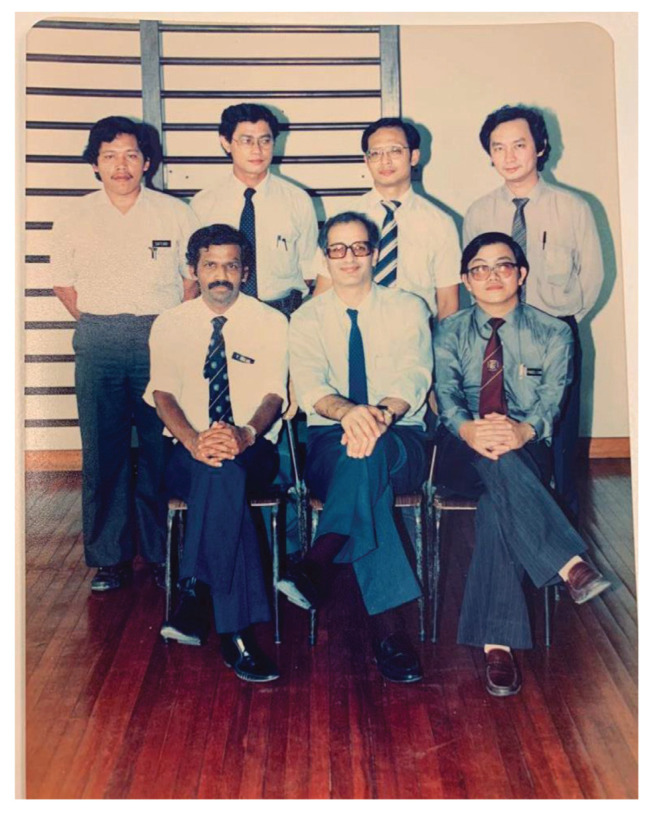
Photos in early 1980s of neurosurgeons working at Hospital Kuala Lumpur. Standing from left is Dr Mohammed Saffari Haspani, Dr Ahmad Zubaidi Abdul Latif, Dr Ahmad Khan Ibrahim Khan and Dr Ho Yau Shen. Sitting from left is Dr Selvapragasam Thambiah, Dr Kazem Djavadkhani and Dr Fadzli Cheah Abdullah

Dr Selvapragasam Thambiah, who received his major training under Dato’ Dr Nadasan Arumugasamy and also spent time at the Institute of Neurosciences in Glasgow, took over as head of the neurosurgery department HKL in 1986 after the retirement of Dato’ Dr Nadasan Arumugasamy. Dr Ho Yau Shen, who was trained in Belgium, returned and served for a few years before moving to Hospital Sultanah Aminah in 1991. Dr Selvapragasam was dedicated to his service until his retirement in 2001 (Figure 39). After his retirement, he provided sessional service to the department for some years and later concentrated on private practice in which he is still active.

**Figure 40:**
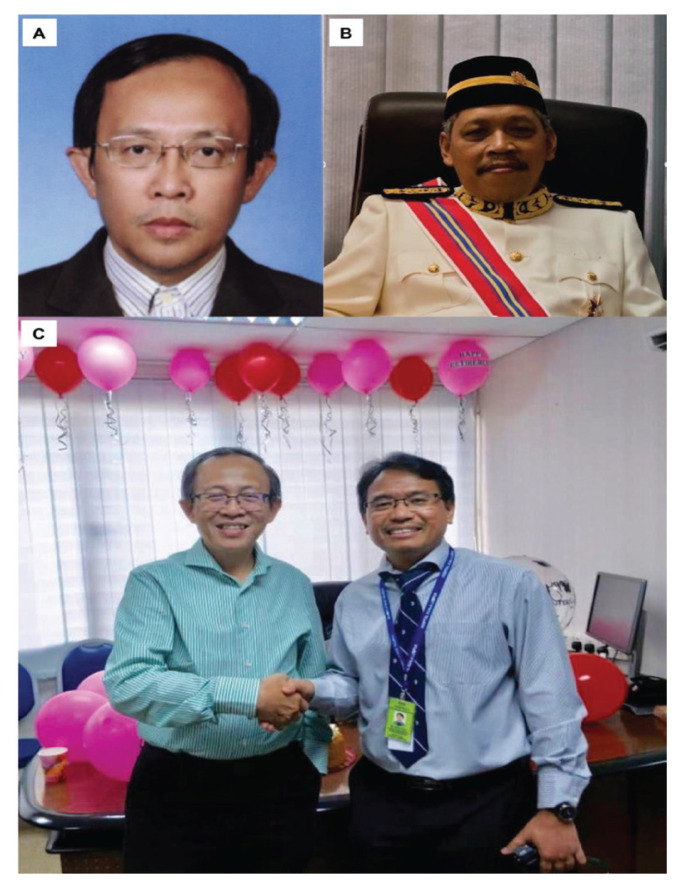
The previous and current head of department in Hospital Kuala Lumpur (A) Dr Johari Siregar Adnan and (B) Dr Mohammed Saffari Haspani were the previous head of department, (C) On 8 October 2019, a small farewell was done for Dr Johari’s retirement and his role was passed to Dr Azmi Alias

Dr Johari Siregar Adnan took over from Dr Selvapragasam as head of the Department of Neurosurgery Hospital Kuala Lumpur (HKL) in 2001 (Figure 40A). He was a local graduate from the UM in 1984 and received his neurosurgery training at the Catholic University of Leuven, Belgium, under Professor Christian Plets from 1989 to 1993. Dr Johari Siregar Adnan was also specially appointed by the MOH as Head of the Neurosurgery Programme responsible for education, training, organisation and development of neurosurgical services nation-wide. Dr Johari Siregar Adnan was instrumental in establishing the post-graduate Master of Neurosurgery training programme in collaboration with USM. He also promoted tele-neurosurgery to allow easy neurosurgery consultation and access from hospitals in remote districts nationwide.

To keep abreast of the rapidly advancing neurosurgery innovations, he also introduced modern facilities such as brain suites in three major hospitals in Johor Bahru, Sungai Buloh, and Kuching. During his tenure as Head of the National Neurosurgery Programme, Dr Johari Siregar Adnan was assisted by Dr Mohammed Saffari Haspani, who also completed his training in Leuven, Belgium, in 1994 (Figure 40B). Dr Mohammed Saffari Haspani was head of the HKL department from 2003 to 2015, when Dr Johari Siregar Adnan was posted to Johor Bahru to head the Neurosurgery Department in Hospital Sultanah Aminah. Dr Johari Siregar Adnan resumed as head of department at HKL in February 2016 until his retirement in October 2019. On 9 October 2019, his role as head of the neurosurgery programme was passed to Dr Azmin Kass Rosman.

Dr Azmi Alias was appointed as the new head of the Department of Neurosurgery, IKTAR, HKL on 1 February 2020 (Figure 40C). Dr Azmi Alias was a local graduate with a Master of Surgery from UKM in 2001. He received his initial neurosurgery training in IKTAR HKL, followed by a one-year fellowship (2004–2005) in neurosurgery at the Johannesburg Gutenberg University of Mainz, Germany under Professor Axel Pernecky. He also undertook a three-month attachment for paediatric neurosurgery and craniofacial surgery at the Women & Children’s Hospital and the Royal Adelaide Hospital, South Australia, in 2007. He has been providing excellent service at IKTAR with special interest in paediatric and endoscopic neurosurgery.

**Figure 41:**
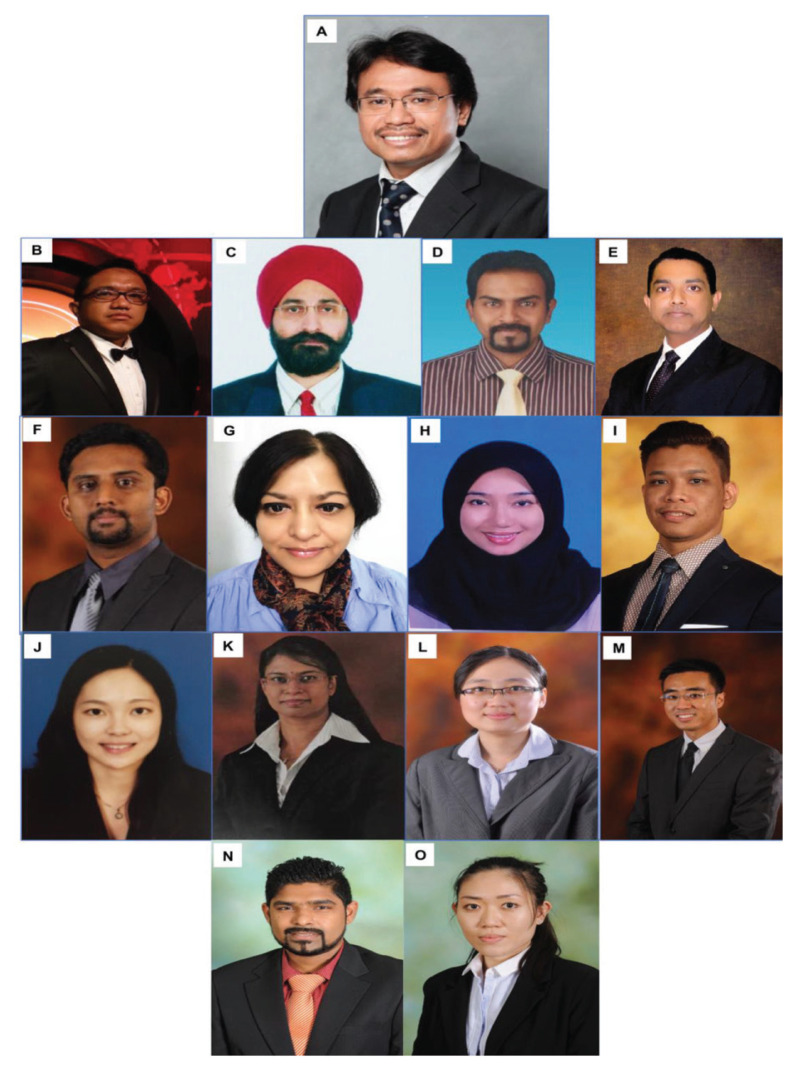
The current neurosurgeons at Hospital Kuala Lumpur. (A) Dr Azmi Alias, (B) Dr Adam Zakaria, (C) Dr Gurmit Singh a/l Attar Singh, (D) Dr Ravindran a/l Vashu, (E) Tinesh Kumaran a/l Jaya Raman, (F) Dr Rajendra Rao a/l Ramalu, (G) Dr Priya Sharda a/p Jagdish Mitter, (H) Dr Siti Azleen Mohamad, (I) Dr Fadzlishah Johanabas Rosli, (J) Dr Lee Chun Lin, (K) Dr Kanmani Devi a/p Ganison, (L) Dr Ng Wei Ping, (M) Dr Yee Sze Voon, (N) Dr Arulkanesh Devatatan and (O) Dr Lim Mei Sin

**Figure 42:**
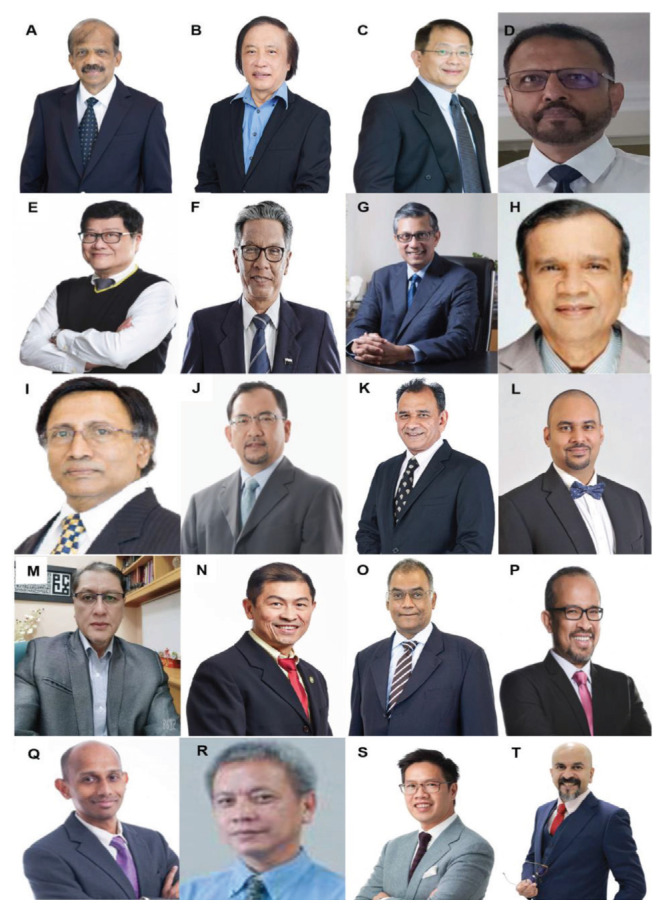
Neurosurgeons in major private hospitals in Kuala Lumpur and Selangor. (A) Dr Selvapragasam a/l Thambiah, (B) Dr Chee Chee Pin, (C) Dr Wong Fung Chu, (D) Dr Muruga Kumar, (E) Dr Lee Foo Chiang, (F) Dr Halili Rahmat, (G) Dr Ravi S a/l Krishnapillai, (H) Dr Benedict Marius Selladurai, (I) Dr Sukumar a/l Sivasubramaniam, (J) Dr Zurin Adnan Abd Rahman, (K) Dr Jagdeep Singh Nanra a/l Kuljit Singh, (L) Dr Regunath Kandasamy, (M) Dr Abdul Muin Ishak, (N) Dr Sia Sheau Fung, (O) Dr N Ramesh a/l Narenthiranathan, (P) Dr Syed Abdullah Al-Hadad, (Q) Dr Gunasegaran Thangaveloo, (R) Dr Lim Heng Tien, (S) Dr Kevin Sek Weng Yew and (T) Dr Gerard Arvind Martin

With ongoing vigorous support and continuing enthusiasm, IKTAR HKL, which currently has 15 neurosurgeons serving the department (Figure 41), is set to maintain its position as the premier neurosurgical referral and training centre in Malaysia, although many private hospitals have sprouted up that provide excellent neurosurgical services. There are currently more than 26 resident neurosurgeons in major private hospitals in Kuala Lumpur and Selangor (Figure 42).

### Neurosurgery in Sungai Buloh

The hospital was developed in 1999 with the main purpose of easing congestion at Hospital Kuala Lumpur of patients needing treatment. The hospital was built 23 km from HKL on the border between Gombak and Petaling districts and became operational on 11 September 2006; neurosurgical services started on 3 November 2006 with the opening of a ward, an outpatient clinic and emergency surgery. The catchment areas are Gombak, Petaling and Kuala Selangor (29% of the total area of Selangor). Two months later, elective cases were added as part of the expansion of neurosurgical services.

**Figure 43:**
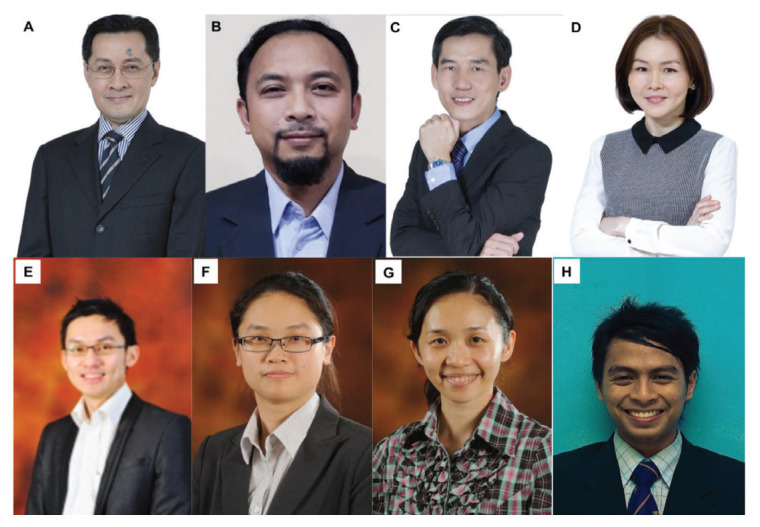
The current neurosurgeons in Hospital Sungai Buloh (A) Dr Azmin Kass Rosman, (B) Dr Saiful Azli Mat Nayan, (C) Dr Liew Boon Seng, (D) Dr Cheah Pooi Pooi, (E) Dr Mah Jon Kooi, (F) You Xin Li, (G) Dr Lee Shwu Yi and (H) Dr Muhammad Aizzat Othman

The first neurosurgeon was Dr Azmin Kass Rosman (Figure 43A). Dr Azmin Kass Rosman was a local graduate who received his early training in IKTAR and later completed his neurosurgery training in Leuven, Belgium, from 1993–1996. After his return, he worked at IKTAR and was later transferred to Penang General Hospital in 1998. He was head of the department in Pulau Pinang until 2005. In 2005, he was posted to Sungai Buloh Hospital, Selangor to establish a new Department of Neurosurgery and continues as head of the department to the present. In 2006, Dr Toh Charng Jeng joined for one year, and in 2007, Dr Saiful Azli Mat Nayan (Figure 43B) joined the department and remains there now. Two years later, Liew Boon Seng (Figure 43C) joined the department who also continues today. These three are the main pillars in the development of neurosurgical services in Sungai Buloh. Dr Gee Teak Sheng served for two years (2011–2013) before moving to a private hospital. Afterwards, other neurosurgeons work there, all of whom are local graduates from the Master of Neurosurgery USM programme. Current neurosurgeons include Dr Cheah Poi Pooi, Dr Mah Jon Kooi and Dr You Xin Li (Figures 43D–43F). Dr Ailani Ghani was at Hospital Sungai Buloh for four years (2015–2019) before moving to Hospital Tengku Ampuan Afzan and Dr Ng Wei Ping (Figure 41L) served for one year (2016–2017) before moving to HKL. Dr Lee Shwu Yi completed the Master in Neurosurgery USM programme in 2019 and Dr Muhammad Aizzat Othman in 2020, have recently joined the department (Figures 43G and 43H).

**Figure 44:**
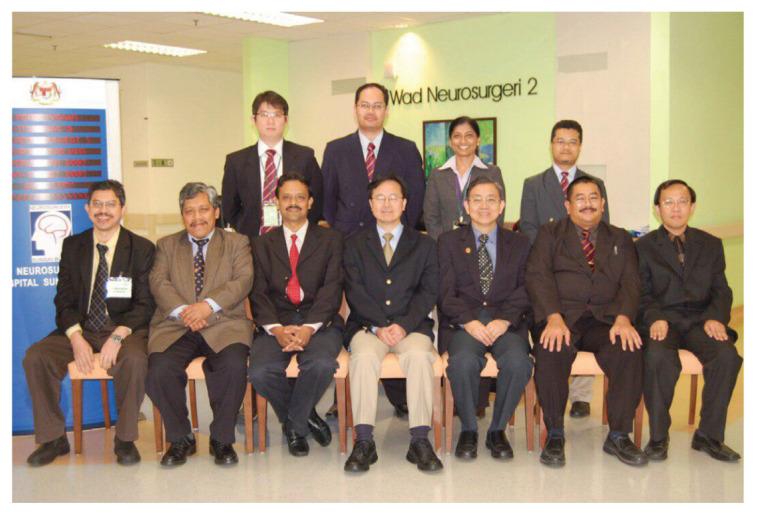
The final year Master in Neurosurgery USM exam 2008 was held at Hospital Sungai Buloh. Standing at the back were the final year trainees (from left to right) Dr Kan Choon Hong, Dr Badrisyah Idris, Dr Sharon Casilda Theophilus and Dr Nujaimin Udin

**Figure 45:**
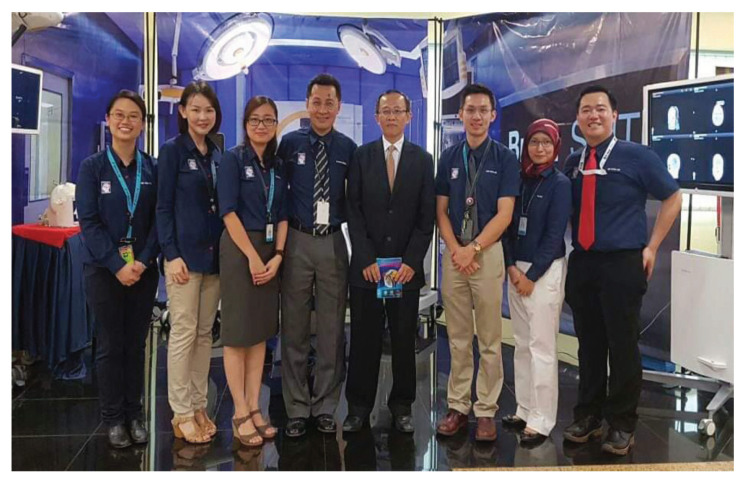
The Virtual Brain Suite event in 2016 organised by the Neurosurgical Department at Hospital Sungai Buloh. From left to right: Dr You Xin Li, Dr Cheah Pooi Pooi, Dr Ng Wei Ping, Dr Azmin Kass Rosman, Dr Johari Siregar Adenan, Dr Mah Jon Kooi, Dr Ailani Abd Ghani and Dr Chan Chee Keong

**Figure 46:**
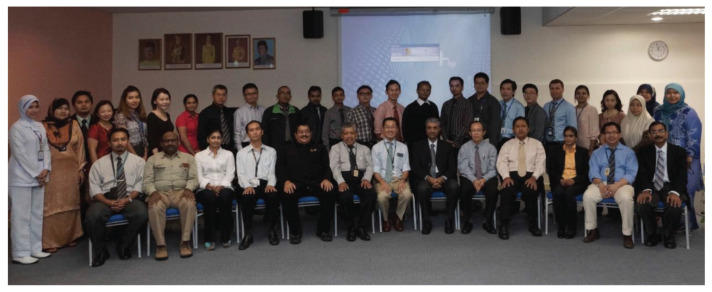
The first Epilepsy Surgery Workshop and Live Surgery at Hospital Sungai Buloh was held on 11–13 February 2014

The Hospital Sungai Buloh Neurosurgery Department has been a regular venue for training courses and professional examination, including the final year examination for Master of Neurosurgery USM, which was first held in 2008 (Figure 44). Since 2010, the hospital has been the first health institution in Malaysia providing a brain suite for intraoperative CT (Figure 45). In 2012, the first deep-brain stimulation via awake surgery for Parkinson’s disease was performed. This was the first surgery at the MOH Malaysia hospital. To 2020, there had been a total of seven successful cases. From the beginning of 2014, the service expanded to include epilepsy cases. To 2020, a total of 65 cases had been performed (Figure 46). The department is also active in research activities, with numerous journal publications and presentations at recognised meetings to its credit.

### Neurosurgery in Johor

**Figure 47:**
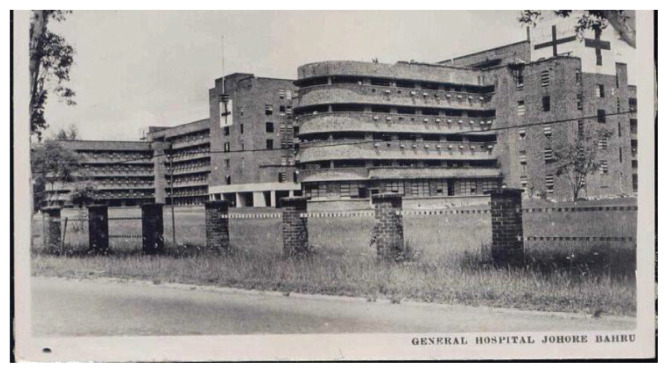
An old photo of Johor Bahru General Hospital

Neurosurgery service was first introduced to Johor Bahru in 1989 when Dr Ahmad Khan Ibrahim Khan joined the Johor Specialist Hospital in the private sector. Neurosurgery as a subspecialty service in the government sector started in 1991 in Hospital Sultanah Aminah Johor Bahru, a magnificent hospital founded in 1882 that faces the Straits of Tebrau (Figure 47). The first head of the Department of Neurosurgery was Dr Ho Yau Shen (Figure 39, standing, right), a local graduate of the UM who was initially trained at IKTAR and later in Leuven, Belgium, under Professor Vanden Berg. Dr Ho Yau Shen started from scratch by sharing wards with the general surgery department and gradually established the present neurosurgery unit. In 1996, Dr Johari Adnan Siregar took over as head of the department (Figure 40). He successfully transformed the department into a well-established regional referral centre equipped with modern facilities that included a state-of-the-art brain suite intraoperative CT.

**Figure 49:**
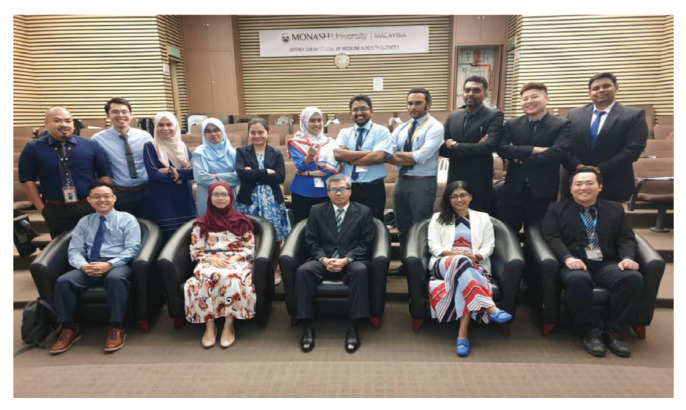
The Johor Neuro-Emergency course 2019 organised by Neurosurgery Department at Hospital Sultanah Aminah Johor Bahru

**Figure 50:**
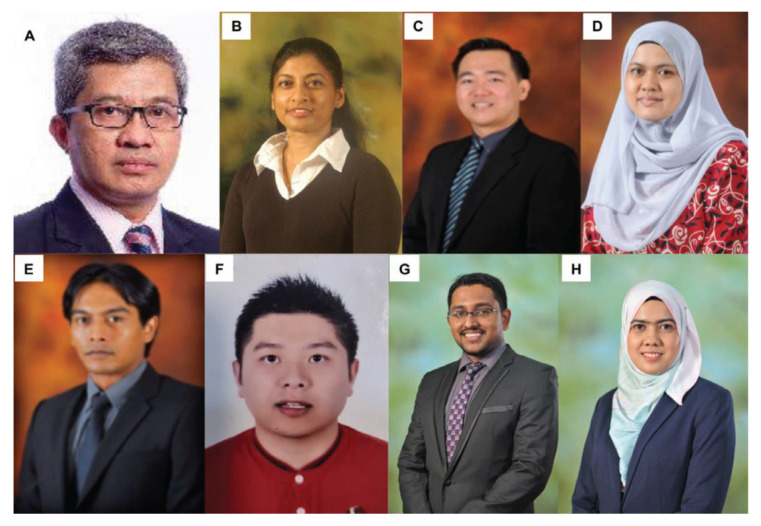
The current neurosurgeons in Hospital Sultanah Aminah Johor Bahru. (A) Dr Noor Azman A Rahman @ Mohd, (B) Dr Sharon Theophilus, (C) Dr Chan Chee Kong, (D) Dr Asma Mohamad Afifi, (E) Dr Rakesh Rethinasamy, (F) Dr Tan Zi Han, (G) Dr Saravanan a/l Sridharan and (H) Dr Nurshaheda Mohd Salleh

Dr Noor Azman A Rahman @ Mohd took over as head of the department in 2016 when Dr Johari Siregar Adnan was transferred back to HKL. Dr Noor Azman A Rahman @ Mohd is a local graduate with a Master of Surgery UKM and completed his neurosurgery training with a fellowship in Melbourne under Dr Andrew Kaye in 2005. He managed to increase the number of staff members and expand the services to respond to the current demand. The department also emerged as a major neurosurgical training centre for the Master of Neurosurgery USM with regular training courses and scientific seminars (Figure 49). Together with Dr Noor Azman A Rahman @ Mohd (Figure 50A), the department has a complement of eight neurosurgeons, including Dr Sharon Theophilus (Figure 50B), who is the first locally-trained female neurosurgeon under the Master of Neurosurgery USM, Dr Chan Chee Kong (Figure 50C), Dr Asma Mohamad Afifi (Figure 50D), Dr Rakesh Rethinasamy (Figure 50E), Dr Tan Zi Han (Figure 50F), Dr Saravanan a/l Sridharan (Figure 50G) and Dr Nurshaheda Mohd Salleh (Figure 50H).

**Figure 51:**
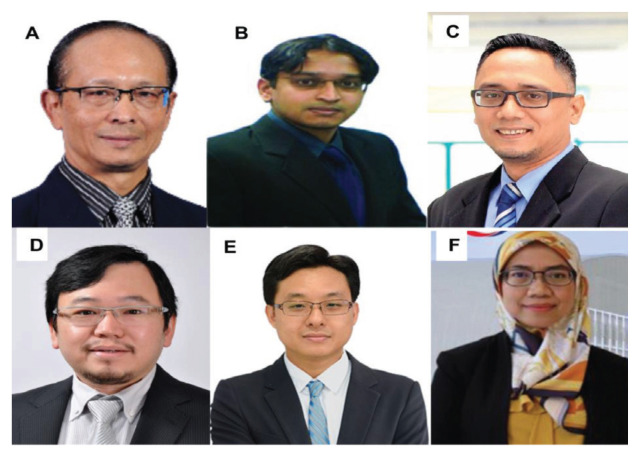
The neurosurgeons in major private hospitals in Johor Bahru. (A) Dr Ahmad Khan Ibrahim Khan, (B) Dr Devaraj Pancharatnam, (C) Dr Ashraf Sharifuddin, (D) Dr Teo Beng Tiong, (E) Dr Ariz Chong Abdullah @ Chong Chee Yong and (F) Dr Risdhawati Hassan

The private sector also plays an active role in providing neurosurgical service, starting with Dr Ahmad Khan since 1989. There are currently five other young neurosurgeons practicing in different private hospitals in Johor Bahru (Figure 51). Three of them, Dr Ashraf Sharifuddin (Figure 51C), Dr Ariz Chong Abdullah (Figure 51E) and Dr Risdhawati Hassan (Figure 51F), also trained under the Master of Neurosurgery USM programme. They had previously worked at Hospital Sultanah Aminah for a few years before embarking into private practice.

### Neurosurgery in Negeri Sembilan

**Figure 52:**
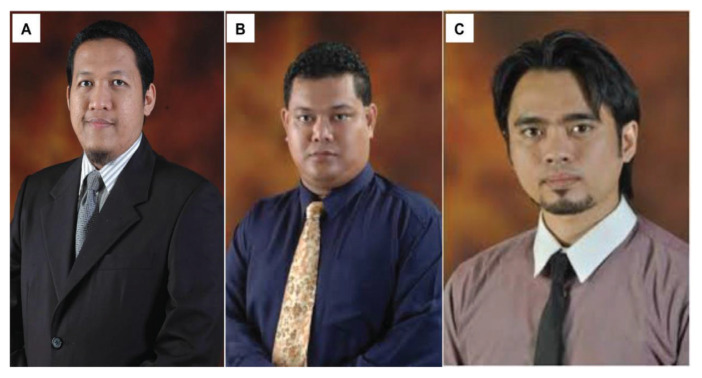
The neurosurgeons currently working in Negeri Sembilan. (A) Dr Mohd Hafiz Mohamad Zain (private sector), (B) Dr Rahmat Harun @Haron (head department of neurosurgery) and (C) Dr Faizul Hizal Ghazali

**Figure 53:**
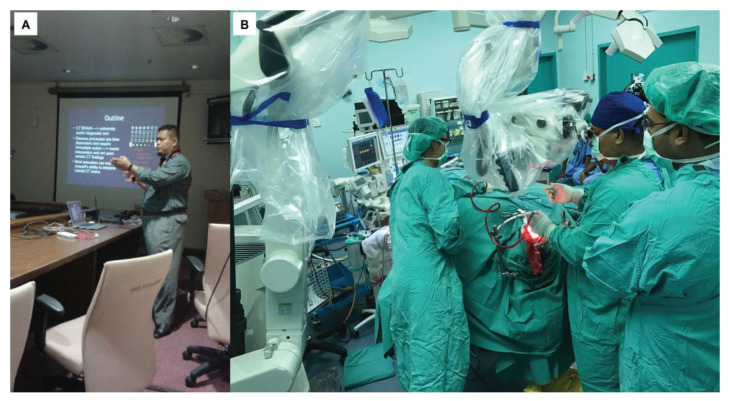
(A) First introduction of the neurosurgical service at Hospital Tuanku Ja’afar (B) At the beginning, some equipment including the microscope and brain retractor system (DORO LUNA) were borrowed

**Figure 54:**
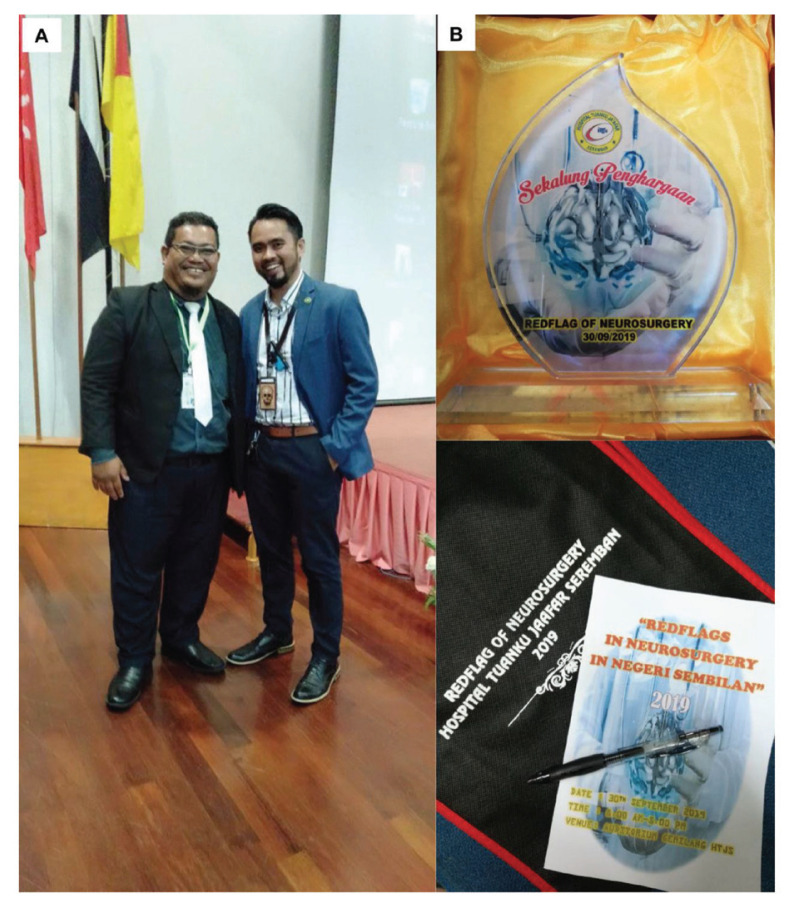
(A) Dr Rahmat Harun @Haron was subsequently joined by Dr Faizul Hizal Ghazali in April 2018, (B) A redflag of neurosurgery course held in Negeri Sembilan in 2019

Neurosurgery service was first available in Negeri Sembilan when Dr Mohd Hafiz Mohamad Zain (Figure 52A) joined KPJ Seremban Specialist Hospital in the private sector in June 2013. Dr Mohd Hafiz is a local USM graduate. He worked at IKTAR HKL prior to embarking into private practice. Neurosurgical service began in the government sector when Dr Rahmat Harun @Haron was posted to Hospital Tuanku Ja’afar, Seremban (HTJS) in May 2016 (Figure 52B). At that time, there were no trained neurosurgical nurses or technicians. Neurosurgical procedures and services are under the umbrella of the Department of General Surgery. Dr Rahmat Harun @Haron managed to borrow some equipment, including the operating microscope and brain retractor system (DORO LUNA) and trained the operating theatre staff (Figure 53). In April 2018, Dr Rahmat Harun @Haron was joined by Dr Faizul Hizal Ghazali who is also a USM graduate (Figures 52C and 54A). Since that time, services have expanded, and it is now able to accept neurosurgical trainees and organise courses. The first neurosurgical course in Negeri Sembilan was conducted on 30 September 2019 titled ‘Redflag of Neurosurgery in Negeri Sembilan’ (Figure 54B).

### Neurosurgery in Melaka

**Figure 55:**
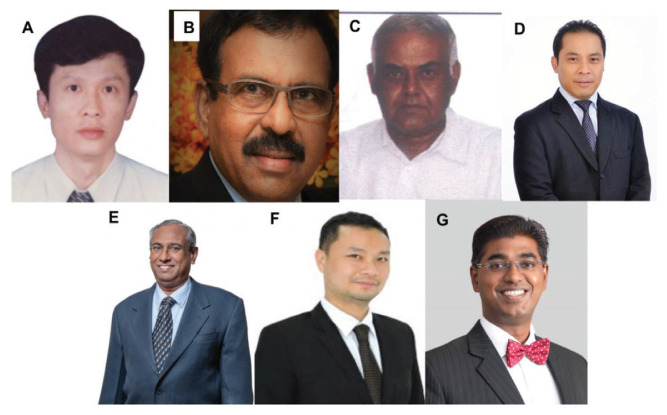
Previous and current neurosurgeons in major private hospitals in Melaka. (A) Dr Chee Wee Liam, (B) Dr M Nachiappan a/l Murugavadigal, (C) Dr Ravi Mamamurthi, (D) Dr Sani Sayuthi, (E) Dr MK Radha Krishnan, (F) Dr Chan Kin Hup and (G) Dr Parthiban Navoo

Neurosurgical service began in Melaka in 1995 when Dr Lim Heng Tien (Figure 42R) resigned from UM and joined the Mahkota Medical Centre. Dr Lim Heng Tien is a local graduate from UM who obtained his FRCS in Edinburgh and subsequently undertook his neurosurgery training in Auckland, New Zealand, under Dr Graeme McDonald. He moved his service to Tung Shin Hospital in Kuala Lumpur in 1998. His position at Mahkota Medical Centre was taken over by Dr Chee Wee Liam (Figure 55A) who is also a local graduate of UM who received his neurosurgery training in Singapore and subsequently in Cambridge, UK, under Dr John Pickard. He was active in Mahkota Medical Centre until 2007 and currently has his own clinic serving several private hospitals.

In 1996, another neurosurgeon, Dr M Nachiappan a/l Murugavadigal (Figure 55B), joined Hospital Pantai Ayer Keroh, Melaka. Dr Nachiappan also held a FRCS from Edinburgh and trained in Singapore and later in Oxford, UK, with an interest in stereotactic neurosurgery. In 2000, Dr Ravi Ramamurthi (Figure 55C) came from India to work for a few years. He returned to India and is currently chairman of Dr Achanta Lakshmipathi Neurosurgical Centre, Voluntary Health Services, Chennai (17). In 2013, Dr Nachiappan left the private sector and joined the Manipal Medical College in Melaka as a full-time lecturer/professor. In total, there are six neurosurgeons in different places in Melaka, including Dr Sani Sayuthi (Figure 55D), Dr MK Radha Krishnan (Figure 55E), Dr Chan Kin Hup (Figure 55F) and Dr Parthiban Navoo (Figure 55G).

## Neurosurgery in the Northern Region

### Neurosurgery in Pulau Pinang

Pulau Pinang was the first state outside Kuala Lumpur to acquire the services of a neurosurgeon when Dr Swaran Singh Khera (Figure 15), one of the local pioneers trained by Dr Roy Clifton Selby (Figure 11), was posted to Penang General Hospital in 1972. He faced multiple challenges and a lack of support facilities. He emigrated to Adelaide, Australia, in 1979. There was no neurosurgeon in the state of Pulau Pinang for almost seven years until Dr Gunasekaran started his private practice in Gleneagles Hospital in 1986. He was soon joined by Dr Kazem Djavadkhani (Figures 22 and 39) in private practice at the Lam Wah Ee Hospital in the latter part of 1986.

Neurosurgical service resumed in Penang General Hospital when Dr Yoong Meow Foong was appointed as the new head of the Department of Neurosurgery in 1994 upon his return from Australia after completing his neurosurgery training at Royal Adelaide Hospital under Dr Brian North. In 1995, another young neurosurgeon, Dr Mathew Tung Yu Yee, returned from the UK and joined Lam Wah Ee Hospital in private practice. Dr Yoong resigned from government service in 1998 and joined Penang Island Hospital to pursue his private practice.

**Figure 56:**
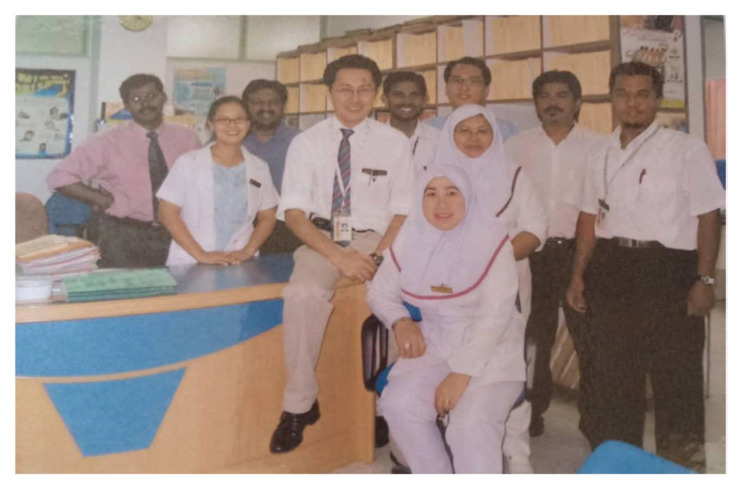
Dr Azmin Kass Rosman became the third neurosurgeon leading neurosurgical department at Penang General Hospital. The photo was taken during his leadership between 1998–2005

**Figure 57:**
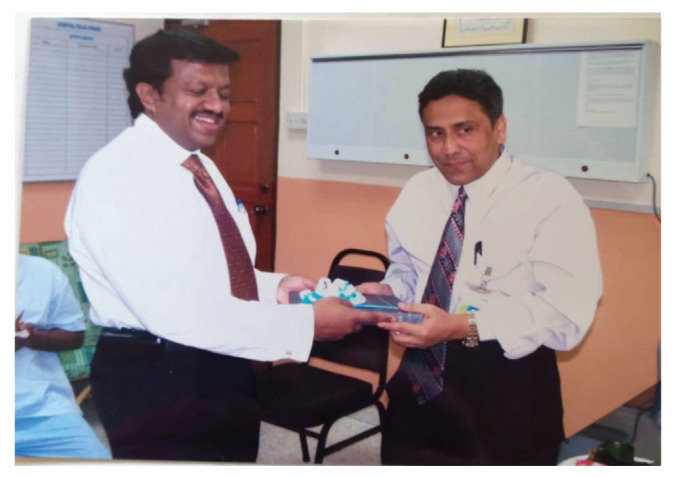
Dr Ravindran Katheerayson became the fourth neurosurgeon leading neurosurgical department at Penang General Hospital. The photo was taken with Director of Penang General Hospital in 2005

**Figure 58:**
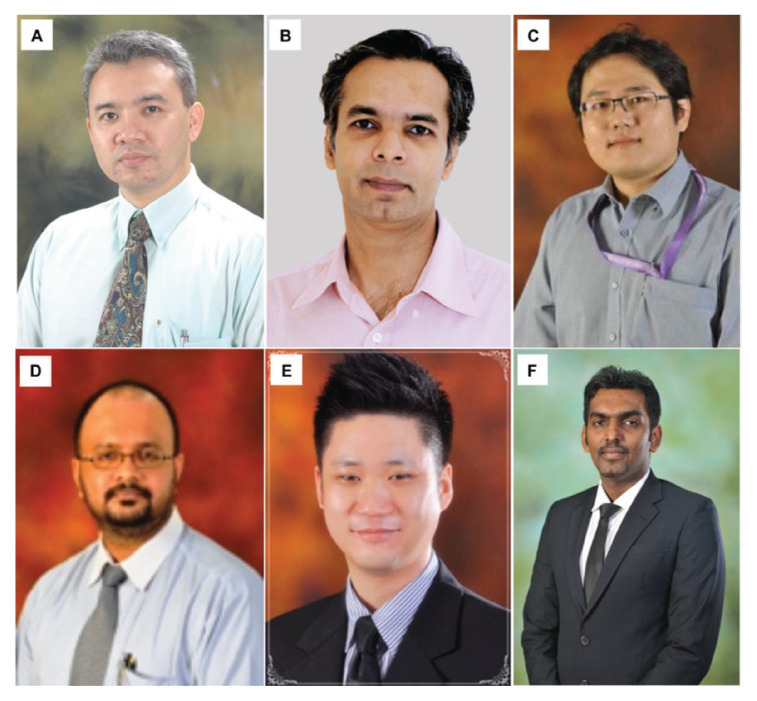
The current neurosurgeons in Penang General Hospital. (A) Dr Nasser Abdul Wahab (2014–2020), (B) Dr Azman Raffiq (current head of department), (C) Dr Ch’ng Chee How, (D) Dr Senthil Kumar a/l Rajapathy, (E) Dr Kelvin Lim Liang Hooi and (F) Dr Kumarappan a/l Chokalingam

**Figure 59:**
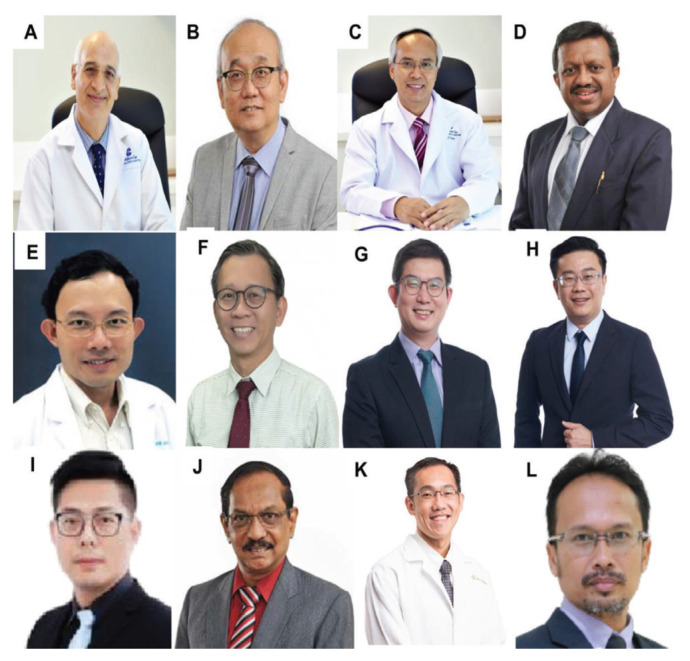
Neurosurgeons in major private hospitals in Pulau Pinang. (A) Dr Kazem Djavadkhani, (B) Dr Yoong Meow Foong, (C) Dr Mohd Supion Hj Dimin, (D) Dr K Ravindran a/l Katheerayson, (E) Dr Ng Cheok Man, (F) Dr Mathew Tung Yu Yee, (G) Dr Kan Choon Hong, (H) Dr Gee Teak Seng, (I) Dr Tan Yew Chin, (J) Dr B Gunasekaran a/l VJ Balasundaram, (K) Dr Lee Hock Keong and (L) Dr Ahmad Zamzuri Remeli

Dr Yoong was succeeded by Dr Azmin Kass Rosman, who served until 2005 when he was transferred to Sungai Buloh Hospital in Selangor (Figures 43A, 45 and 56). His post was taken over by Dr Ravindran Katheerayson from 2005 to 2008 (Figure 57). Dr Gurmit Singh (Figure 41C) continued as the head from 2008 to 2014. Dr Nasser Abdul Wahab (Figure 58A), a local graduate of the Master of Neurosurgery USM programme, took over the helm in 2014. He moved in 2020 after being appointed as the sole neurosurgeon at Putrajaya Hospital. The current department head is Dr Azman Raffiq (Figure 58B), assisted by four other neurosurgeons — Dr Ch’ng Chee How (Figure 58C), Dr Senthil Kumar a/l Rajapathy (Figure 58D), Dr Kelvin Lim Liang Hooi (Figure 58E) and Dr Kumarappan a/l Chokalingam (Figure 58F). Dr Kumarappan just completed his Master in Neurosurgery USM programme in 2021 and been posted to Penang General Hospital. There are currently 11 resident neurosurgeons in major private hospitals in Pulau Pinang (Figure 59).

### Neurosurgery in Perak

**Figure 60:**
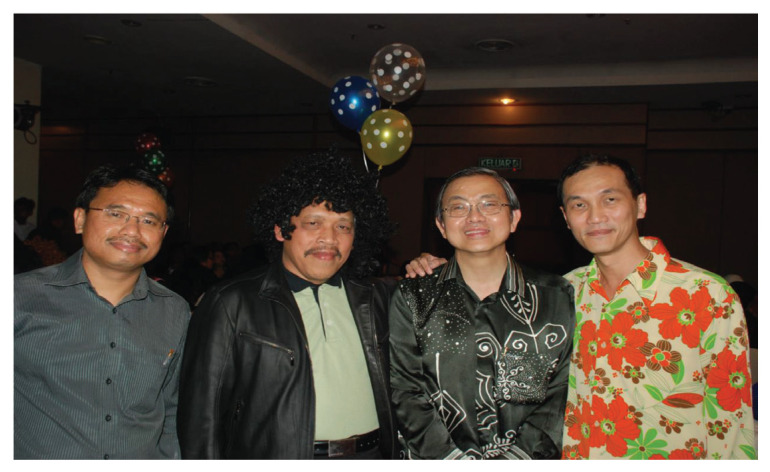
Event in 2009: Ipoh Neurosurgical Nite Dinner. From left to right; Dr Azmi Alias, Dr Mohammed Saffari Haspani, Dr Fadzli Cheah Abdullah and Dr Cheang Chee Keong

Neurosurgery service was first available in the state of Perak when Dr Fadzli Cheah Abdullah (Figures 39 and 60) joined the Ipoh Specialist Centre (currently known as the KPJ Ipoh Specialist Hospital) on 1 January 1990. He was the first neurosurgeon and for six years until 1996, the only neurosurgeon for the State of Perak. In addition to his busy private practice, Dr Fadzli Cheah Abdullah also provided voluntary service in his capacity as Honorary Consultant to the Ipoh General Hospital (currently known as Hospital Raja Permaisuri Bainun) during this early period. Dr Fadzli Cheah Abdullah also served as the medical director of the KPJ Ipoh Specialist Hospital from 2007 to 2017.

**Figure 61:**
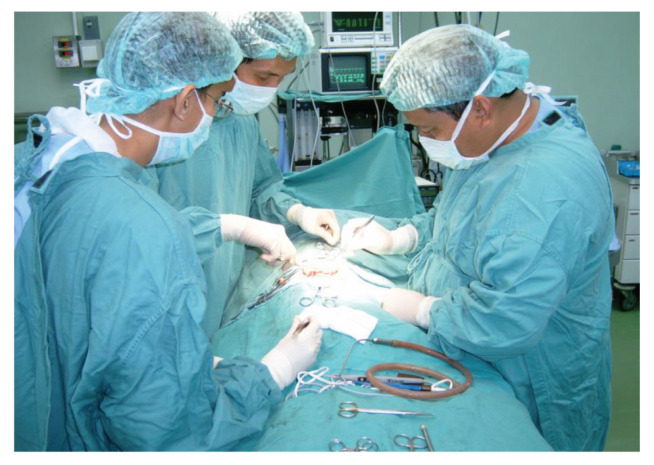
Photo in 2002 where Dr Mohammed Saffari Haspani and Dr Cheang Chee Keong were performing spine surgery

**Figure 62:**
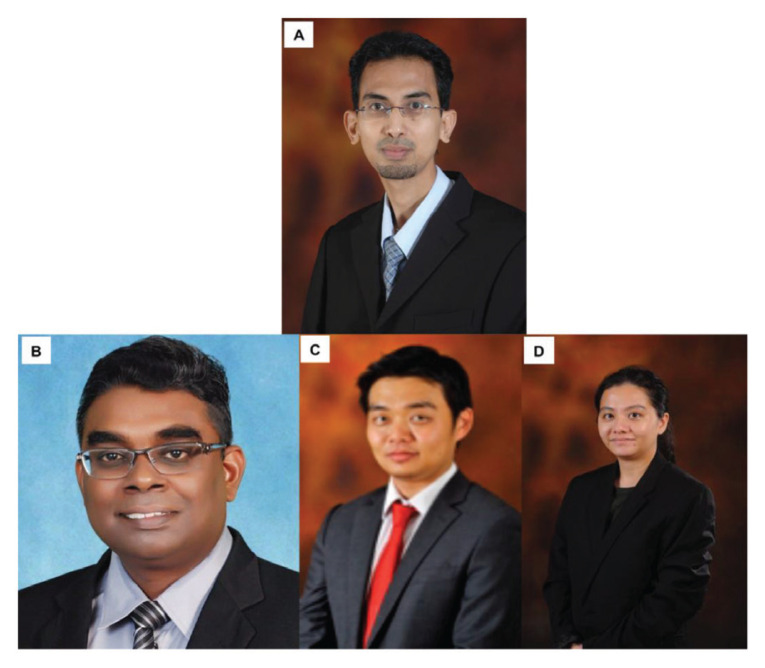
Current neurosurgeons at Hospital Raja Permaisuri Bainun. (A) Dr Mohamad Azhari Omar, (B) Dr Premananda Raja a/l Murugesu, (C) Dr Mohd Syahiran Mohd Sidek and (D) Dr Neoh Yee Yik

In 1996, Dr Mohammed Saffari Haspani (Figures 39, 40B and 61) was posted to Hospital Raja Permaisuri Bainun and served as head of the department until 2003. Several neurosurgeons provided their services at different times — Dr Baskaran a/l Suppiah (trained in Australia) from 1997 till 1999; Dr Ravindran Katheerayson (Figures 21 and 41D) from 2001 till 2004; and the late Dr Anil a/l K Sivasankaran from 2005 till 2006. Dr Cheang Chee Keong took over as head of the department in 2007 after completing his neurosurgery training under Professor Andrew Kaye in Melbourne, Australia. Dr Cheang Chee Keong resigned in 2016 and joined the KPJ Ipoh Specialist Hospital. He was succeeded by Dr Mohamad Azhari Omar (Figure 62A), the current department head, assisted by three other neurosurgeons—Dr Premananda Raja a/l Murugesu (Figure 62B), Dr Mohd Syahiran Mohd Sidek (Figure 62C) and Dr Neoh Yee Yik (Figure 62D).

**Figure 63:**
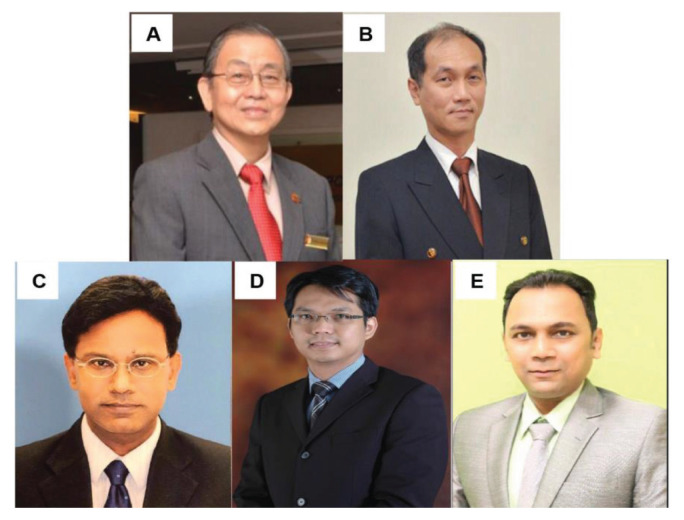
Neurosurgeons in major private hospitals in Perak. (A) Dr Fadzli Cheah Abdullah, (B) Dr Cheang Chee Keong, (C) Dr Baskaran a/l Suppiah, (D) Dr Tan Wei Ming and (E) Dr Jason Raj a/l Johnson Kovilpillai

In the private sector, besides Dr Fadzli Cheah Abdullah (Figure 63A) and Dr Cheang Chee Keong (Figure 63B) in the KPJ Ipoh Specialist Hospital, there are currently two neurosurgeons — Dr Baskaran a/l Suppiah (Figure 63C) in Fatimah Hospital and Dr Tan Wei Ming (Figure 63D) in Pantai Ipoh Hospital — and one, Dr Jason Raj a/l Johnson (Figure 63E) in Columbia Asia.

### Neurosurgery in Kedah

**Figure 64:**
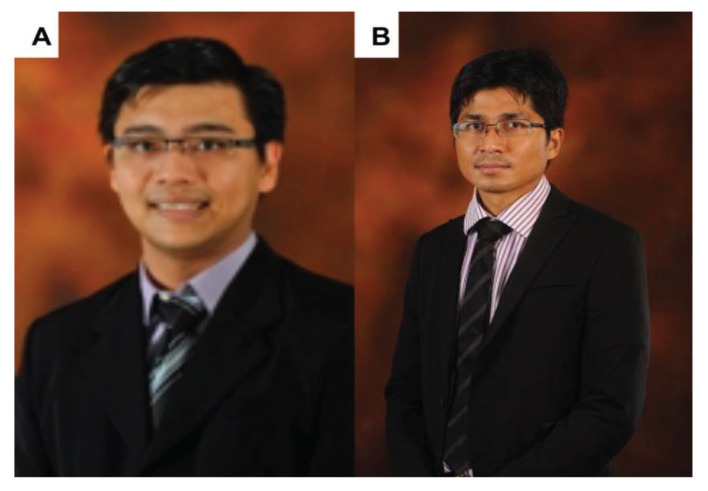
The present neurosurgeons at Hospital Sultanah Bahiyah. (A) Dr Lai Chuang Chee and (B) Dr Asrarul Fikri Abu Hassan

**Figure 65:**
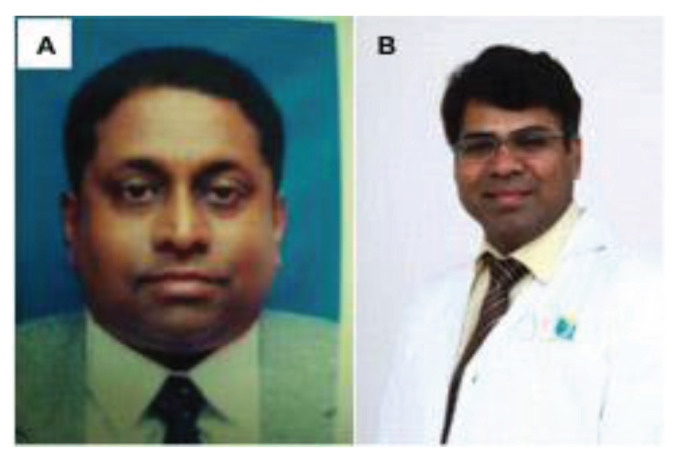
The previous and current neurosurgeons in private sector in Kedah. (A) The late Dr Anil a/l K Sivasankaran (B) Dr Daniel Rajesh Babbu

The state general hospital, Hospital Sultanah Bahiyah, initiated neurosurgical service on 9 April 2011 when Dr Adam Mohd Zakaria was posted there. Dr Adam Mohd Zakaria is a local USM graduate who previously worked in Ipoh General Hospital and Penang General Hospital after completing his neurosurgery training. He was the sole neurosurgeon for the state of Kedah and was transferred to Sibu General Hospital on September 2016. He currently works at HKL (Figure 41B). He was succeeded by Dr Ahmad Zamzuri Remeli (Figure 59L), also a graduate from the Master of Neurosurgery USM programme. Later, Dr Lai Chuang Chee (Figure 64A) took over in 2019 after Dr Ahmad Zamzuri Remeli joined KPJ Pulau Pinang in private practice. Dr Asrarul Fikri Abu Hassan soon joined the service in 2021 (Figure 64B). In the private sector, after the demise of the late Dr Anil a/l K Sivasankaran (Figure 65A), there has been only one neurosurgeon, Dr Daniel Rajesh Babbu (Figure 65B).

## Neurosurgery in the Eastern Region

### Neurosurgery in Pahang

Neurosurgery service was first available in Kuantan, Pahang, in 2004, when the IIUM recruited Dr Kyaw Tun Wai as a lecturer in the Surgery Department. Dr Kyaw Tun Wai was from Burma (Myanmar) and trained in general surgery and neurosurgery. He worked in close collaboration with the General Surgery Department of the State Hospital —HTAA — and provided neurosurgical services mainly dealing with head injury. In 2009, Dr Sani Sayuti (Figures 38A and 55D) joined IIUM, prior to embarking into the private sector. He was followed by Dr Saufi Awang (Figure 38B) and Dr Chan Kin Hup (Figures 38C and 55F) in 2010.

**Figure 66:**
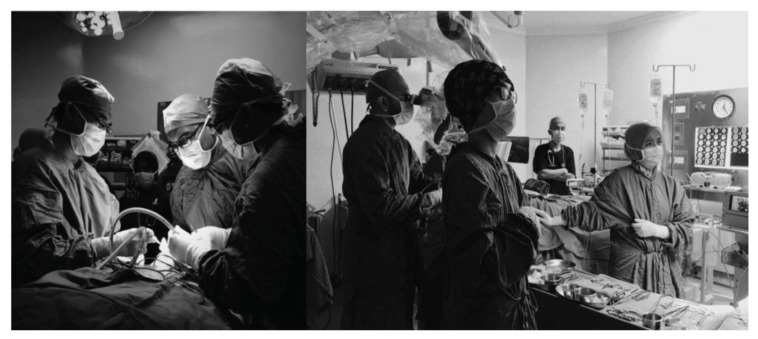
Dr Gerald Arvind Martin and the operating theatre staff at Hospital Tengku Ampuan Afzan during two separate surgeries

**Figure 67:**
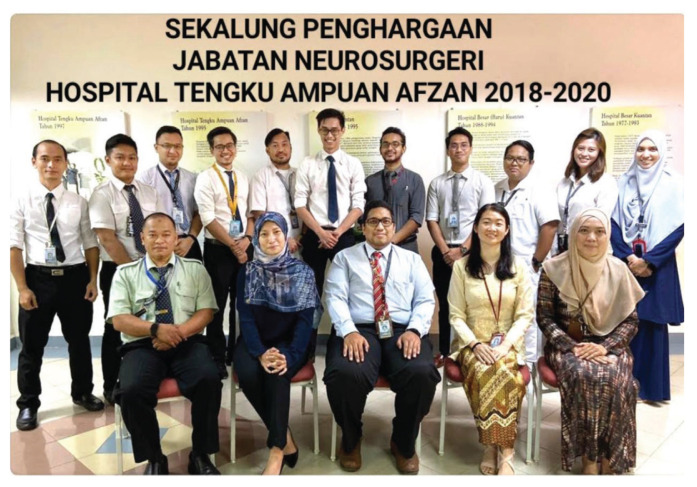
The current neurosurgical team at Hospital Tengku Ampuan Afzan. Second, seated from left are Dr Ailani Abdul Ghani, Dr Mohd Aidil Mohd Nor and Dr Low Siaw Nee

**Figure 68:**
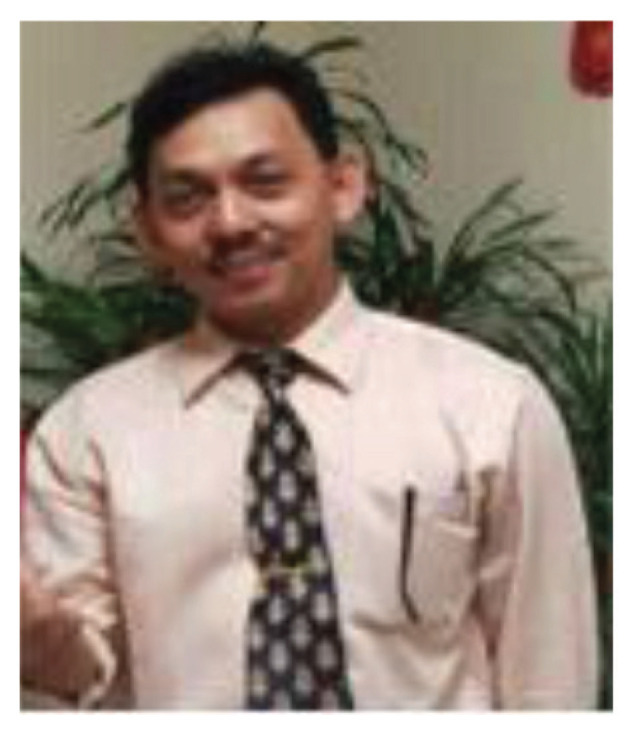
Dr Khairul Muhsein Abdullah is practicing in private sector in Pahang

In 2015, the MOH Malaysia appointed two neurosurgeons — Dr Gerald Arvind Martin (Figure 66) and Dr Mohd Aidil Mohd Nor (Figure 67, seated, middle), both from the Master in Neurosurgery USM programme — to set up a neurosurgery unit at HTAA, which was subsequently upgraded as an independent department in 2016. Treatment was further extended into emergency and elective cases. Dr Gerald Arvind Martin recently moved to private practice in 2019 (Figure 42T). The department was further strengthened with the addition of two neurosurgeons, Dr Ailani Abdul Ghani and Dr Low Siaw Nee (Figure 67). Neurosurgery service is also available in the private sector with Dr Khairul Muhsein Abdullah (Figure 68) serving at Kuantan Specialist Hospital (currently in KPJ Pahang Specialist Hospital) since 2008.

### Neurosurgery in Terengganu

**Figure 69:**
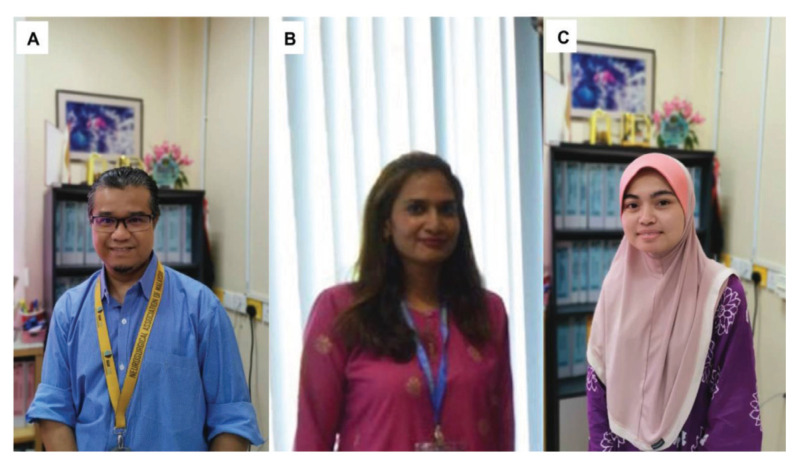
The department is run by (A) Dr Nujaimin Udin, assisted by (B) Dr Jacintha Vikeneswary Francis and (C) Dr Shukriyah Sulong

**Figure 70:**
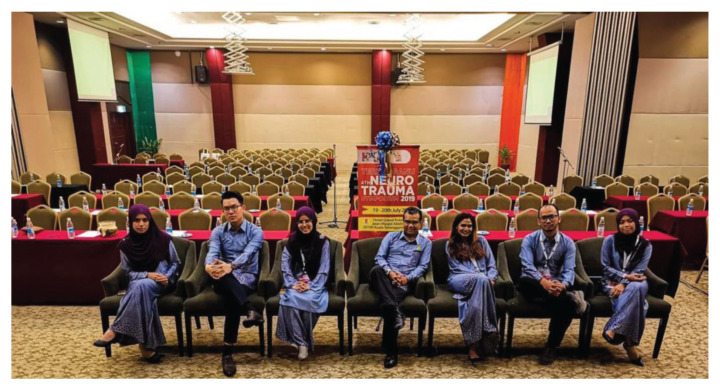
The 4th Neurotrauma Symposium 2019 in Terengganu. In the middle is Dr Nujaimin Udin and to his left is Dr Jacintha Vikeneswary Francis

Neurosurgery services under the MOH officially started on 30 March 2011 in the state of Terengganu when Dr Nujaimin Udin (Figure 69A) was appointed as the head of the Neurosurgery Department in Hospital Sultanah Nur Zahirah (HSNZ), Kuala Trengganu. Prior to that, neurosurgical cases were largely managed by general surgeons with consultation from Dr Ahmad Zubaidi Abdul Latif (Figure 39), professor and consulting neurosurgeon from the Universiti Sultan Zainal Abidin (UNISZA). Dr Nujaimin Udin is a local USM graduate. He is currently assisted by two female neurosurgeons, also USM graduates — Dr Jacintha Vikeneswary Francis (Figure 69B) and Dr Shukriyah Sulong (Figure 69C). The department has conducted many educational events and recently successfully organised the 4th Neurotrauma Symposium 2019 (Figure 70).

## Neurosurgery in East Malaysia

### Neurosurgery in Sarawak

**Figure 71:**
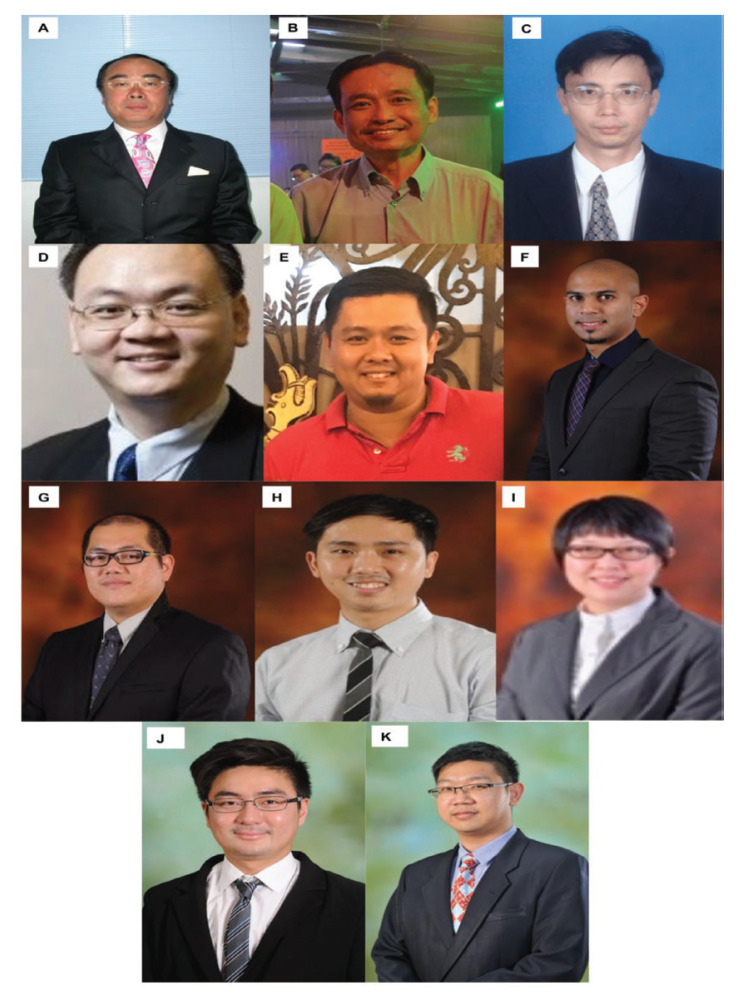
The previous and current neurosurgeons in Sarawak General Hospital (A) Professor Yuji Asou from Tokyo University, (B) the late Dr Ching Hin San, (C) Dr Albert Wong Sii Hieng, (D) Dr Donald Liew Ngian San, (E) Dr Lim Swee San, (F) Dr Davendran Kanesan, (G) Dr Lau Bik Liang, (H) Dr Goh Chin Hwee, (I) Dr Low Yong Lee, (J) Dr Teo Eu Gene and (K) Dr Sam Joe Ee

The neurosurgical service in Sarawak began following the arrival of Professor Torao Fuji at Sarawak General Hospital (SGH) on 9 April 1988, under the auspices of the Japan International Cooperation Agency (JICA). Professor Torao Fuji brought along not only his personal expertise, but also generous donations from his government of essential neurosurgical equipment, including an operating microscope, a craniotomy drill, an operating table and microsurgical instruments, to name just a few. The emergency service of the Accident and Emergency Department also benefited from the donation of an ambulance. Professor Torao Fuji left on 2 April 1990. He was succeeded by Professor Yuji Asou (Figure 71A) who served SGH from 18 August 1992 to 16 August 1994. Professor Yuji Asou returned again to serve another two-year term from 1996 to 1998. These two unassuming professors from Japan were pioneers in neurosurgery who laid the foundation for neurosurgery in Sarawak. Their services and contributions shall be remembered forever.

Upon his return from training in the Institute of Neurosciences, Glasgow, Scotland, and in Cambridge, England, Dr Ching Hin San (Figure 71B) was the first Malaysian neurosurgeon to serve at SGH. He left government service and joined the Normah Specialist Medical Centre in 1999. He passed away in 2016 after a brief illness. Dr Albert Wong Sii Hieng (Figure 71C), trained under Professor Peter Reilly in Royal Adelaide and Flinders Hospital, Australia, took over the helm of neurosurgery at SGH in 2000. He also completed a one-year spine fellowship programme with Professor Michael Fehlings in Toronto Western Hospital, Canada. Dr Albert Wong successfully established a very active service and training centre for neurosurgery and the spine in East Malaysia. He is assisted by Dr Donald Liew Ngian San (Figure 71D), who joined him in 2006 after completing his neurosurgery training in Royal Perth and Sir Charles Gardner Hospital under Professor Neville Knuckey. Subsequently, Dr Lim Swee San (Figure 71E) joined in 2013. There are currently nine neurosurgeons at SGH.

**Figure 72:**
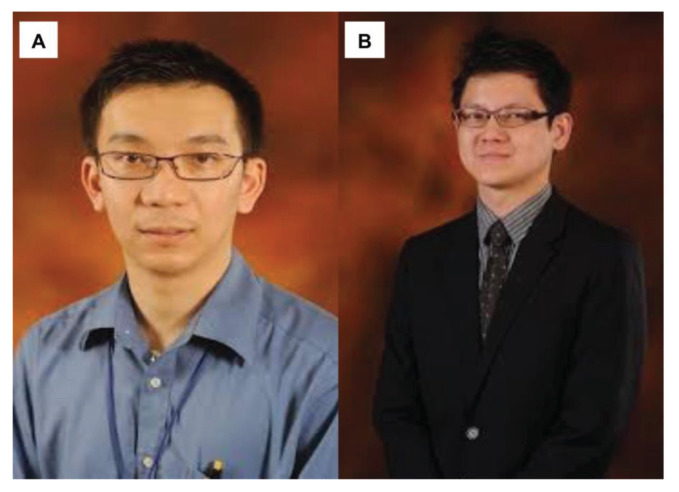
Current neurosurgeons in Sibu Hospital. (A) Dr Giat Seng Kho and (B) Dr Nelson Yap Kok Bing

**Figure 73:**
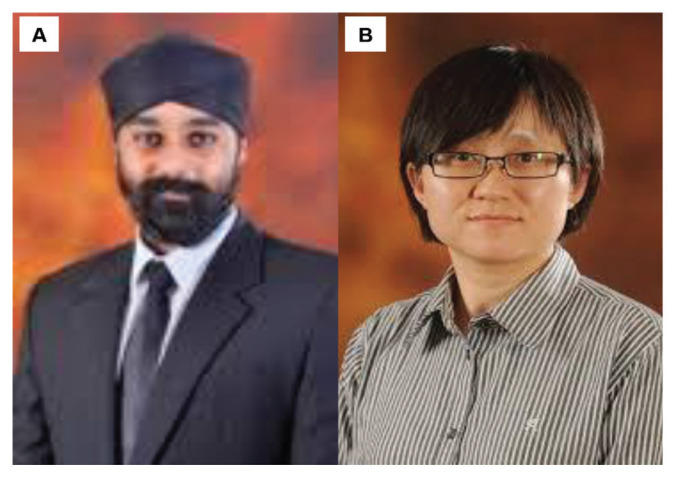
Current neurosurgeons at Miri Hospital. (A) Dr Manvinder Singh Mangat and (B) Dr Low Peh Hueh

**Figure 74:**
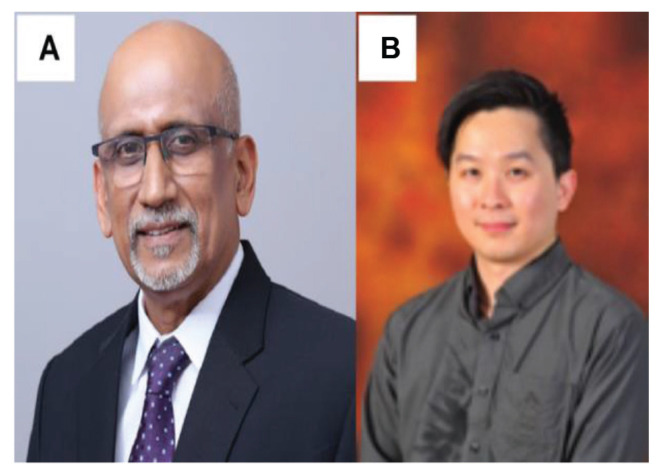
The neurosurgeons in private sector in Sarawak. (A) Dr Haroon M Pillay and (B) Dr Adrian Ng Wei Chih

Over the last 20 years, the department has been academically oriented, producing 19 scientific papers in various national and international journals on topics ranging from cerebral aneurysm, acoustic neuroma and carpal tunnel syndrome. It also reported the first case of a brain stem auditory implant in Malaysia. The neurosurgical service expanded to other parts of Sarawak, including Sibu Hospital and Miri Hospital. In Sibu, the first neurosurgeon was Dr Premananda Raja a/l Murugesu (2013–2014), followed by Dr Arshad Ali (2014–2016). From 2017 until 2019, the hospital received two neurosurgeons — Dr Adrian Ng Wei Chih and Dr Adam Mohd Zakaria. Currently, the department is run by Dr Giat Seng Kho and Dr Nelson Yap Kok Bing (Figure 72). They are both local Neurosurgery USM graduates. Dr Albert Wong and Dr Donald Liew are also scheduled to visit the neurosurgical clinic. In Miri, the neurosurgery unit started 3 years ago (2018), with the first neurosurgeon, Dr Manvinder Singh Mangat (Figure 73A). A year later, he was joined by Dr Low Peh Hueh (Figure 73B). Both are local Neurosurgery USM graduates. Dr Albert Wong, Dr Donald Liew and Dr Lim Swee San take turns visiting the neurosurgical clinic. Under the Miri catchment area, a neurosurgical clinic was opened at Limbang Hospital and run by Dr Manvinder Singh. In private practice, Dr Haroon Manadath Pillay used to work at Normah Medical Specialist Centre in Kuching before returning to India (Figure 74A). There is only one neurosurgeon in private practice, Dr Adrian Ng Wei Chih (Figure 74B), currently at the Borneo Medical Centre.

### Neurosurgery in Sabah

**Figure 75:**
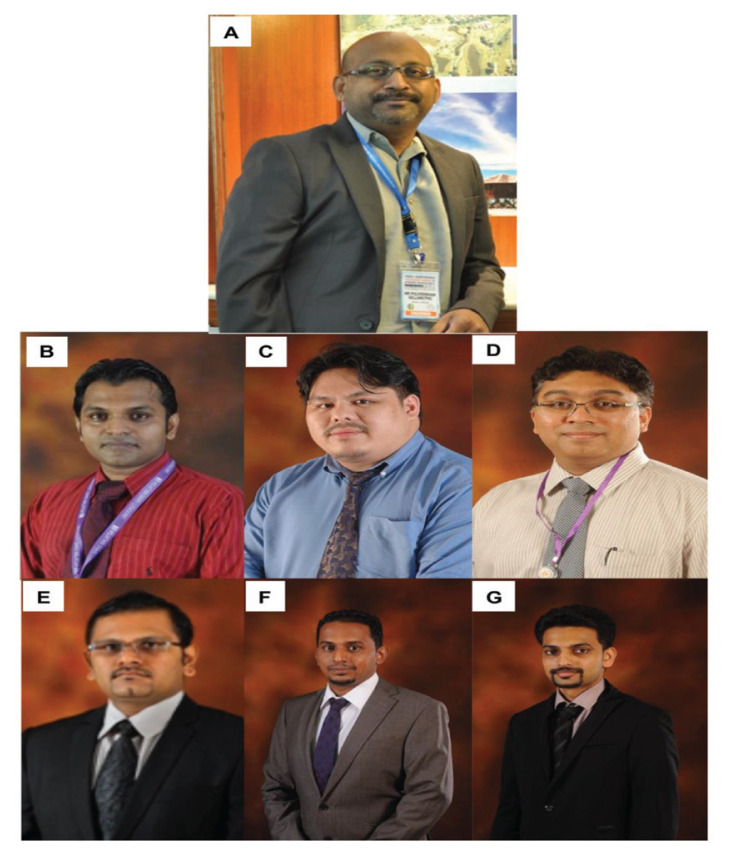
The current neurosurgeons in Sabah. (A) Dr Pulivendhan Sellamuthu, (B) Dr Ananda Arumugam, (C) Dr Sofan Zenian, (D) Dr Ramani a/l Thiagarajah, (E) Dr Prabu Rau a/l Sriram, (F) Dr Vinodh a/l Vayara Perumall and (G) Dr Ramissh Paramasivam

The first neurosurgery unit in Sabah was officially established at Queen Elizabeth Hospital (HQE) in February 2003. The unit was headed by Dr Pulivendhan Sellamuthu (Figure 75A), who was trained in the UK and obtained his FRCS at Edinburgh. He was trained in neuro-spine surgery at Addenbrooke’s Hospital, Cambridge. At the time of its formation, the department’s total strength was one neurosurgeon with two medical officers and two medical assistants. From this humble beginning of a 12-bed mixed ward in HQE, Dr Pulivendhan Sellamuthu has successfully transformed the unit to a modern neurosurgery department. The second neurosurgeon only came in 2007. With extra hand, the paediatric neurosurgery department expanded next to the Sabah Women and Children’s Hospital (SWACH) in 2007, the first such hospital in Malaysia. Currently, there are six other neurosurgeons covering three hospitals in Kota Kinabalu — HQE, Hospital Queen Elizabeth 2 (HQE2) and SWACH. They are Dr Ananda Arumugam (Figure 75B), Dr Sofan Zenian (Figure 75C), Dr Ramani a/l Thiagarajah (Figure 75D), Dr Prabu Rau a/l Sriram (Figure 75E), Dr Vinodh a/l Vayara Perumall (Figure 75F) and Dr Ramissh Paramasivam (Figure 75G). Dr Prabu Rau was sent to Oxford, UK and returned after completing his fellowship in Skull Base and Pediatric Neurosurgery in Oxford University Hospital. The department was named Sabah Brain and Spine Centre in August 2019 as a unified name for the departments of neurosurgery in all three hospitals in Kota Kinabalu and in recognition of the excellent services provided to the state. Currently, modern neurosurgical equipment and operating facilities have actively been made available to support maximum care to patients.

**Figure 76:**
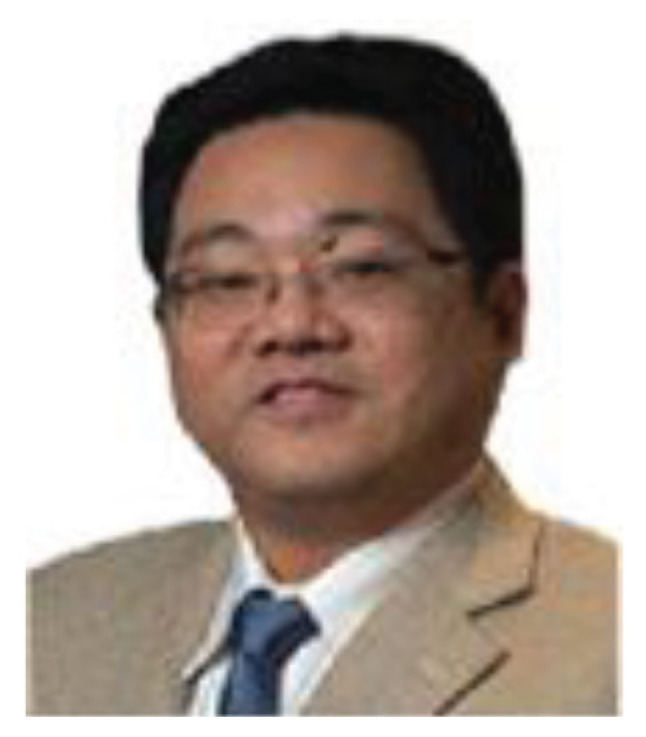
Dr Tan Wei Chean is the only neurosurgeon in private sector in Sabah

The department is active in teaching and training and plays an important role in the Master of Neurosurgery USM postgraduate programme. To date, it has produced six local neurosurgeons and has taken in eight international doctors for clinical attachment. The department currently has six neurosurgeons and six trainee residents. They visit clinics in remote hospitals and provide flying neurosurgical operative services to hospitals such as Lahad Datu, Sandakan, Tawau, Labuan and Keningau. This is the regional referral centre for a population of almost four million people served. There are active research activities with several journal publications, regular neurosurgical updates and conferences. In the private sector, there is only one neurosurgeon, Dr Tan Wei Chean (Figure 76).

## Neurosurgical Association of Malaysia (NAM)

Neurosurgeons in Malaysia, as a fraternity, have progressed exponentially in numbers, competence and technological advances since the inception of the first neurosurgical centre in Kuala Lumpur in 1963. It started with one expatriate neurosurgeon serving a population of nine million, to the mid-1980s with about seven neurosurgeons for 15 million population. The year 2001 saw the induction of Malaysia into the WFNS as a member society, with its then 21 neurosurgeons serving a population of 23 million.

On 21 February 2000, a group of enthusiastic neurosurgeons met in Kota Bharu, Kelantan and proposed the idea of establishing a national association of neurosurgeons. There was an imperative need for a national body to spearhead and coordinate neurosurgical activities in the country. The USM, under the initiative of Dr Jafri Malin Abdullah, was planning to launch the first local neurosurgery postgraduate training programme in 2001. It was thus vital to have the unanimity of support, endorsement, and collaboration from a singular national association that represented all the neurosurgeons in Malaysia. Dr Fadzli Cheah Abdullah accepted the nomination to be chairman of the Pro Tem Committee, with Dr Jafri Malin Abdullah as secretary tasked with drafting the constitution for the proposed association.

**Figure 77:**
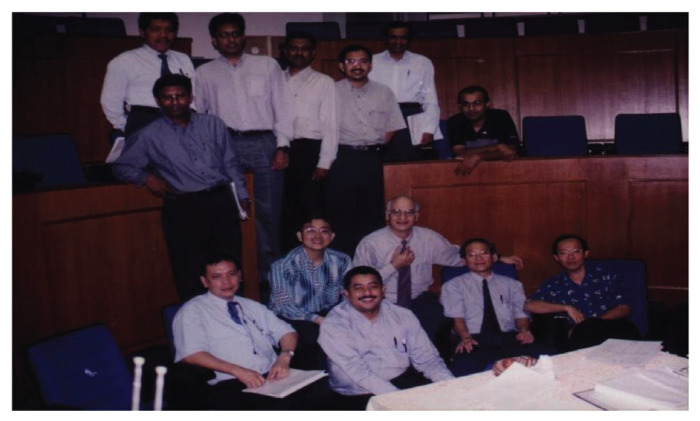
The First Inaugural Annual General Meeting of Neurosurgical Association Malaysia was held at the Shangri-La Hotels and Resorts, Kuala Lumpur on 16 June 2001

On 23 February 2001, this Pro Tem Committee convened at the Shangri-La Hotel in Kuala Lumpur with the participation of 17 neurosurgeons. The decision to set up a national association was well received and unanimously approved. The draft of the constitution prepared by Dr Jafri Malin Abdullah was adopted with some amendments. On 26 April 2001, the Neurosurgical Association of Malaysia (NAM) was officially registered with the Registrar of Societies (RoS), with special mention of the untiring efforts and tenacity of the Pro Tem Secretary. The first Annual General Meeting of NAM was held on 16 June 2001 (Figure 77). The following office holders were duly elected:

President: Dato’ Dr Nadasan Arumugasamy

Deputy President: Dr Fadzli Cheah Abdullah

Secretary: Dr Jafri Malin Abdullah

Treasurer: Dr Mohammed Saffari Haspani

Executive committee: Dr Richard Veerapan, Dr Muruga Kumar, Dr Zurin Adnan Abdul Rahman, Dr Johari Adnan Siregar

Auditors: Dr Kazem Djavadkhani, Dr Chee Chee Pin

In July 2001, NAM replaced the Malaysian Neurosciences Society (MSN) as the official affiliated member of the WFNS. On 16 September 2001, coinciding with a very significant day in our nation’s history, the NAM was officially inducted as a member society of the WFNS. On becoming a voting member society of the WFNS, the NAM actively participated in WFNS activities fostering bidirectional exchange of collaborative and mutually beneficial educational offerings. The first initiative was the WFNS Teaching Course, which was conducted in Kuala Lumpur in June 2003. The organisational task was entrusted to Dr Vicknes Waran and his UM team. This meeting attracted the participation of several prominent personalities and was the first of several WFNS teaching courses to follow. It saw the beginning of focused teaching with an emphasis on subspecialty topics, including paediatric neurosurgery and stereotactic radiosurgery. The post educational evenings included interaction and the exchange of ideas and innovations between senior members of the fraternity on the development of neurosurgery and neurosurgical education.

The NAM administration has had a succession of six further presidents to date, each of whose administrative terms comprised respective areas’ emphasis and niche developments. Dr Fadzli Cheah Abdullah succeeded Dato’ Dr Nadasan Arumugasamy as the second president of NAM following the third AGM on 18 May 2003. He was president for two terms to 2007. The administration by Dr Fadzli Cheah Abdullah oversaw the escalation and formalisation of neurosurgical training in Malaysia. The first batch of the Masters of Neurosurgery programme established at the USM graduated, giving the fraternity its first intake of fully locally trained neurosurgeons. His term also saw the success of the parallel pathway of the neurosurgical training programme at UM. In August 2002, Dr Vicknes Waran established a parallel training pathway for neurosurgical trainees at UM. Aspirants with a postgraduate qualification in general surgery, either a Master in Surgery or an FRCS (general surgery), were enrolled in a matching programme that entailed three years of training at UM, followed by a two-to three-year stint in an accredited training post in the UK or Ireland, culminating in the FRCS (Neurosurgery).

**Figure 78:**
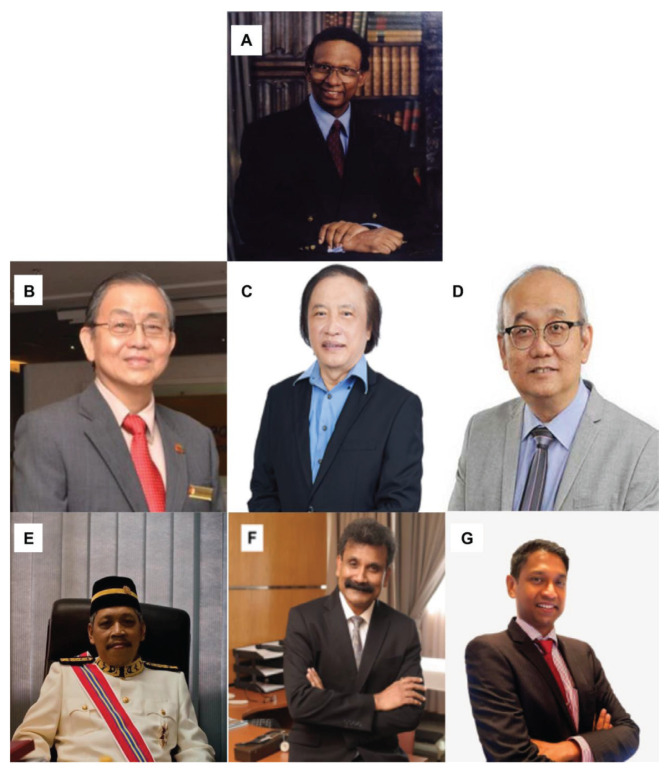
The past and current president of NAM. (A) Dato’ Dr Nadasan Arumugasamy (first president; 2001–2003), (B) Dr Fadzli Cheah Abdullah (second president; 2003–2007), (C) Dr Chee Chee Pin (third president; 2007–2011), (D) Dr Yoong Meow Foong (fourth president; 2011–2015), (E) Dr Mohammed Saffari Haspani (fifth president; 2015–2017), (F) Dr Hari Chandran Thambinayagam (sixth president; 2017–2019) and (G) Dr Kantha Rasalingam (seventh president; 2019–current)

The office of the president was subsequently led by its third president, Dr Chee Chee Pin, from 2007 to 2011; the fourth, Dr Yoong Meow Foong, from 2011 to 2015; the fifth, Dr Mohammed Saffari Haspani, from 2015 to 2017; the sixth, Dr Hari Chandran Thambinayagam, from 2017 to 2019; and the seventh and current president, Dr Kantha Rasalingam (Figure 78). NAM has systematically succeeded in uniting the Neurosurgery Fraternity and played a vital role in the promotion and development of neurosurgery in the country. The society has gone on to successfully organise numerous courses, seminars, workshops, and conferences on national, regional, and international platforms. NAM’s initiatives on the national level soon came to the fore, with particular emphasis on neurosurgical training. NAM then embarked on organising conferences on international platforms. During the presidency of Dr Chee, Malaysia hosted the 8th Asian Congress of Neurological Surgeons (ACNS) in Kuala Lumpur, Malaysia. This was the first international neurosurgical event of such magnitude organised and hosted by NAM. The event attracted the participation of more than 500 regional and international delegates. In addition to firmly establishing the society’s capability and distinction in organising a major meeting that was well appraised for its organisation and scientific content, the financial proceedings from this meeting contributed significantly to the coffers of the NAM and ensured its financial security and independence.

The administration of Dr Yoong Meow Foong saw the consolidation and further unification of various cohorts of the neurosurgical fraternity in Malaysia. This administration saw the beginning of a greater-than-linear increase in the number of neurosurgeons in Malaysia. Dr Yoong Meow Foong encouraged and supported the establishment of more neurosurgical training programmes. Dr Mohammed Saffari Haspani was elected president in August 2015. This administration was marked by a heterogeneous mix of committee members on the executive committee of the NAM. Office holders hailed from various cohorts of the neurosurgical fraternity — the MOH, private practitioners and the universities — with varying degrees of seniority. The Mohammed Saffari Haspani administration saw an exponential increase in the number of neurosurgeons in Malaysia. After a long hiatus, a combined neurology and neurosurgery conference titled ‘My neuro-reconnecting the synapses’ was held in August 2017. The conference enjoyed the participation of more than 924 delegates from the neuroscience’s community of Malaysia, which was the largest gathering to date of local neurologists and neurosurgeons. The event cemented the camaraderie between neurophysicians and ophthalmologists in Malaysia.

**Figure 79:**
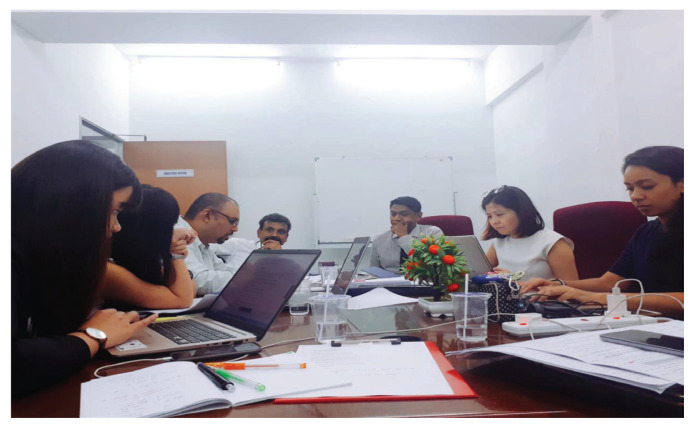
The Think Tank of the 6th International Symposia of the WFNS with the event organisers/conference partners

**Figure 80:**
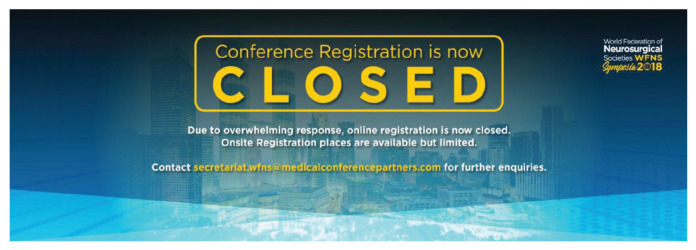
The end of the trepidation

Dr Hari Chandran became the sixth president of the NAM on 12 August 2017. His presidency heralded the ascension and recognition of NAM and neurosurgery in Malaysia in a global context via the WFNS. Dr Hari Chandran was elected as a member of the Constitution and Bylaws Committee of the WFNS during the XVI World Congress of the WFNS in Istanbul in August 2017. During this time, he secured the bid to host the VI International Symposia of the WFNS in Kuala Lumpur in August 2018 (Figures 79 and 80). There were many concerns and trepidations with regard to this undertaking. The Congress was to be sandwiched between the World Congress XV in Istanbul in August 2017 and the last interim meeting of the WFNS (termed Special World Congress) in Beijing in September 2019. Further, NAM was committed to giving the WFNS an honorarium of USD75,000 for hosting rights. With an excellent team passionately committed to success, this congress with six pre-congress workshops and three full days of scientific sessions managed to assemble many eminent and distinguished personalities of the neurosurgical universe to share updates and dynamic insights in the ever-evolving practice of neurological surgery; the Congress drew 957 delegates from more than 70 countries and attracted many corporate sponsors committed to neurosurgical education. The Congress was supported by all members of the WFNS administrative council and officers of the federation and representatives from all five Continental associations of the WFNS. The WFNS declared the meeting a success, surpassing all expectations and parallels were drawn to past and future World Congresses. The success of the meeting was also reflected in the net financial balance of NAM, which transcended all previous cumulative quantities. Dr Hari Chandran administration of 2017–2019 saw the conduct and support of NAM for numerous neurosurgical education endeavours by all the cohorts of the fraternity, notwithstanding the closer interaction among the same and incentives for the cohorts of Young Neurosurgeons (YNS) and Women in Neurosurgery (WINS). The formation of neurosurgical subspecialty committees within NAM was initiated, and good communication and progress was made with the MOH on the fee structure and with the Malaysian Medical Council on stringent credentialing of practitioners. In recognition of his contribution to the international community, Dr Hari Chandran was elected as the Continental Second Vice-President (Asia-Australasia) of the WFNS, further increasing the role of NAM in the world federation. As Dr Hari Chandran assumed his international duties, Dr Kantha Rasalingam took over the leadership of the NAM in 2019.

**Figure 81:**
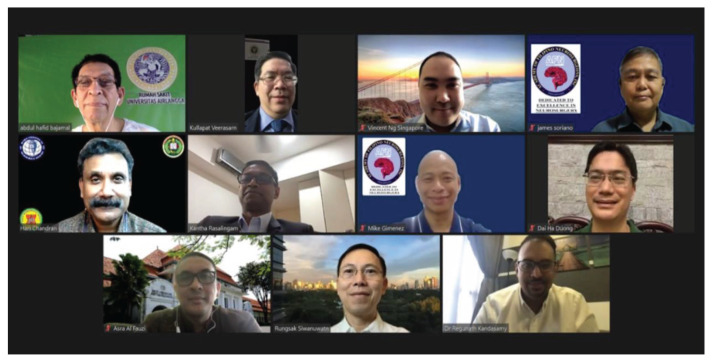
The ASEAN Neurosurgical Society virtual executive meeting

In his presidency, Dr Kantha was also elected to the Constitutions and Bylaws Committee of the WFNS in elections in Beijing. Dr Kantha was also elected president of the ASEAN Neurosurgical Society, while Dr Regunath Kandasamy became secretary of the ASEAN as well. Faced with unprecedented circumstances due to the COVID-19 pandemic, the NAM Exco made the shift into the virtual era by organising a series of virtual update webinars that culminated in the very first completely virtual congress of the ASEAN Neurosurgical Society, which incorporated the inaugural meeting of the second Vice Presidents of the WFNS (Figure 81). This event was a resounding success, with over 1,700 participants from nearly 116 countries. The organisation of this congress helped cement Malaysia’s versatile ability to organise events both physically and virtually. The secretary of NAM was also honoured by the WFNS Foundation, who named Dr Regunath Kandasamy in June 2021 as one of the Young Pioneers in developing neurosurgery education in the region.

NAM has been instrumental in the evolution and development of neurosurgery in Malaysia to its current form. The organisation endeavours to adhere to the highest standards and recommendations of practice and patient care. With its links and broad web of networking globally, training and advancement are ever at the fore. There is zero tolerance for compromises in training and patient care.

## Figures and Tables

**Figure 48:**
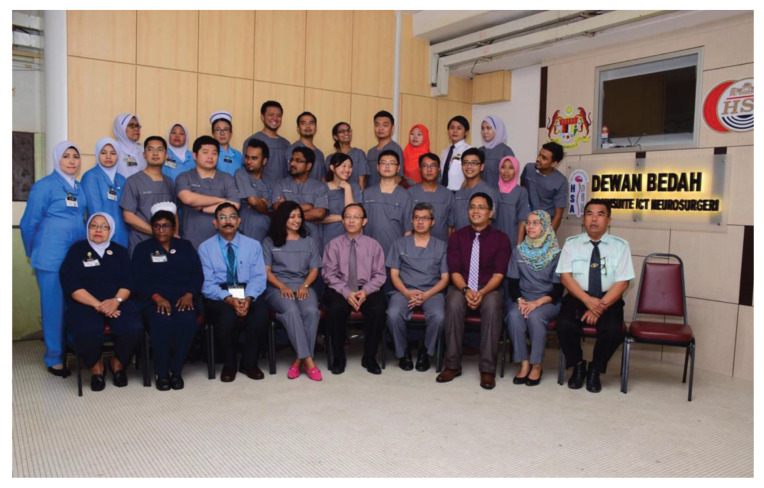
A group photo with Dr Johari Adnan Siregar who established the Brain Suite Intraoperative CT
